# Liquid Chromatography-Mass Spectrometry Analytical Methods for the Quantitation of *p*-Cresol Sulfate and Indoxyl Sulfate in Human Matrices: Biological Applications and Diagnostic Potentials

**DOI:** 10.3390/pharmaceutics16060743

**Published:** 2024-05-30

**Authors:** Ala’a R. Al-Dajani, Qi Kun Hou, Tony K. L. Kiang

**Affiliations:** Katz Group Centre for Pharmacy and Health Research, Faculty of Pharmacy and Pharmaceutical Sciences, University of Alberta, Edmonton, AB T6G 2E1, Canada; aaldajan@ualberta.ca (A.R.A.-D.); qikun@ualberta.ca (Q.K.H.)

**Keywords:** *p*-Cresyl sulfate, indoxyl sulfate, protein-bound uremic toxins, liquid chromatography-mass spectrometry, kidney disease

## Abstract

Indoxyl sulfate (IxS) and *p*-cresyl sulfate (*p*CS) are toxic uremic compounds with documented pathological outcomes. This review critically and comprehensively analyzes the available liquid chromatography-mass spectrometry methods quantifying IxS and *p*CS in human matrices and the biological applications of these validated assays. Embase, Medline, PubMed, Scopus, and Web of Science were searched until December 2023 to identify assays with complete analytical and validation data (N = 23). Subsequently, citation analysis with PubMed and Scopus was utilized to identify the biological applications for these assays (N = 45). The extraction methods, mobile phase compositions, chromatography, and ionization methods were evaluated with respect to overall assay performance (e.g., sensitivity, separation, interference). Most of the assays focused on human serum/plasma, utilizing acetonitrile or methanol (with ammonium acetate/formate or formic/acetic acid), liquid–liquid extraction, reverse phase (e.g., C18) chromatography, and gradient elution for analyte separation. Mass spectrometry conditions were also consistent in the identified papers, with negative electrospray ionization, select multiple reaction monitoring transitions and deuterated internal standards being the most common approaches. The validated biological applications indicated IxS and/or *p*CS were correlated with renal disease progression and cardiovascular outcomes, with limited data on central nervous system disorders. Methods for reducing IxS and/or *p*CS concentrations were also identified (e.g., drugs, natural products, diet, dialysis, transplantation) where inconsistent findings have been reported. The clinical monitoring of IxS and *p*CS is gaining significant interest, and this review will serve as a useful compendium for scientists and clinicians.

## 1. Introduction

Kidneys are responsible for filtering the blood of waste products such as endogenous uremic compounds, which are accumulated in patients with impaired renal function [1,2]. Chronic kidney disease (CKD) can cause the elevation of uremic toxins (UTs) [2] and affects around 37 million people in the United States and 8–16% of the population worldwide [2,3]. Unfortunately, 90% of adults with CKD are not aware of their diagnosis [3]. CKD is pathologically identified as the presence of one or more kidney dysfunction markers for a duration of three or more months of (i) estimated glomerular filtration rate (eGFR) less than 60 mL/min/1.73 cm^2^, (ii) increased albumin excretion rates up to 30 mg/24 h, or more, (iii) increased albumin-to-creatinine ratio up to 30 mg/g, or more, and (iv) others (e.g., hematuria, structural abnormalities) [4,5]. eGFR is an important marker commonly used for the classification of CKD stages, where normal kidney function (“G1”) is defined as eGFR ≥ 90 mL/min/1.73 cm^2^, CKD stage G2 (eGFR = 60–89 mL/min/1.73 cm^2^), CKD stage G3a (eGFR = 45–59 mL/min/1.73 cm^2^), CKD stage G3b (eGFR = 30–44 mL/min/1.73 cm^2^), CKD stage G4 (eGFR = 15–29 mL/min/1.73 cm^2^), and CKD stage G5 (eGFR < 15 mL/min/1.73 cm^2^) [4].

**Figure 1:**
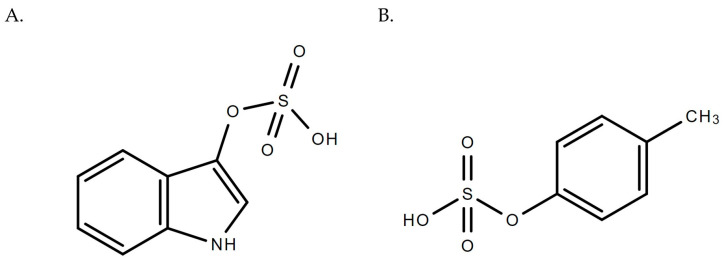
Chemical structures for (**A**) indoxyl sulfate and (**B**) *p*-cresyl sulfate.

The European Uremic Toxins Work Group (EUTox) identified a list of 130 UTs and provided a framework for classification [6,7,8,9]. The uremic toxins can be classified according to their physiochemical properties such as size and binding affinities [10]: (i) free water-soluble low molecular weight compounds (<0.5 kD), (ii) middle molecules (0.5–60 kD), and (iii) protein-bound uremic toxins (PBUTs) [7]. The latter class is known for its inefficient clearance during typical dialysis protocols [11,12]. In addition, Rosner et al. [13] suggested the use of a different classification system according to hemodialysis techniques, dialysis membranes, and patterns of solute removal [13]. Moreover, Rosner et al. [13] recommended the use of the 2018 classification, which relies on the biological outcomes of uremic toxins [6,13]. In this classification, the uremic toxins were given scores according to the reported experimental evidence on their toxicities [6]; for example, some small water-soluble solutes can have high toxicity scores [6]. Of interest to this review are the two sulfonated PBUTs, indoxyl sulfate (IxS) and *p*-cresyl sulfate (*p*CS), which have gained significant research interest in the last decade due to their toxicological impacts on serum accumulation (Figure 1). We refer the readers to several excellent reviews on the toxicology of IxS and *p*CS (e.g., [6,14,15,16,17,18,19]), which are known to be associated with cardiovascular outcomes in CKD patients [20], colonic cancer [15], oxidative stress [21], and the progression of CKD [17,19]. Recently, Vanholder et al. [6] have also ranked both IxS and *p*CS as uremic compounds of significant toxicological interest.

IxS, initially called “indican”, has a molecular weight of 213 g/mol, and its protein binding characteristics were reported to be 90% or more [19]. IxS remains at elevated concentrations even after dialysis due to its extensive protein-binding behavior [22]. IxS is produced endogenously from intestinal microflora that metabolizes tryptophan to form indole, which undergoes hepatic hydroxylation by CYP2E1 and conjugation by SULT1A1 to form IxS [14]. Similar to IxS, *p*CS (molecular weight 188.2 g/mol) is generated in the liver after the sulfonation of *p*-cresol (*p*C), a gut-derived metabolite of tyrosine and phenylalanine fermentation [17,23]. *p*CS binds to albumin (median percentage protein binding > 90%) and is extensively retained in the body in cases of abnormal low renal excretion [17,24]. Both IxS and *p*CS are excreted into the urine via renal tubular secretion [25]. In patients with renal dysfunction, the accumulation of IxS and *p*CS may lead to the manifestation of toxicities in various organ and tissue systems (e.g., [6,14,15,16,17,18,19]). Further examples include significant correlations observed between urine IxS concentrations and urinary oxidative stress markers such as 15-isoprostane F_2t_ and pteridine in patients with type 2 diabetes mellitus [26] and elevated plasma *p*CS concentrations being associated with ischemic strokes in hemodialysis patients [27]. Therefore, monitoring IxS and *p*CS concentrations can potentially aid the diagnosis, prevention, and management of uremia-induced pathophysiology. The literature provides various analytical techniques to quantify IxS and *p*CS, such as ultra-performance liquid-chromatography (UPLC), LC-MS/MS, and enzyme-linked immunosorbent assays (ELISA) (e.g., [28,29,30]). However, LC-MS/MS assays are considered the most sensitive and selective, with the ability to quantify IxS and *p*CS in the nanomolar concentrations (e.g., [31,32]).

Several excellent reviews have been published around these subject areas. Fernandes et al. provided an overview of various analytical assays separating IxS, *p*CS, *p*C, and indole-3-acetic acid (IAA) [8], focusing on a wide array of sample preparation, chromatographic, and detection methods [8]. Heaney [33] provided discussions on the principles and examples of mass spectrometry-based analytical assays to quantify gut-derived bacterial metabolites and a variety of classes of uremic toxins [33]. Niwa provided general discussions on mass-spectrometry data for both IxS and *p*CS [34,35]. The current critical review provides a comprehensive update with a focus on LC-MS/MS assays validated specifically for IxS and *p*CS in human clinical samples. Our review discusses in further detail all relevant assay parameters such as sample handling, matrix, chromatography, mass-spectrometry, and validation. Additionally, our review provides a critical analysis of the clinical applications related to these validated assays, with a focus on potential diagnostic applications. This review is tailored to pharmaceutical analytical scientists with interest in assay development and also to clinicians who are interested in managing the pathophysiology of IxS and *p*CS-associated uremic toxicities.

## 2. Materials and Methods

**Figure 2:**
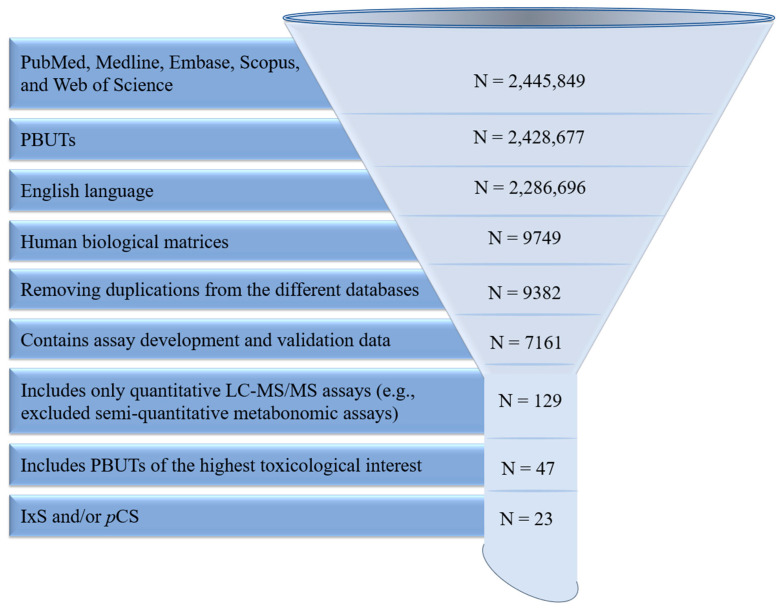
Literature screening, exclusion, and inclusion. PBUTs: protein-bound uremic toxins. LC-MS/MS: high-performance liquid chromatography-mass spectrometry. IxS: indoxyl sulfate. *p*CS: *p*-cresyl sulfate. N: total number of papers remaining. PBUTs of the highest toxicological interest were determined according to a recent critical review [6].

Embase, Medline, PubMed, Scopus, and Web of Science were searched until December 2023 for articles included in this review using combinations of the following keywords: liquid chromatography-mass spectrometry (LC-MS/MS), high-pressure liquid chromatography (HPLC), and protein-bound uremic toxins (PBUT). The initial search resulted in 2,445,849 papers, which was reduced to 2,428,677 papers that only focused on PBUTs. Of these, 2,286,696 papers were in English, from which we included 9749 papers that analyzed human biological matrices. Further exclusion criteria were (i) database duplications (N = 9382 remaining); (ii) lack a clear description of validation parameters as outlined in the United States Food and Drug Administration guidance document [36] (N = 7161 remaining); (iii) lack of quantitative mass-spectrometry detection (N = 129 remaining), and (iv) PBUTs not deemed of clinical toxicological interest based on a recent critical review [6] (N = 47 remaining). Of these, we further narrowed the focus to 23 papers specifically tailored to IxS and/or *p*CS (Figure 2). Data pertaining to the biological matrix, sample processing, chromatography parameters, mass spectrometry detection, and validation were extracted for further analysis.

**Figure 3:**
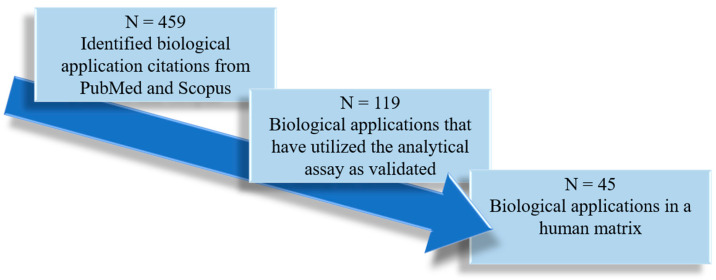
Literature screening, inclusion, and exclusion for the human biological applications of the validated high-performance liquid chromatography-mass spectrometry assays quantifying indoxyl sulfate and *p*-cresyl sulfate (in reference to Figure 2). N: total number of biological applications remaining.

The biological applications reported within these analytical assays were also objectively summarized and discussed in this review. Additional applications of these analytical methods were identified in PubMed and Scopus by systematic citation analysis (i.e., searching for indexed peer-reviewed papers that have cited these 23 analytical assays as they were originally validated). A total of 45 biological applications were identified and further categorized based on the human biological matrix. If the biological applications did not use the cited analytical assay as originally developed/validated, they were excluded from our analysis (Figure 3). Data pertaining to experimental objectives, population characteristics, and the authors’ key findings were extracted for analysis.

## 3. Results and Discussion

### 3.1. Analytical Assays in Serum and Plasma

Most of the methods that quantify IxS and/or *p*CS were validated in serum (N = 11), plasma (N = 5), or combined (N = 2). The majority of the assays quantified additional analytes (including IxS and/or *p*CS in the mix); however, some assays focused only on either IxS, *p*CS, or the combination of these two sulfonated metabolites [29,37,38,39] (Table 1).

#### 3.1.1. Sample Preparation

For assays in serum and/or plasma, the most common sample extraction procedure is liquid–liquid extraction (LLE), which includes the use of a protein precipitation solution typically containing the internal standards. The choice of the organic solvent depended mainly on the organic phase used in the liquid chromatography mobile phase and consisted primarily of acetonitrile (ACN) and/or methanol. Both IxS and *p*CS contain aromatic rings (i.e., indole and benzene) and a polar sulfonate functional group; therefore, methanol and ACN could possibly provide both dispersion [59] and electrostatic interactions [60], supporting their common usage in sample extraction (Table 1).

To our knowledge, the extraction efficiency of the different organic solvents for IxS and *p*CS has not been systematically tested. However, ACN appears to be utilized in more analytical assays (Table 1), and these assays had slightly longer run times (e.g., 2.5–20 min, Table 1) than assays that used methanol (e.g., 6–10.1 min, Table 1) when data were provided. With respect to assay sensitivity, the use of ACN for the extraction of serum and plasma was associated with LLOQs of ~20–500 ng/mL [37,46,50], whereas methanol was associated with LLOQs of the most sensitive assays being 1 ng/mL for IxS and *p*CS in serum [31,32] and ~50 ng/mL for IxS in plasma [51]. Different approaches were used to measure the matrix effects (e.g., with/without sample extraction, the choice of the blank matrix/neat solution) (Table 1) with inconsistencies across the studies; therefore, it may not be possible to compare the results directly between different papers. Based on Table 1, the use of ACN was accompanied by a matrix effect of <20% for IxS and *p*CS in most of the assays (e.g., [44,46]). However, a significant matrix effect of more than 20% was reported by André et al. [45], whereas methanol also had a matrix effect <20% for IxS and *p*CS [31,51], except for Monosik et al. [49]. Overall, there is no consistent pattern in the usage of a particular solvent for the quantification of IxS and/or *p*CS, and no clear trends in recovery efficiency were associated with a specific organic solvent, where 80–120% has been reported for IxS or *p*CS recovery in ACN and in methanol except for Wang et al. [47] and Zhu et al. [32] which reported the recovery outside of this range. On the other hand, both ACN- and methanol-processed samples can be placed at –20 °C or –80 °C for long-term storage, with most assays being stable up to three freeze–thaw cycles for both organic solvents (Table 1). An exception was reported for IxS stability after three freeze–thaw cycles performed by André et al. [45]. While there is no clear advantage of one organic solvent over the other, it should be cautioned, however, that these comparisons are only qualitative in nature as a properly controlled experiment is required to test the overall effects of ACN vs. methanol for extraction.

On the other hand, solid-phase extraction (SPE) was also used in some assays [43,51], which typically requires additional hardware, such as the Oasis WAX μElution plate [51] and the 3cc HLB cartridge [43]. In Oda et al. [51], the Oasis WAX μElution plate is pre-conditioned with methanol and water, the plasma sample is loaded into the plate after being mixed with the working solution, the plate is washed with 2% aqueous formic acid, and the analytes are eluted with 0.25% aqueous ammonia solution in methanol. This protocol generated a linearity range of 0.05–200 μg/mL for IxS with an extraction recovery corrected to the internal standards as 100.7%, 101.9%, and 101.6% for 0.15, 4.5, and 150 μg/mL, respectively [51]. In Zhang et al. [43], serum samples were loaded into a 3cc HLB cartridge conditioned with methanol and formic acid (1%), the cartridge washed with formic acid (1%), and the analytes eluted with ammonium peroxide (1%) in methanol: water (50:50 *v*/*v*%). The supernatant is then evaporated with an N_2_ stream and subsequently reconstituted with ammonium acetate (5 mM). This method generated a linearity range between 0.021–106.605 μg/mL for IxS and an extraction recovery of 108.7% and 115.3% for 33.3 and 10 µmol, respectively [43]. Based on the limited available data, it is difficult to determine which SPE protocol provided better assay performance, and it is not clear whether these protocols could also be applied to *p*CS. The LLE also does not require specific extraction cartridges, which may result in more cost savings.

To ensure efficient extraction, various mechanical means (e.g., incubation, vortex mixing, ultrasonication, and centrifugation) are utilized for sample cleanup and protein removal (Table 1). Most assays that used methanol for extraction tended to incubate the sample with the extraction solution (15–60 min) [32,38,49], but this approach was not commonly performed with ACN. This might be due to the C≡N group in ACN, which is a stronger nucleophile compared to the OH group, thereby minimizing the required contact time. On the other hand, some assays used a longer vortex time in place of having a separate incubation period, but it is not apparent whether this approach affects the extraction efficiency or sensitivity [37,49,52]. Likewise, the impact of ultrasonication is not clear since it was used only in one assay [50]. Centrifugation was a common step in all assays with a wide range of utilized speed and time, with no apparent advantage of any specific approach (Table 1). Drying/sample concentrating was typically conducted with a nitrogen stream or, less commonly, with vacuum evaporation (Table 1).

Both IxS and *p*CS are highly protein-bound [17,19], and it is more toxicologically relevant to quantify the unbound concentrations that are considered biologically active. However, only a few assays have quantified the free concentration (Table 1), likely due to the much longer sample processing time, higher costs, and the requirement for additional ultracentrifugation steps. Ultracentrifugation was the common approach to isolate the free fractions of IxS and *p*CS. As the molecular weights of the bound fraction exceed 60,000 Da [31], the filters commonly had a molecular weight cut-off (MWCO) of 3000–30,000 Da. In Boelaert et al. [40], the Millipore Centrifree device with an MWCO of 30,000 Da was used for ultracentrifugation at 1469× *g* for 25 min. The LLOQ was 75 and 95 ng/mL for IxS and *p*CS, respectively [40]. The same device was employed by Ma et al. [46] with higher centrifugation speed (3500× *g*) and longer time (30 min), with a corresponding LLOQ of 20 and 19 ng/mL for IxS and *p*CS, respectively [46]. In Lin et al. [29], the serum was centrifuged with a Merck Millipore filter with a MWCO of 3000 Da at 10,621× *g* for 30 min to provide an LLOQ of 50 ng/mL for both IxS and *p*CS [29]. On the other hand, in Shu et al. [37], serum samples were centrifuged at 20,000× *g* for 5 min with an unspecified device [37]. The LLOQ was 500 and 50 ng/mL for IxS and *p*CS, respectively [37]. Fabresse et al. [31] utilized the ultra-centrifugal 30,000 Da filter (Amicon, Merck) with centrifugation at 13,300× *g* for 20 min and reported relatively higher sensitivity with an LLOQ of 1 ng/mL for both IxS and *p*CS [31]. The sensitivity of the latter assay may also be attributed to their sample preparation technique (i.e., complete evaporation of the extracted sample and reconstitution with pure solvent (Table 1)) or more sensitive mass spectrometry parameters [31]. Generally, the quantification of unbound IxS and *p*CS does not only require extra sample preparation but needs assays with higher sensitivity as the serum/plasma free fraction for IxS and *p*CS are relatively low at 5–10% of the total concentration [61]. According to the literature, the IxS and *p*CS-free concentrations can be as low as 80 and 150 ng/mL in CKD patients, respectively (e.g., [29]).

#### 3.1.2. Liquid Chromatography

The composition of the mobile phase depends on the instrument hardware, method of ionization, and utilized analytical column. In all assays discussed in this review, an aqueous phase containing additives (discussed below) is utilized (Table 1) due to the water solubility of IxS and *p*CS [62,63]. The organic solvent of choice is either methanol or ACN (Table 1) due to favorable chemical interactions with IxS and *p*CS already discussed above. As there was a lack of a systematic evaluation/comparison of different organic solvents in a single setting, it was not clear why most assays favored methanol or ACN. Likewise, data/descriptions on chromatography (i.e., resolution, background interference, retention factor, etc.) were not always available, which limited a direct comparison between methanol vs. ACN. However, ACN was used as the organic phase in more assays (Table 1), including a few assays that had used methanol in their sample extraction process [31,32,38]. Additionally, ACN was employed as the organic phase in assays with relatively shorter run times (i.e., <6 min) quantifying a mixture of IxS and *p*CS [29,37,39,44,45], compared to the run time (i.e., 6–22 min) achieved by assays utilizing methanol as the organic phase [40,42,43,49,52,53]. This is likely due to the potentially stronger interaction between ACN and IxS or *p*CS, as already discussed above. By comparing the available chromatograms, the majority of assays utilizing methanol as the organic phase exhibited relatively sharp/narrow peaks [40,42,43,53]. However, although most assays utilizing ACN illustrated sharp peaks, some exceptions were identified. For example, background interference and/or some asymmetry were observed in IxS and *p*CS peaks [45,46,51]. Notably, most assays using ACN and methanol, which reported less than ideal chromatographic peaks, were all tailored to analyte mixtures, perhaps due to the complexity of having to separate multiple compounds (e.g., [45,46,49]).

The mobile phase additives included the inorganic ammonium acetate or ammonium formate salts at concentrations between 2–10 mM and formic acid or acetic acid mainly at a concentration of 0.1% *v*/*v* (Table 1). The ammonium salts, acting as buffers, were commonly added to the aqueous phase but not the organic solvents, likely due to solubility (e.g., ammonium acetate is soluble in aqueous media up to ~148 g/100 cc and much less so in methanol ~7.89 g/100 cc [64]). Ammonium salts appear to affect the separation and sensitivity of IxS and *p*CS but not the total run time. Assays utilizing ammonium salts had an IxS-*p*CS separation between 0.58–1.7 min; total run between 4–20 min; IxS LLOQ between 21–500 ng/mL; and *p*CS LLOQ between 50–508 ng/mL (Table 1). Ammonium salt additives were utilized in both isocratic and gradient mobile phase conditions (Table 1). On the other hand, assays without ammonium salts had IxS–*p*CS separation of 0.04–0.47 min, at a total run time of between 2.5 and 22 min, and both IxS and *p*CS LLOQ ranged between 1 and 100 ng/mL. Few assays had chromatograms with asymmetric peaks or background interference (as discussed before). Of these assays, some utilized ammonium salts, such as Oda et al. [51], and some did not use ammonium salts, such as Ma et al. [46]. Therefore, other than the slightly longer separation between IxS and *p*CS, the impacts of ammonium salts on the overall chromatography (i.e., sensitivity, resolution, total run time…etc.) are not apparent. Furthermore, formic acid and acetic acid are added to adjust the pH of the mobile phase but not to all assays [37,43,52]. Assays with formic acid or acetic acid had wide LLOQ ranges between 1 and 405 ng/mL for IxS and 1 and 508 ng/mL for *p*CS. These assays provided an IxS–*p*CS separation of 0.04–1.4 min (Table 1). Similarly, the LLOQ values for assays that lacked acid additives were 21–500 ng/mL for IxS and 50 ng/mL for *p*CS [37,43,52], and the IxS–*p*CS separation was between 0.58–1.7 s. Therefore, the presence of formic or acetic acid does not appear to affect IxS and *p*CS sensitivity and separation (Table 1), which is consistent with the discussion provided by Fernandes et al. [8]. The assays utilizing formic or acidic acid in the mobile phase provided variable chromatogram shapes, where some assays provided asymmetrical peaks with tailing (e.g., [45,46,51]), but others did not show apparent abnormalities (e.g., [38,50]). Similarly, the assays with no formic or acidic acid also provided relatively sharp peaks with little fronting or tailing (e.g., [37,43,52]); therefore, the presence of these acids might not be essential for chromatography.

Isocratic elution was used by Prokopienko et al. [44] (10 mM ammonium formate [85%]: ACN [15%]), Shu et al. [37] (10 mM ammonium acetate [90%]: ACN [10%]), and Ma et al. [46] (acetic acid [0.1%] in water [60%]: ACN [40%]) [37,44,46]. It was common for the analytes to elute at higher aqueous compositions (Table 1). The run times were ~4, 5, and 8 min, respectively, where the IxS LLOQs were 200, 500, and 20 ng/mL, and the *p*CS LLOQs were 250, 50, and 19 ng/mL, respectively (Table 1). The columns and flow rates were BEH C18 (1.7 µm, 2.1 × 100 mm; 0.3 mL/min), SB-C18 (3.5 µm, 2.1 × 100 mm; 0.3 mL/min), and Poroshell 120 HILIC (4.6 µm, 2.7 × 100 mm; 0.6 mL/min), respectively. The use of higher organic compositions in Ma et al. [46] appeared to be associated with lower LLOQs for both IxS and *p*CS (20.14 ng/mL and 19.54 ng/mL, respectively) [46] compared to LLOQs higher than 50.00 ng/mL for both IxS and *p*CS in Shu et al. [37] and Prokopienko et al. [44]; whereas better separation (1.7 and 1.19 s) between IxS and *p*CS was evident at a lower flow rate (0.3 mL/min) in Shu et al. [37] and Prokopienko et al. [44] compared to 0.08 s in the Ma et al. [46] (0.6 mL/min). The higher IxS and *p*CS sensitivities at higher organic composition could be due to the interacting effects with ACN. Furthermore, all isocratic assays exhibited chromatograms with sharp peaks and negligible background interference except for Ma et al. [46] where minor asymmetrical peaks were evident for *p*CS [46], which might be attributed to the use of the HILIC column, which may exhibit different interactions with sulfonated analytes compared to C18 columns [50].

On the other hand, the majority of the assays utilized a gradient composition (Table 1), which typically starts with higher concentrations of the aqueous phase with increasing concentrations of the organic phase and re-equilibrium to baseline conditions over time. When gradients are used, both IxS and *p*CS are mostly eluted at relatively higher concentrations of the organic phase (e.g., ~60–95% [42,43,45]), with the flow rate ranging between 0.2–0.8 mL/min, total run times from 2.5–22 min, LLOQs between 1 and 405 ng/mL and 1 and 508 ng/mL for IxS and *p*CS, respectively, and the separation between IxS and *p*CS from 0.04 to 1.4 min (Table 1). Additionally, the chromatography was not ideal in some assays (e.g., [45,49,51]). As such, although gradient conditions were more commonly used in complex assays for quantifying multiple analytes, there does not appear to be a clear advantage over isocratic conditions for analyzing IxS and *p*CS with respect to chromatography, run time, or sensitivity (Table 1). However, in assays that quantify multiple analytes (in addition to IxS and *p*CS), gradient conditions may be better suited for separating IxS and *p*CS from other uremic toxins. For example, in de Loor et al. [42], IxS and *p*CS eluted at 95% of the organic phase, and five other analytes eluted at 3–16% of the organic phase [42].

Almost half of the analyzed gradient assays used a stable flow rate of ≤0.3 mL/min, with some assays reaching up to 0.8 mL/min (Table 1). On the other hand, two assays used a varying flow rate of up to 0.6 mL/min [49,50] (Table 1) and reported LLOQs of 19 and 405 ng/mL for IxS and 24 and 508 ng/mL for *p*CS. The separation between IxS and *p*CS was 0.14 and 1.15 s, which was achieved in a total run time of 6 and 20 min. Consistently, assays with fixed flow rates reported LLOQs between 1 and 156 ng/mL for IxS and 1 and 100 ng/mL for *p*CS. The separation between IxS and *p*CS in fixed-flow rate assays varied between 0.04–1.4 min achieved in a total run time between 2.5 and 22 min (Table 1). Therefore, it is not clear whether there is any utility for changing the flow rate during the run for analyzing IxS and *p*CS. However, a possible advantage is the separation of multiple analytes. For example, Monosik et al. [49] were able to elute three analytes at a flow rate of 0.55 mL/min, four additional analytes at 0.43 mL/min (including IxS and *p*CS), and the remaining analyte at 0.6 mL/min [49]. Additionally, varying flow rates can potentially shorten the total run time for multiple-analyte assays by controlling the timings of rate changes tailored to analyte elution and column re-equilibration.

The elution of IxS and *p*CS typically utilizes reverse-phase chromatography due to the lipophilic and charged interactions between the analytes and the column. Kanemitsu et al. [50] investigated different types of columns for the quantification of uremic toxins, including IxS and *p*CS [50]. The tested columns included the XSELECT CSH C18 (2.1 mm, 150 mm, 3.5 µm), Luna Phenyl-Hexyl (2 mm, 100 mm, 3 µm), SeQuant ZIC-HILIC (2.1 mm, 50 mm, 3.5 µm), Scherzo SM-C18 (2.1 mm, 100 mm, 3.5 µm), Scherzo SW-C18 (2.1 mm, 100 mm, 3.5 µm), and Scherzo SS-C18 (2.1 mm, 100 mm, 3.5 µm). According to the authors, C18 and Phenyl-Hexyl columns resulted in poor separation for higher polarity toxins such as trimethylamine-N-oxide (TMAO), whereas IxS and *p*CS could only be poorly resolved [50]. The ZIC-HILIC column produced shorter retention times for IxS and *p*CS [50], and the chromatograms showed some tailing was observed in the IxS and *p*CS peaks. Chromatograms generated by Scherzo SW-C18 produced evident tailing in *p*CS peaks, whereas chromatograms from Scherzo SM-C18 had narrower peaks for IxS and *p*CS [50]. According to Kanemitsu et al. [50], the Scherzo SS-C18 column produced better retention for IxS compared to *p*CS, resulting in ideal separation with a distinct order of elution, likely due to the ability of IxS to generate both “hydrophobic and ionic interactions” with this column [50]. Generally, broader but symmetric peaks were associated with Scherzo SS-C18 for IxS and *p*CS.

The particle size ranged between 1.7 and 5 µm (Table 1). Columns with particle sizes of 1.7–2.7 µm provided an IxS–*p*CS separation of 0.08–1.4 min, which was similar to the separation of 0.04–1.7 min from columns with particle sizes between 3 and 5 µm (Table 1). Furthermore, assays utilized 100 mm columns (eight assays) [29,31,37,40,44,46,47,49], 50 mm columns (six assays) [39,42,43,45,50,51], or longer columns (210 mm [38] and 150 mm [32,52,53]). IxS–*p*CS separation from columns ≥100 mm ranged between 0.08 and 1.7 min, which was only slightly longer than the 50 mm columns (0.04–1.15 min). On the other hand, the total run time for columns ≥100 mm ranged between 4–22 min, which was similar to 2.5–20 min associated with the 50 mm columns (Table 1). As mentioned before, asymmetric chromatography was associated with some assays [45,46,49,51] with no apparent correlation with column particle size and length (Table 1).

### 3.2. Analytical Assays in Other Matrices

Although the majority of methods quantifying IxS and/or *p*CS were validated for serum and plasma, assays in urine [32,49,54,55] and saliva [56,57,58] were also available (Table 1).

#### 3.2.1. Sample Preparation

Extraction protocols are required in urine and saliva for sample cleanup (Table 1). For urine, centrifugation may be required for the initial sample processing before the addition of the precipitation solution [49,55], and sample dilution was common in all urine assays in this review (Table 1). The LLOQ for IxS and *p*CS ranged between 1 and 480 ng/mL and 1 and 24 ng/mL, respectively, whereas the ULOQ for IxS and *p*CS ranged between 9.86 and 48 µg/mL and 1 and 12.47 µg/mL, respectively (Table 1). According to the literature, the urine concentrations of IxS and *p*CS in kidney transplant patients were 48.4 ± 52.39 µM (10.32 ± 11.17 µg/mL) and 37.2 ± 65.91 µM (7.00 ± 12.40 µg/mL), respectively (e.g., [65]). Where the serum concentrations of IxS and *p*CS for the same population were 1.9 ± 2.9 µM (0.41 ± 0.62 µg/mL) and 4.1 ± 5.76 µM (0.77 ± 1.08 µg/mL), respectively [65]. Therefore, urine sample dilution may be required due to the significantly higher concentrations of IxS and *p*CS observed in the urine. The correlations between IxS and *p*CS serum concentrations and their respective urine concentrations were statistically significant (*p* < 0.0329) and (*p* < 0.0001), respectively [65]. Although urine sample collection is not invasive, it is not convenient (i.e., necessitating additional privacy) and requires various collection standards (e.g., clean location and appropriate transportation) to avoid sample contamination.

On the other hand, saliva requires additional assay steps for sample collection and preparation. It is important to consider that a collecting tool (e.g., cotton swab) is needed, and either stimulated or unstimulated saliva may be utilized [56,57,58]. Three saliva assays were identified in our review (Table 1), and similar to plasma/serum, both ACN and methanol were used in the extraction process. Nam et al. [58] studied the effects of three extraction solvents (methanol, ACN, and methanol–ACN mixture) and reported ACN to be superior as it resulted in the lowest intensities for large molecules (*m*/*z* > 550), suggesting better removal of proteins [58]. Giebułtowicz et al. [56] incubated saliva samples in ACN for 20 min for deproteinization and reported a comparable matrix effect compared to Fabresse et al. [57], where methanol was used for the extraction without an incubation step. The other sample preparation steps were generally comparable to those described in serum/plasma assays (Table 1).

The LLOQs for saliva assays were 1–1.5 for IxS and 1–3.7 ng/mL for *p*CS (Table 1), with ULOQ reaching 50 µg/mL for both IxS and *p*CS (Table 1). In the literature, the IxS saliva concentrations in kidney transplant patients are 21–33 ng/mL, whereas the *p*CS saliva concentrations are 46–57 ng/mL [66]; therefore, these assays exhibit sufficient sensitivities. The serum IxS and *p*CS concentrations for the same population were 1715–2075 ng/mL and 4697–7103 ng/mL, respectively [66], in which a significant univariate correlation was evident between saliva and serum concentrations of IxS and *p*CS [66]. The limitations associated with saliva samples include (i) contamination, (ii) interactions with food, beverages, and oral bacteria, (iii) multiple factors affecting the salivary flow rate such as the time of collection (e.g., day vs. night), gender, and hydration level, and (iv) interactions with patients’ concurrent medications (e.g., adverse effects such as mouth dryness from anticholinergics) and health conditions (sympathetic and parasympathetic systems) [67,68].

#### 3.2.2. Liquid Chromatography Conditions

In the assays validated by Monosik et al. [49] and Zhu et al. [32], the same liquid chromatography conditions were used for serum/plasma as for urine within the same assay [32,49], indicating no additional specific requirements for urine after sample preparation. On the other hand, the reviewed saliva assays were only validated in this specific matrix. Overall, the chromatographic conditions were generally comparable between assays that have been specifically validated in urine or saliva and in serum or plasma (Table 1) with respect to mobile phase (both ACN and methanol were used) and column, leading to consistent assay performance parameters such as LLOQ (as low as 1 ng/mL for both IxS and *p*CS) (Table 1).

### 3.3. Mass Spectrometry

In addition to the high sensitivity provided by LC-MS/MS, it has significantly better selectivity compared to other analytical techniques (e.g., ultra-performance liquid chromatography). Electrospray negative ionization (ESI) with multiple reaction monitoring (MRM) was the most common method for detecting IxS and *p*CS (Table 1). Atmospheric pressure chemical ionization (APCI) requires the analyte to be volatile, and it was not utilized in the reviewed papers as it is not suitable for polar analytes such as IxS and *p*CS [69]. None of the assays used positive ionization to quantify IxS or *p*CS (Table 1), and this was expected since this approach is more suitable for amino groups, amides, or carbonyls [70], which are not associated with IxS and *p*CS. The reported mass-to-charge (*m*/*z*) transitions were consistent between the assays: IxS from 212 → 80 (the sulfate group) or 212 → 132 (the indoxyl group) and *p*CS from 187 → 80 (the sulfate group) or 187 → 107 (the *p*-cresol). The assays that used ammonium salts also reported comparable MRM transitions, reflecting a low probability for adduct formation (Table 1). Various internal standards were utilized, including hippuric acid–d_5_ [40,46], indole-3-acetic acid–d_5_ [40], tryptophan–d_3_ [53], *p*-toluene sulfonic acid [38,52], and ^13^C_6_ ([^13^C_6_-IxS]) [45] (Table 1), but IxS-d_4_ and *p*CS-d_7_ were most used (Table 1) and provided the ideal internal reference standard due to identical physiochemical characteristics as the original un-deuterated analytes.

Unique mass spectrometry parameters were evident in the reviewed assays (Table 1); however, some parameters were commonly observed (when data were provided). For example, nitrogen was frequently used as the nebulizing gas [31,37,42] and argon as a collision gas [31,39,40,42,49,50,51], with some exceptions where nitrogen was utilized for collision [37,47,58]. Across the assays (Table 1), the source temperature ranged between 105 and 600 °C. The LLOQ range for assays with a source temperature of <350 °C was 19–500 ng/mL for IxS and 1–95 ng/mL for *p*CS. The LLOQ for assays with a source temperature of ≥350 °C were 1–100 ng/mL for both IxS and *p*CS. The ion spray voltage was typically between 1500 and 5000 V. Overall, the assay development data for ionization parameters have generally not been provided in the reviewed papers (Table 1), making it difficult to assess their effects within each assay.

### 3.4. Assay Validation

A variety of guidelines were followed for assay validation, with the Food and Drug Administration (FDA) [71] and European Medicines Agency (EMA) [72], both previous versions, being the most commonly used (Table 1). Although the validation guidelines are generally similar, some differences can be observed; therefore, assays considered validated based on different standards may still exhibit inconsistent performances. For example, back conversion is not required by this version (i.e., year 2018) of the FDA document [71,73], and EMA provides more specific suggestions for the use of isotope-labeled internal standards in mass-spectrometry [72,73]. Furthermore, some validation criteria might not be completely consistent between these two versions of the guidance documents (e.g., matrix effect) [71,72], and indeed, different methods for detecting/reporting matrix interference have been noted in the reviewed papers, which may lead to inconsistencies in data interpretation. Overall, the majority of the reviewed assays have provided accuracy and precision, stability, recovery, and matrix effect, however, some assays did not provide full validation data (Table 1). Table 1 provides summaries of the assay validation results.

### 3.5. Analytical Assay Summary and Future Directions

**Figure 4:**
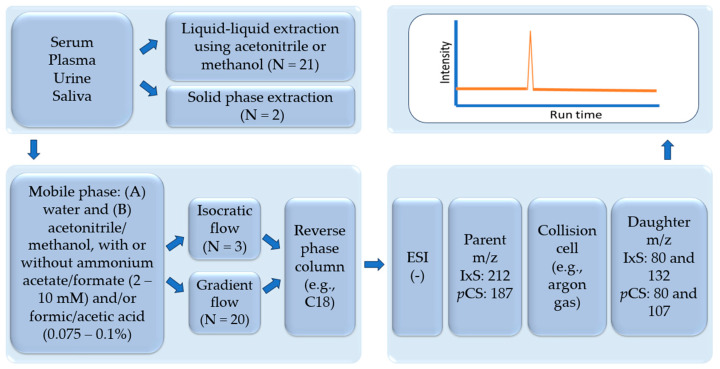
Summary of common assay preparation, chromatography, and mass spectrometry conditions summarized in this review. ESI: electrospray ionization. IxS: indoxyl sulfate. *p*CS: *p*-cresyl sulfate. N: number of LC-MS/MS assays in each category. (–): negative ionization mode.

The review critically summarizes the published LC-MS/MS assays for quantifying IxS and *p*CS in human biological samples, including sample preparation protocols for both unbound and total concentrations (a summary figure of common workflow is presented in Figure 4; see Table 1 and text for specific differences). Liquid–liquid extraction is the most common approach and is typically conducted with methanol or ACN; however, a systematic comparison between a variety of organic extraction solvents is not yet available in the literature and would benefit future assays. The mobile phase of choice in all reviewed biological matrices consisted of water with either methanol or ACN with formic acid, acetic acid, and/or ammonium acetate. According to our analysis, these mobile phase additives generally do not affect the chromatography of IxS and *p*CS. The most commonly used analytical columns had a C18 stationary phase, which produced sufficient separations between IxS and *p*CS. A comparison between the different column parameters, in addition to the coating material, would also benefit future assays trying to analyze these uremic toxins. Gradient and isocratic conditions are both suited for separating IxS and *p*CS, although gradients are more commonly used if multiple toxins are being separated/analyzed. Negative electrospray ionization was used in all assays with consistent MRM transitions for IxS and *p*CS but variable ionization conditions are observed across the studies. Further controlled experiments are also needed to determine the most optimal ion source conditions for ionizing IxS and *p*CS. While the majority of assays have been developed in serum/plasma, analytical assays in saliva and urine are also available. The sample preparation procedures should be tailored to these specific biological samples, but the chromatographic and mass-spectrometry conditions remain comparable across the variety of matrices reviewed.

### 3.6. Clinical Data Obtained Using the IxS and pCS LCMS/MS Assays Validated in Human Biological Matrices

**Figure 5:**
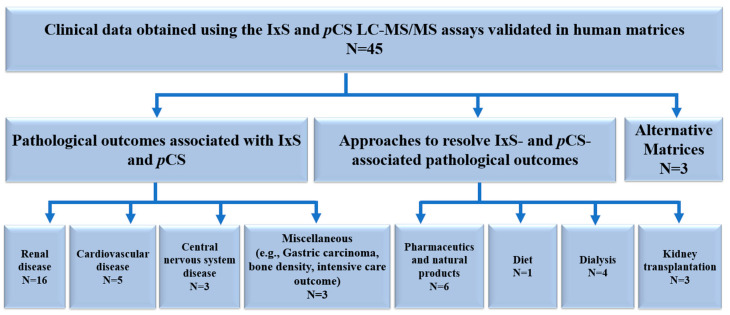
Summary of the biological application data citing the validated LC-MS/MS assays included in the review. IxS: indoxyl sulfate. *p*CS: *p*-cresyl sulfate. LC-MS/MS: liquid chromatography-mass spectrometry. N: number of biological applications in each category.

The accumulations of IxS and *p*CS are thought to be associated with organ and tissue malfunctions. The LC-MS/MS assays included in this review were employed in the quantification of IxS and *p*CS in various indications (Table 2). As discussed in the methods section, only the biological applications using the fully validated LC-MS/MS assays cited in Table 1 were further examined (Table 2). Figure 3 summarizes the biological application literature review process, citing the validated LC-MS/MS assays included in our paper. The data were categorized based on pathological outcomes associated with IxS and *p*CS, approaches used to mitigate these outcomes, and alternative matrices for quantifying IxS and *p*CS (Figure 5).

#### 3.6.1. Liquid Chromatography Conditions

The biological applications were classified according to organ systems (Figure 5, Table 2). The accumulations of IxS and *p*CS were mostly associated with renal insufficiency; however, additional organ pathologies have also been identified (Figure 5).

##### Renal Disease

Several studies have correlated the concentrations of IxS and *p*CS with symptoms of uremic syndrome secondary to CKD progression. IxS and *p*CS total serum concentrations in healthy individuals in the reviewed data were 0.25–3.02 µg/mL and 0.29–9.87 µg/mL, respectively (Table 2). IxS and *p*CS serum concentrations increase proportionally with CKD progression. This is evident in Boelaert et al. [40], who reported the progressive accumulations of IxS and *p*CS serum concentrations in pre-dialysis patients in CKD stages 1 to 5. Significant elevations were observed at stage 3 (IxS: 1.7 ± 1.1 µg/mL and *p*CS: 20.0 ± 7.3 µg/mL), reaching up to 4.6 ± 2.1 µg/mL and 26.5 ± 9.8 µg/mL, respectively, at stage 5 for patients not on dialysis [40]. These represent significant elevations compared to IxS (0.5 ± 0.3 µg/mL) and *p*CS (6.6 ± 3.7 µg/mL) serum concentrations in healthy controls within the same study [40]. Accumulations of IxS and *p*CS are also observed in serum and plasma samples at CKD stages 4 and 5 by Massy et al. [96] and Lin et al. [29] and at stages 3–5 in Oda et al. [51]. Although de Loor et al. [42] and Cuoghi et al. [38] (Table 2) did not provide detailed information pertaining to each CKD stage, the average data for pre-dialysis patients with CKD illustrated that IxS and *p*CS concentrations were generally elevated compared to healthy individual data in the literature.

Patients on dialysis were also reported to have accumulated concentrations of IxS and *p*CS. IxS and *p*CS serum/plasma concentrations in patients on hemodialysis were reported to be 3.5 ± 1.5 µg/mL to 23.74 µg/mL and 16.68 ± 8.8 µg/mL to 55.9 ± 15.1 µg/mL, respectively (Table 2). In patients on peritoneal dialysis, IxS and *p*CS serum/plasma concentrations were 17.6 ± 8.7 µg/mL to 32.33 ± 20.18 µg/mL and 4.6 ± 2.7 µg/mL to 23.90 ± 19.87 µg/mL, respectively (Table 2). Concentrations of IxS and *p*CS in hemodialysis patients were significantly higher than that reported in healthy volunteers and patients with chronic kidney disease (e.g., [40,51,82]) (more discussion regarding dialysis is available in Section Dialysis). Consistently, significant inverse correlations were observed in a CKD population between serum IxS or *p*CS concentrations and eGFR in de Loor et al. [42]. Similar data were reported for serum or salivary IxS or *p*CS and eGFR by Korytowska et al. [66] and serum IxS and eGFR by Wang et al. [85]. However, Shu et al. [37] only reported significant correlations between serum-free and total IxS, but not *p*CS, with eGFR.

The accumulation of IxS and *p*CS concentrations in different CKD stages is typically observed before the complete loss of kidney function, which suggests that monitoring IxS and *p*CS concentrations might be a potential approach for the detection of CKD progression. According to Lin et al. [29], IxS and *p*CS concentrations can be a predictable tool for CKD development and can be used as a therapeutic treatment for delaying the need for dialysis [29]. Furthermore, creatinine-based GFR calculations may not be the best marker for detecting early stages of CKD, and it may result in the overestimation of renal function, for example, in patients with liver diseases [99]. In addition, these sulfonated PBUTs could act as diagnostic markers for acute kidney injury (AKI) as suggested by André et al. [45], as the authors reported IxS and *p*CS serum concentrations to be elevated significantly in 8 patients who developed AKI within 72 h of acute care admission [45]. Therefore, IxS and *p*CS may potentially serve as better biomarkers for measuring renal function in patients with renal disease, but this would require extensive clinical and outcome validation in the clinic.

Various potential mechanisms by which IxS and *p*CS may lead to the worsening of CKD have been documented in the literature. As examples, IxS and *p*CS could generate oxidative stress and/or enhance free radical production, as evident by in vitro and in vivo experiments [100,101,102,103]. Mouse proximal renal tubular cells treated with IxS and *p*CS express genes related to inflammation response, which may be a compensatory response to oxidative stress injury [104]. Moreover, 5/6-nephrectomized rats administered IxS exhibit elevated mRNA of tubulointerstitial fibrosis-related genes [105], and IxS is also capable of generating the production of free radicals in rodent mesangial cells [106]. Additional pathological mechanisms include IxS and *p*CS activating both the renal renin-angiotensin-aldosterone system and the transforming growth factor β1 pathway, leading to an injured kidney [107]. Interactions with cellular transporters were also evident in Mutsaers et al. [108] in proximal renal tubular epithelial cells, where *p*CS was shown to inhibit multidrug resistance protein 4 and the breast cancer resistance protein [108]. These transporters are responsible for the renal uptake and therefore the excretion of drugs, metabolites, and endogenous molecules [108].

On the other hand, the impairment in kidney function can also lead to the accumulation of IxS and *p*CS, which may create a pathological feedback loop, resulting in further oxidative stress and the eventual worsening of kidney function. As IxS and *p*CS accumulations are significant around CKD stage 3 [40], this would indicate that earlier stages of CKD (i.e., initiation of disease state) were accompanied by normal serum levels of IxS and *p*CS, supporting the notion that renal dysfunction might possibly precede the accumulation of these PBUTs.

##### Cardiovascular Disease

IxS and *p*CS accumulation in CKD is potentially associated with the progression or development of cardiovascular disease (CVD), as evident by, for example, lipid profile [75], arterial stiffness [85], thrombosis [86], cardiovascular autonomic function [90], and fat deposits in skeletal muscle [83] (Table 2). Hobson et al. [75] reported that IxS and *p*CS were negatively correlated (univariately) with total cholesterol, low-density lipoprotein (LDL), and non-HDL in 611 patients with kidney failure [75] (Table 2). Wang et al. [85] demonstrated that IxS was an independent predictor of arterial stiffness in CKD patients using multiple regression, where categorical analysis also indicated CKD patients exhibiting arterial stiffness had significantly higher IxS concentrations [85]. Furthermore, the accumulation of IxS in CKD patients was associated with thrombosis, indicated by greater activities of aryl-hydrocarbon-receptor (AHR) and tissue factor (TF) as demonstrated by Kolachalama et al. [86] (Table 2). Cheng et al. [90] reported that significant reductions in IxS concentrations after peritoneal dialysis were associated with improvements in sympathetic/parasympathetic imbalance in 26 patients [90]. Keddar et al. [83] reported that a significant reduction in muscle radiation attenuation was associated with cardiovascular events in CKD that are also linked with the accumulation of *p*CS [83]. In this study, muscle radiation attenuation is an indicator of the amount of fat deposits in cardiac muscles, and higher values indicate lower fat deposits [83]. Moreover, the development of kidney failure was associated with lower muscle radiation attenuation, and kidney transplantation was able to elevate the muscle radiation attenuation [83]. Therefore, the relationship between muscle radiation attenuation and *p*CS concentrations may be mediated by renal function. However, the cause-effect relationships between these variables still remain to be established.

Potential mechanisms have been identified for the effects of IxS and *p*CS on cardiovascular outcomes in CKD patients. IxS is proposed to increase the uptake of oxidized LDL in the THP-1 macrophages, which facilitates the development of atherosclerosis [109]. IxS was reported to induce the proliferation of rat vascular smooth muscle cells and human aortic smooth muscle cells [110,111], potentially leading to vascular dysfunction. Consistent evidence is observed in rats where IxS contributes to the aortic wall thickness and aortic calcification [112], and *p*CS leads to vascular remodeling with oxidative stress induction, potentially leading to the development of cardiovascular events [113].

##### Central Nervous System Disease

CKD is also associated with nervous system complications [114]. For example, a study with 225 CKD patients on dialysis revealed that 16.4% of subjects experienced uremic neuropathy [115]. Table 2 included three studies reporting associations between IxS and *p*CS and central nervous system (CNS) complications in CKD patients. Lai et al. [87] reported that free IxS was independently associated with the composite amplitude score, which determines the severity of peripheral nerve dysfunctions in 54 stages 3–4 CKD patients (50% with diabetes; data not available with *p*CS), although no significant differences in IxS and *p*CS concentrations were reported in patients with diabetes compared to patients without diabetes [87] (Table 2). IxS and *p*CS can cause oxidative stress [100,101,102,103], and according to Lai et al. [87], IxS may exert similar pathophysiological effects resulting in neuropathy [87]. Furthermore, Olesova et al. [55] quantified urinary IxS and *p*CS (and other PBUTs) and reported significantly higher IxS concentrations in children 6–10 years old with autism spectrum disorder (ASD) compared to age-matched controls [55] (Table 2). According to the authors, a potential explanation might be that ASD is associated with changes in the microbiota and, therefore, may affect IxS production and concentrations [55]. However, further investigations and clinical studies are required before IxS can be considered as a diagnostic biomarker for ASD. On the other hand, te Linde et al. reported significant reductions in serum uremic toxins, including (IxS and *p*CS) with renal transplantation, but the improvement in cognitive function was not observed [76]. Therefore, further mechanistic experiments are warranted to determine how IxS and *p*CS affect cognitive functions in patients and whether the effects are clinically relevant.

##### Miscellaneous

CKD also impacts other organ systems or conditions. Table 2 summarizes the studies relating IxS and *p*CS concentrations to the gut microbiota, bone density, and ICU outcomes. Choi et al. [53] investigated the use of tryptophan metabolites as potential biomarkers for the development of gastric carcinoma [53] and reported plasma IxS to be significantly lower in gastric cancer patients [53]. However, the mechanisms and the cause-effect relationships between IxS and gastric cancer development remain to be further proven. Batteux et al. [94] characterized osteoporosis as measured by bone mineral density (BMD) and by bone fractures after transplantation [94] (Table 2). It was evident that serum IxS concentrations at the time of transplantation exhibited a positive relationship with BMD in the femoral neck and the total hip at one-month post-transplant [94], but *p*CS did not generate significant correlations. On the other hand, there were no associations between uremic toxins measured at the time of transplantation and BMD or bone fracture after up to 24 months post-transplantation [94]. According to the authors, the reduction in BMD after transplantation could be a result of multiple factors other than uremia, including changes in the body mass index, serum parathyroid hormone, and calcium concentrations in serum [94]. Kuo et al. [97] investigated the relationship between urine IxS concentrations and outcomes in the intensive care unit (ICU) [97]. It was evident that ICU patients who had lower urine IxS concentrations after 72 h of ICU admission were associated with reduced ICU-free days and higher mortality at one year [97]. However, the underlying mechanisms behind this association and whether serum concentrations held the same relationship remain unknown. Also, additional studies are needed to rule out all potential confounding effects that may be present in the ICU population.

#### 3.6.2. Approaches to Mitigate IxS and *p*CS Associated Pathological Outcomes

Table 2 summarizes potential approaches to reduce the exposure of IxS and *p*CS, including pharmaceuticals and natural products, diet, dialysis, and kidney transplantation.

##### Pharmaceutics and Natural Products

Biruete et al. [77] investigated the effects of the prebiotic inulin for 4 weeks on gut microbiota composition and gut-derived metabolites in 12 CKD patients on hemodialysis in a cross-over study with maltodextrin as control [77] (Table 2). No significant effects on fecal *p*-cresol and indole or serum IxS and *p*CS were identified with inulin administration compared to maltodextrin [77]. However, both the experimental and the control interventions resulted in distinct patterns of microbiome changes [77]; therefore, maltodextrin may not be an appropriate control, as suggested by the authors [77]. Nazzal et al. [79] reported that the administration of oral vancomycin reduced the concentrations of IxS and *p*CS within 4 weeks of treatment [79]. According to the authors, this may be attributed to vancomycin modulating the microbiome, affecting the formation of IxS and *p*CS [79]. For example, they reported reductions in *Clostridium* and *Bacteroides* which could be responsible for the production of indole that is further metabolized to IxS [79]. However, the effects would have to be reproduced in a larger study with a balanced control group, as the authors indicated potential carryover effects of vancomycin, which precluded the use of half of their control samples [79].

Additional pharmacological approaches to mitigate IxS and *p*CS accumulation have been suggested (Table 2), including the use of adsorbents such as AST-120, which aim to adsorb the amino acids responsible for the final production of IxS and *p*CS [116]. Hsu et al. [91] investigated the effects of AST-120 in CKD patients at stages 4 and 5 [91] but did not observe significant changes in IxS and *p*CS concentrations despite trends of reducing *p*CS [91]. The literature data also provided inconsistent results for AST-120. For example, Schulman et al. reported AST-120 delayed the progression of composite CKD endpoints, including the need for dialysis, transplantation, and doubling of serum creatinine [117], but the study did not appear to have included uremic toxin concentrations. On the other hand, Cha et al. [118] reported that the administration of AST-120 did not delay the manifestation of CKD primary endpoints in patients receiving standard care [118] and that AST-120 did not affect serum IxS concentrations (despite a trend towards a reduction). Similarly, Akizawa et al. [119] also reported no effects of AST-120 on the progression of CKD [119] during 12 months of follow-up, but the study also did not measure uremic toxin concentrations. The potential inconsistent effects of AST-120 are very well-documented in the review article written by Asai et al. [120].

The Shen-Shuai-Ning tea was tested for its effects in reducing uremic toxins in CKD patients on peritoneal dialysis [84], where a significant decrease in serum total IxS, but not *p*CS, concentrations were reported [84]. The divergent effects on total vs. free concentrations may support distinct pharmacokinetic interacting mechanisms such as intrinsic clearance or binding displacement, but further investigations are needed to support these effects. According to the authors, this tea potentially increases bowel movement, which enhances the excretion of toxins [84], but limited data are available in the literature on its effects on IxS and *p*CS concentrations. On the other hand, Dai et al. [80] reported that sevelamer therapy in patients with ESRD was associated with significantly higher concentrations of IxS but not *p*CS [80]. The unexpected relationship with sevelamer, which is typically used to reduce phosphate concentrations in CKD patients, was proposed by the authors to be caused by interactions with the gut environment metabolism and transit times [80]. Biruete et al. [121] also reported in their review the inconsistent effects of sevelamer on IxS and *p*CS [121], indicating more studies are required to establish its efficacy on uremic toxin reduction. El Chamieh et al. [95] investigated the associations between proton pump inhibitors and uremic toxin concentrations in CKD patients [95]. Higher concentrations of total and free IxS and *p*CS were observed in patients receiving proton pump inhibitors, but a significant association with IxS was only observed using multiple linear regression [95]. According to the authors, proton pump inhibitors exhibit high affinities for OAT transporters, which are also responsible for the excretion of IxS and *p*CS, thus potentially explaining this observed interaction [95]. However, the observation was only correlational in nature, and baseline differences between the proton pump inhibitor groups vs. the comparator group were evident, which could potentially confound the findings.

##### Diet

Diet is also an approach to control serum exposure to IxS and *p*CS. In Table 2, Wu et al. [88] investigated the effects of low protein diet on gut microbiota and the generation of IxS and *p*CS in the CKD population [88]. Although significant changes were reported in gut microbiome composition between the experimental and control groups, the study did not demonstrate a difference in serum IxS and *p*CS concentrations in the low-protein diet group [88]. According to the authors, the small sample size can be a reason for the negative effects [88], but the general lack of difference in uremic toxin concentrations between CKD and health controls may also potentially mask their findings. In the literature, Yang et al. [122] suggested in their review that the intake of dietary fiber from vegetable and fruit sources could reduce IxS formation [122]. Takkavatakarn et al. [123] also discussed strategies to reduce IxS and *p*CS, including the use of a (very) low-protein diet [123], but reported inconsistent findings for IxS.

##### Dialysis

Dialysis is the artificial mechanism for removing toxins from the biological system; however, PBUTs are not effectively cleared with conventional dialysis. Ma et al. [46] reported the efficacy of hemodialysis in eliminating IxS and *p*CS to be 24.00 ± 18.29% and 34.28 ± 24.38%, respectively [46] (Table 2). According to Ma et al., the ineffective hemodialysis removal of these PBUTs is due to their high protein binding ratio and high affinity toward serum albumin [46]. Consistently, Ragi et al. [52] illustrated that IxS and *p*CS plasma concentrations pre- and post-hemodialysis were 3.5 ± 1.5 µg/mL to 2.34 ± 0.6 µg/mL and 16.68 ± 8.8 µg/mL to 12.59 ± 7.1 µg/mL, respectively [52]. Etinger et al. [81] hypothesized that dialysate pH affects the clearance of PBUTs and tested two conditions: isohydric dialysis with reduced bicarbonate buffer concentration for the first 90 min vs. standard dialysis with normal dialysate bicarbonate concentration for the full dialysis run [81] (Table 2). The overall reduction in IxS and *p*CS did not differ between the two conditions [81], but this could potentially be attributed to the lack of difference in IxS/*p*CS serum protein binding observed in the two dialysis methods. Furthermore, Vandenbosch et al. [78] tested the effects of asymmetric cellulose triacetate dialyzer with or without anticoagulants for clotting propensity [78]. Although they were not targeting the effects on IxS and *p*CS clearance, they reported no significant differences in the reduction ratios of these UTs without or without anticoagulants [78]. Furthermore, peritoneal dialysis was utilized by only a few studies in Table 2. Cheng et al. [90] demonstrated that IxS and *p*CS serum concentrations were reduced with peritoneal dialysis from 17.6 ± 8.7 µg/mL to 11.7 ± 5.3 µg/mL and from 4.6 ± 2.7 µg/mL to 3.7 ± 1.9 µg/mL, respectively, between baseline to 6-months into dialysis [90], suggesting that PD is also not efficient in removing both toxins. It is difficult to compare the efficiencies of IxS and *p*CS removal between hemodialysis and peritoneal dialysis due to the limited data.

##### Kidney Transplantation

The inefficient IxS and *p*CS removal by dialysis is likely attributed to their high percentage of protein binding; therefore, interventions such as organ transplantation might be required in patients with end-stage renal disease. Kidney transplantation was discussed in these studies in Table 2. te Linde et al. [76] reported significant decreases in serum IxS and *p*CS concentrations after adult kidney transplantation (as mentioned in the Section Central Nervous System Disease) [76] from 24.66 µg/mL to 0.86 µg/mL and 38.32 µg/mL to 5.45 µg/mL, respectively [76]. Korytowska et al. [66] documented salivary and serum IxS and *p*CS concentrations at baseline, ~6 months, and ~12 months after transplant and reported lower concentrations (although not statistically significant) at 6 and 12 months post-transplant compared to baseline [66]. Additionally, Korytowska et al. [66] investigated the correlations between saliva IxS or *p*CS and the deterioration of graft function in kidney transplant patients [66] and only found significant associations with IxS [66].

Furthermore, it is also important to consider the interacting effects of pharmacological agents that are usually administered to kidney transplant patients. André et al. [92] reported an association between higher serum *p*CS, but not IxS, concentrations and the usage of at least one OAT1/OAT3 inhibitor in adult kidney transplant patients [92]. The OAT1/OAT3 inhibitors used in this study included furosemide, acetylsalicylate, and pantoprazole [92], and these drugs likely exhibited different potency and mechanisms of interaction leading to the divergent findings observed with IxS and *p*CS. In addition, tacrolimus concentration-to-dose ratio, but not cyclosporine, was associated with significantly higher IxS and *p*CS serum concentrations in adult kidney transplant patients [93], but multiple regression, including the age variable, reduced the significance of *p*CS. The difference in the associations between the two calcineurin inhibitors might be attributed, according to the authors, to differences in their plasma protein binding properties [93]; however, additional pharmacokinetic interacting mechanisms may also be possible. From the literature, Rong et al. [124] reported a positive correlation between plasma *p*CS and mycophenolic acid trough concentrations [124], but this study did not find an association with tacrolimus. More studies are needed to characterize the interactions between immunosuppressants and IxS or *p*CS, which can potentially cause clinically significant pharmacodynamic effects in this already fragile population [124,125,126].

##### Alternative Matrices

Additional biological matrices, such as urine and saliva, have been utilized for the quantification of IxS and *p*CS (Table 2). Urinary IxS concentrations were correlated with ICU outcomes [97] and autism spectrum disorder in children [55], whereas Wijeyesekera et al. [54] established the reference ranges for urinary *p*CS concentrations in women and men and in two separate age groups (40–49 and 50–59 years old) [54]. These studies did not investigate whether urine was suitable for replacing serum/plasma for quantifying IxS and *p*CS. On the other hand, Giebułtowicz et al. [56] developed an analytical assay for characterizing IxS and *p*CS saliva concentrations in healthy volunteers at ages <30, 30–60, and >60 years (Table 2) [56], and this method was successfully applied to other studies in patients as described below. Additionally, Fabresse et al. [57] quantified the saliva IxS and *p*CS concentrations in healthy volunteers and reported Spearman’s correlations vs. free serum to be 0.78 (*p* < 0.0001) and 0.68 (*p* = 0.002), respectively [57]. Korytowska et al. [98] quantified saliva IxS and *p*CS in CKD patients and also reported significant correlations with free or total serum IxS and *p*CS concentrations (*p* < 0.00001) (Table 2) [98]. Consistent associations between salivary and serum concentrations were also observed in Korytowska et al. [66] in adult kidney transplant patients, where correlation coefficients were reported to be 0.81 (*p* < 0.00001) and 0.92 (*p* < 0.00001) for IxS and *p*CS, respectively [66]. However, despite these significant associations, additional characterizations on the precision and accuracy of saliva for predicting IxS or *p*CS concentrations in the serum are warranted (e.g., [127,128,129]). Although the use of saliva as an alternative matrix for measuring IxS and *p*CS concentrations may be promising, the matrix might be limited by potential contaminations, interactions with food and beverages, and physiological factors that may affect salivary flow (as discussed in Section 3.2.1).

### 3.7. Biological Applications Summary and Future Directions

The available clinical data illustrate the potential impacts of IxS and *p*CS on multiple organ systems. IxS and *p*CS are reported to accumulate with CKD progression, and the accumulation appears to become significant at CKD stage 3 and further elevated in patients on dialysis. These data suggest that CKD may lead to the accumulation of IxS and *p*CS; however, due to their propensity to cause tissue injury, IxS and *p*CS may also result in the progression and, hence, may serve as good biomarkers of CKD. Furthermore, IxS and/or *p*CS are potential predictors of cardiovascular events, as evidenced by associations with arterial stiffness, altered lipid profiles, thrombosis, and fatty tissue deposits. IxS is also associated with peripheral nerve dysfunction and the presence of ASD in children 6–10 years old. On the other hand, although kidney transplantation reduced IxS and *p*CS concentrations, no improvements in cognitive functions were observed. Likewise, several unexpected associations between IxS with gastric cancer, bone mineral density, or ICU outcomes are also observed, which would warrant further investigations. However, the majority of these data were obtained from correlational studies and should be further confirmed with cause-effect investigations.

Several therapeutic methods may be capable of reducing IxS and/or *p*CS concentrations, including drugs such as oral vancomycin, natural products such as Shen-Shuai-Ning, and kidney transplantation, although these are based on a limited amount of data. On the other hand, inconsistent or unexpected effects of AST-120, sevelamer, and diet on IxS and/or *p*CS concentrations are reported, and dialysis appears to be inefficient in reducing IxS and *p*CS. The data also suggest that there is potential for using saliva to monitor IxS and *p*CS concentrations, but further validation using prediction-error analyses is required. Overall, different outcomes/associations with IxS and *p*CS have been observed in various studies in different patient populations (Table 2), suggesting that each toxin may act by distinct interacting/toxicological/pathological mechanisms. As such, mitigation strategies to reduce toxin concentrations may also have to be tailored to each specific sulfonated uremic toxin and to the specific patient population. Further mechanistic investigations of the pathological mechanisms of IxS and *p*CS are also warranted. Finally, as a reflection of the significant interests in this review topic, we have identified further examples of biological applications (e.g., [130,131]) since stopping our systematic literature search (i.e., in the period from December 2023–May 2024). As this is a rapidly evolving field, we recommend revisiting the topic regularly, using this review as a foundation for further discussions.

## 4. Conclusions

This comprehensive review critically evaluated the LC-MS/MS assays that quantified IxS and/or *p*CS in different human biological matrices (Table 1) and presented the clinical applications using these validated assays (Table 2). The majority of the assays focused on human serum/plasma, utilizing acetonitrile or methanol, liquid–liquid extraction, reverse phase (e.g., C18) chromatography, and gradient elution for analyte separation. Mass spectrometry conditions were also consistent in the identified papers, with negative electrospray ionization, select MRM transitions and deuterated internal standards being the most common approaches. The available biological data illustrated the impacts of IxS and *p*CS on both renal and cardiovascular systems, whereas limited data were available on the other organ systems (e.g., CNS, bone density, gastrointestinal health, etc.). These data also supported that IxS and *p*CS might exhibit different toxicity mechanisms and different mitigation strategies might be warranted to detoxify these compounds. The clinical monitoring of IxS and *p*CS is gaining significant interest, and this review will serve as a useful compendium for both the scientific and clinical communities.

## Figures and Tables

**Table 1:** Summary of validated LC-MS/MS assays for quantifying indoxyl sulfate and *p*-cresol sulfate in human biological matrices.

Analyte(s)	Liquid Chromatography Conditions	Mass Spectrometry Conditions	Validation Data	Citation
SERUM				
**Indoxyl sulfate (IxS)** ***p*-Cresol sulfate (*p*CS)** In addition to other analytes: 3-carboxy-4-methyl-5-propyl-2-furan propionate (CMPF), hippuric acid (HA), Indole acetic acid (IAA), *p*-Cresol glucuronide (*p*CG), Uric acid (UA)	**Matrix:** Human serum **Internal standard(s):** Isotope-labeled hippuric acid -d5 (HA-d5, 7.5 µg/mL): IxS Isotope-labeled indole-3-acetic acid-d5 (IAA-d5, 2.5 µg/mL): *p*CS **Column:** reversed phase Acquity UPLC BEH C18 column (1.7 μm, 100 mm × 2.1 mm) Acquity UPLC BEH C18 VanGuard pre-column (1.7 μm, 2.1 mm × 5 mm) **Temperature(s):** Column: 35 °C Autosampler: 8 °C **Mobile phase:** A: 0.1% acetic acid in methanol B: 0.1% acetic acid in 5 mM ammonium acetate (pH 4.3) **Gradient:** 0.0–2.0 min: 95 → 80% B 2.0–5.0 min: 80% B 5.0–6.5 min: 80 → 0% B 6.5–9.0 min: 0% B 9.0–15.0 min: re-equilibration **Injection volume:** 20 µL **Flow rate:** 0.2 mL/min **Retention times:** IxS: 5.7 min d5-HA: 4.4 min *p*CS: 7.1 min d5-IAA: 7.6 min **Total run time:** 15 min (column washed with isopropanol every 15 runs) **Study authors’ sample extraction procedure:** 1. Centrifuge the blood samples, collect the serum, and store at −80 °C; 2. Thaw at room temperature and vortex; 3. Add 40 µL of the internal standard solution to 160 µL serum; 4. Vortex, then add 600 µL water; 5. Expose to 90 °C for 30 min to deproteinate samples; 6. Cool for 10 min on ice; 7. Using a Millipore Centrifree ultrafiltration device (molecular weight cut-off (MWCO): 30,000 Da), ultrafilter samples at 1469× *g* for 25 min; 8. Evaporate 600 μL of ultrafiltrate under nitrogen (N_2_) at room temperature; 9. Use 120 μL of the initial mobile phase (5 mM ammonium acetate buffer solution at pH 4.3 with 5% methanol with 0.1% acetic acid) to reconstitute.	**Mass spectrometer: triple-quadrupole (Waters)** **Mode of ionization:** negative mode **Ion source conditions:** Electrospray ionization (ESI) Drying gas: N_2_ De-solvation gas flow: 650 L/h Cone gas flow: 10 L/h De-solvation temperature: 350 °C Source temperature: 110 °C Capillary potential: 2870 V Collision gas: Argon (Ar) at 2.60 × 10^−3^ Torr **Scanning mode:** Multiple reaction monitoring (MRM) ***m*/*z* transitions:** IxS: 212 → 80 Collision energy = 19 eV Cone voltage = 25 V d5-HA: 183 → 139 Collision energy = 15 eV Cone voltage = 20 V *p*CS: 187 → 107 Collision energy = 18 eV Cone voltage = 25 V d5-IAA: 179 → 135 Collision energy = 10 eV Cone voltage = 15 V	**Validation criteria guideline(s):** EPA ^(a)^ **LLOQ (μg/mL)**: *p*CS: 0.095 IxS: 0.075 **LLOD (μg/mL)**: *p*CS: 0.029 IxS: 0.023 **Low, medium, and high-quality control (QC) (μg/mL):** IxS: 0.25, 7.5, 15 *p*CS: 7.5, 15, 45 **Calibration range(s):** Linearity range (μg/mL): IxS: 0.1–40 *p*CS: 0.1–80 Linearity: R^2^ ≥ 0.99 for all analytes **Selectivity:** No issues **Precision (within-day and between-day) and accuracy** met the acceptance criteria ^(a)^. Accuracy %: <15% IxS; −4.89, −1.33, 0.31 (for low, mid, high QC) *p*CS; 14.00, −1.40, −8.35 (for low, mid, high QC) Within-day precision %: <15% IxS; 4.05, 3.68, 4.00 (for low, mid, high QC) *p*CS; 1.10, 0.60, 1.05 (for low, mid, high QC) Between-day precision %: <15% IxS; 9.15, 8.41, 8.50 (for low, mid, high QC) *p*CS; 7.56, 7.02, 4.69 (for low, mid, high QC) **Stability:** ^(b)^ **Matrix effect (%):** comparison between peak areas resulting from water samples and other samples in serum at the low QC concentration. IxS: −4.37 ± 2.81 (mean ± sample standard deviation (SD); N = 6) *p*CS: −2.52 ± 11.12 (mean ± SD; N = 6) **Recovery (at medium QC concentration; “similar for the low and high QC levels”):** comparison between serum samples spiked before extraction and serum spiked after ultrafiltration. IxS: 91.5 ± 3.9% *p*CS: 98.5 ± 6.3% **Storage conditions:** Stock solutions: −20 °C	Boelaert et al. 2013 [40]
**IxS** ***p*CS** In addition to other analytes: CMPF, HA, IAA, kynurenic acid (KA), kynurenine (Kn), *p*CG, phenyl glucuronide (PG), phenylacetylglutamine (PAG), phenylalanine (Phe), phenyl sulfate (PS) Trimethylamine N-oxide (TMAO), tryptophan (Trp), tyrosine (Tyr)	**Matrix:** Human serum **Internal standard(s):** Isotope-labeled (IxS-d4), original stock 40 μM, and *p*-cresol sulfate-d7 (*p*CS-d7), original stock 20 μM. **Column:** Acquity CSH Fluoro Phenyl column (50 × 2.5 mm; 1.7 µm particle size (Waters, Zellik, Belgium). Precolumn: Acquity CSH Fluoro Phenyl VanGuard (10 × 2.5 mm; 1.7 µm particle size (Waters, Zellik, Belgium). **Temperature(s):** Column: 40 °C Autosampler: 10 °C **Mobile phase**: A: 0.1% formic acid in MQwater B: methanol **Gradient:** 0.00–1.00 min: 3 → 16% B 1.00–4.00 min: 16 → 80% B 4.00–4.50 min: 80 → 95% B 4.50–5.50 min: 95% B 5.50–8.00 min: 3% B **Injection volume:** 5 µL **Flow rate:** 0.5 mL/min **Retention time:** IxS: 4.73 min IxS-d4: 4.72 min *p*CS: 4.83 min *p*CS-d7: 4.82 min **Total run time:** 8 min **Study authors’ sample extraction procedure:** Serum sample preparation: 1. Collect blood samples in vacutainer serum tubes; 2. Centrifuge at 1860× *g* at room temperature for 10 min; 3. Store at –80 °C. LCMS sample preparation: 1. In an Ostro 96-well plate, mix 50 µL serum with 50 µL solution of MQwater/methanol/0.01 N sodium hydroxide (*v*/*v*% 75/20/5); 2. Add 20 µL internal standard solution (20 µM *p*CS-d7, 40 µM IxS-d4, and other deuterated analytes) dissolved in MQwater/methanol, 50/50) and 150 µL acetonitrile (ACN); 3. Mix using an automated pipette (3 times); 4. Apply a positive pressure manifold to separate the precipitate and the supernatant; 5. Collect the supernatant in a 96-well plate (2 mL); 6. Dry precipitate for 30 min at 40 °C using N_2_; 7. Add 1000 µL MQwater to each sample; 8. Seal 96-well plates with a pre-slit cap mat; 9. Place the plates directly into the precooled autosampler (10 °C).	**Mass spectrometer: triple-quadrupole (Waters)** **Mode of ionization:** negative mode **Ion source conditions:** ESI Nebulizing and de-solution gas: N_2_ Collision gas: Ar Source temperature: 150 °C De-solvation temperature: 600 °C De-solvation gas flow: 800 L/h Cone gas flow: 150 L/h Capillary voltage: 3000 V **Scanning mode:** MRM ***m*/*z* transitions** IxS 212.1 → 80.1 Dwell time = 0.02 s Collision energy: 18 V Cone voltage: 35 V IxS-d4 216.1 → 80.2 Dwell time = 0.02 s Collision energy d4-IxS: 16 V Cone voltage: 35 V *p*CS 187.1 → 107.0 Dwell time = 0.02 s Collision energy *p*CS: 20 V Cone voltage: 35 V *p*CS-d7 194.2 → 114.1 Dwell time = 0.02 s Collision energy d7-*p*CS: 20 V Cone voltage: 35 V	**Validation criteria guideline(s):** LLOD according to Armbruster et al. 1994 [41]. Precision (as a coefficient of variation percentage (% CV)) according to NCCLS EP5-T ^(c)^. Serum samples were obtained from patients prior to HD, with low, medium, and high concentrations. Samples were divided into 20 aliquots and analyzed duplicates on separate days. Accuracy: Two added calibrator concentrations in serum samples from healthy volunteers (N = 4) and patients on hemodialysis (HD) (N = 4). **LLOQ:** IxS: 0.2 µM = 0.043 μg/mL *p*CS: 0.3 µM = 0.056 μg/mL **LLOD:** IxS: 0.03 µM= 0.0064 μg/mL *p*CS: 0.03 µM= 0.0056 μg/mL **Calibration range(s):** - Provided in the original manuscript in µM: IxS: 0.2–349 *p*CS: 0.3–586 - Equivalent in μg/mL to the following: IxS: 0.043–74.41 *p*CS: 0.056–110.29 **Precision %** (serum samples from three patients with low, medium, and high toxin concentrations): <15% for all analytes Total precision %: IxS: 4 ± 12, 4 ± 8, 4 ± 0.6 *p*CS: 4 ± 9, 4 ± 3, 3 ± 6 Within-run precision %: IxS; 2 ± 5, 2 ± 4, 2 ± 1 *p*CS; 1 ± 3, 2 ± 1, 1 ± 3 Between-run precision %: IxS; 3 ± 9, 2 ± 4, 2 ± 2 *p*CS; 4 ± 8, 2 ± 2, 2 ± 4 Between-day precision %: IxS; 2 ± 6, 3 ± 7, 3 ± 2 *p*CS; 0.001 ± 0.001, 2 ± 2, 2 ± 3 **Recovery %** (medium and high concentrations, N = 8): By comparing the ratio of the difference in the initial concentration and the final concentration to the spiked concentration. IxS: 94 ± 9, 100 ± 8 *p*CS: 96 ± 7, 96 ± 11 **Stability:** Freeze–thaw stability at 1, 2, 3, 5, and 10 cycles, from −80 °C to room temperature, N = 8 All analytes had insignificant differences between thawing cycles except for phenylalanine at the 10th freeze–thaw cycle (+20%) **Storage conditions:** Stock solutions: −20 °C	de Loor et al. 2016 [42]
**IxS** ***p*CS**	**Matrix:** Human serum **Internal standard(s):** hydrochlorothiazide (DHCT), 2 μg/mL (final concentration for the analysis) **Column:** Agilent Zorbax SB-C18 column (3.5 μm, 2.1 × 100 mm). **Temperature(s):** Column: 30 °C **Mobile phase:** ACN and 10 mM ammonium acetate buffer (10:90, *v*/*v*) **Gradient:** Isocratic elution **Injection volume:** 5 µL **Flow rate:** 0.3 mL/min **Retention time:** IxS: 2.1 min *p*CS: 3.8 min DHCT (internal standard): 4.3 min **Total run time:** 5 min **Study authors’ sample extraction procedure:** 1. Serum samples collected after 12 h fasting in the morning; 2. transferred to coagulation cubes; 3. centrifuged at 4500× *g* for 10 min; 4. samples stored at −20 °C until analysis: - Sample preparation steps were performed for both the original serum (for total concentration) and the filtrate (for free fraction); 1. 100 µL filtrate/original serum is mixed with 200 µL ACN containing 2 µg/mL DHCT; 2. Vortex for 3 min, then centrifuge at 20,000× *g* for 5 min; 3. Supernatant is mixed with 10 mM ammonium acetate buffer (1:1, *v*/*v*) for analysis: - Filtrate preparation for free concentration measurement: Centrifuge 200 µL of the serum sample at 20,000× *g* for 5 min and prepare 100 µL of the filtrate with the sample preparation steps described above: - Calibration standards were prepared as 10X in ACN: water (10:90, *v*/*v*) and diluted by 1:10 (*v*/*v*) with 4% bovine serum albumin (BSA).	**Mass spectrometry: triple-quadrupole (Agilent)** **Mode of ionization:** negative mode **Ion source conditions:** ESI Spray voltage: 4000 V Nebulizer gas: N_2_ Nebulizer pressure: 40 psi Source temperature: 105 °C De-solvation gas: N_2_ De-solvation gas temperature: 350 °C De-solvation gas flow rate: 10 L/min Collision gas: N_2_ Collision gas pressure: 0.1 MPa **Scanning mode:** MRM ***m*/*z* transitions:** IxS: 212.2 → 80.0 Fragmentor: 130 V Collision energy: 20 eV *p*CS: 187.0 → 107.1 Fragmentor: 100 V Collision energy: 18 eV DHCT: 296.0 → 205.0 Fragmentor: 180 V Collision energy: 25 eV	**Validation criteria guideline(s):** FDA ^(d)^ **Quality control (QC) samples (low, medium, and high concentrations, μg/mL):** IxS: 1, 4, and 8 *p*CS: 0.1, 0.5, and 5 **Selectivity: confirmed for IxS, *p*CS, DHCT** **Precision and accuracy:** Intra- and inter-day precision (%):0.44–10.6 and 0.89–7.34% Accuracy (%): 95.0–105.3% **Calibration range(s):** Linearity range (μg/mL): IxS: 0.500–10.000 *p*CS: 0.050−10.000 **Quality control concentrations used in the validation (μg/mL):** IxS: 1, 4 and 8 *p*CS: 0.1, 0.5 and 5 **Stability:** 3 cycles of freeze–thaw Benchtop stability: after 6 h at room temperature. Processed sample stability: after 1 month at −20 °C Accuracy: ±10% Precision: CV < 15% **Recovery/Matrix effect:** Recovery: By comparing the protein precipitation method (using ACN or methanol) with liquid–liquid extraction. The liquid–liquid extraction recovery data are not provided by the main manuscript. The protein precipitation data using ACN are as follows (exact protocol not specified): *p*CS: 95.8–99.0% IxS: 84.7–91.8% Matrix effect: By comparing post-extraction samples with samples in the mobile phase. *p*CS: 97.9–119.9% IxS: 83.3–94.1%% **Storage:** Calibration standards and QC samples: −20 °C	Shu et al. 2016 [37]
**IxS** In addition to other analytes: anthranilic acid (AnA), indoxyl acetate (IA), KA, Kn, Trp, xanthurenic acid (XA)	**Matrix:** Human serum **Internal standard(s):** Isotope-labeled IxS-d4, final concentration 20 μg/mL in methanol. **Column:** Atlantis T3 C18, dp = 3 μm, 50 × 2.1 mm column (Waters Corporations MA, USA). **Temperature(s):** ^(b)^ **Mobile phase:** A: 5 mM ammonium acetate [the solvent was not mentioned] B: methanol **Gradient:** Negative mode: 0.0–0.5 min: 5% B 0.5–5.0 min: 5 → 95% B 5.0–8.5 min: 95 → 98% B After 8.5 min: 98 → 5% B Positive mode: 0.0–0.5 min: 5% B 0.5–8.0 min: 5 → 95% B After 8.0 min: 95 → 5% B **Injection volume:** 10 μL **Flow rate:** 0.25 mL/min **Retention time:** IxS: ~4.5 min **Total run time:** ^(b)^ **Study authors’ sample extraction procedure:** 1. Dilute a 200 μL sample with 500 μL of 0.1% formic acid with internal standards; 2. Solid phase extraction (SPE); 3. Load samples onto a 3cc HLB cartridge [the cartridge is conditioned with methanol and 1% formic acid]; 4. Wash the cartridge with 1% formic acid; 5. Elute the analytes with 1% ammonium peroxide in methanol/water (50:50); 6. Evaporate the supernatant using a N_2_ stream; 7. Reconstitute the pellet with 150 μL of 5 mM ammonium acetate. **Note:** IxS stock solution (100 μM = 21.321 μg/mL) was prepared using methanol as the main solvent and diluted with 5% bovine serum albumin/phosphate buffered saline solution.	**Mass spectrometry: triple- quadrupole (Sciex)** **Mode of ionization:** negative mode for IxS, IA, and AnA positive mode for Trp, Kn, KA, and XA **Ion source conditions:** ESI Negative mode: CAD (definitions and units for abbreviations not found in original paper): 6 CUR: 20 GS1: 45 GS2: 20 IxS: 4500 TEM: 350 DP: 48 EP: 10 Positive mode: CAD: 6 CUR: 15 GS1: 50 GS2: 40 IxS: 4000 TEM: 550 DP: 48 EP: 10 **Scanning mode:** MRM ***m*/*z* transitions:** IxxS: 213.2 → 212.0, 80.1 Collision energy: −35 V	**Validation criteria guideline(s):** FDA ^(d)^ **LLOQ:** Determined in triplicate with CV < 15% IxS: 0.1 μM equivalent to 0.02132 μg/mL **LLOD (μg/mL):** ^(b)^ **Precision and accuracy** met the acceptance criteria (± 15%) ^(d)^. Intra-assay variation based on 2 QC concentrations (~16 µM & ~46 µM) CV % = 2.357 and 3.991 Inter-assay variation based on 2 QC concentrations: CV % = 0.403 and 5.847 **Calibration range(s):** Linearity range (μM): IxS: 0.1–500 equivalents in μg/mL to 0.021–106.605, R^2^ = 0.9991 **Recovery/Matrix effect:** Recovery: Percentage of the difference in spiked and non-spiked samples to the spiked amount (33.3 and 10 µmol per original study) IxS: 108.724, 115.321% **Storage conditions: −**20 °C	Zhang et al. 2017 [43]
**IxS** ***p*CS**	**Matrix:** Human serum **Internal standard(s):** Isotope-labeled IxS-d4 and *p*CS-d7 **Column:** Acquity UPLC BEHC 18 column (2.1 × 100 mm, 1.7-μm particle size, Waters) **Temperature(s):** Column: 30 °C **Mobile phase:** A: 0.1% (*v*/*v*) formic acid in water B: 0.1% (*v*/*v*) formic acid in ACN **Gradient:** 0.0–1.0 min: 20% B 1.0–1.7 min: 20 → 80% B 1.7–1.8 min: 80 → 95% B 1.8–3.4 min: 95% B 3.5 min–5.5 min: 20% B **Injection volume:** 5 µL **Flow rate:** 0.45 mL/min **Retention time:** IxS: 1.10 min *p*CS: 1.57 min **Total run time:** 5.5 min **Study authors’ sample extraction procedure:** Total IxS and *p*CS: 1. Thaw serum samples at room temperature; 2. Mix 50 µL sample with 500 µL ACN with 0.2 mg/L IxS-d4 and *p*CS-d7 (internal standard); 3. Vortex 30 s, then centrifuge at 13,400× *g* for 15 min; 4. Dilute the supernatant 10-fold with water before injection. Free-form IxS and *p*CS 1. Centrifuge 100 µL serum for 30 min at 10,621× *g* (37 °C) using a 3000 MWCO filter (Merck Millipore); 2. 50 µL of the filtrate is subsequently processed the same way as the total concentration analysis starting from Step 2. Clinical samples 1. Centrifuge whole blood sample at 200× *g* for 10 min within 2 h of sample collection; 2. Store serum at −70 °C.	**Mass spectrometry: triple-quadrupole (Waters)** **Mode of ionization:** negative mode **Ion source conditions:** ESI Capillary voltage: 1500 V De-solvation gas flow: 150 L/h Source temperature: 150 °C **Scanning mode:** MRM ***m*/*z* transitions:** IxS: 212.04 → 80.14, 132.05 Cone voltage: 6 V Collision energy: 20/18 eV IxxS-d4: 216.04 → 80.14 Cone voltage: 6 V Collision energy: 20 eV *p*CS: 186.98 → 107.03, 80.02 Cone voltage: 4 V Collision energy: 20/16 eV *p*CS-d7: 194.04 → 114.04 Cone voltage: 8 V Collision energy: 18 eV	**Validation criteria guideline(s):** CLIA ^(e)^ **LLOQ (μg/mL):** S/N = 10, imprecision for 40 replicates over five runs is < 20%, inaccuracy < 15%. IxS: 0.05 *p*CS: 0.05 **LLOD (μg/mL):** ^(b)^ **Dilution effect:** Serum samples were prepared with a concentration of 10 mg/L for both analytes; then, it was diluted 10- and 20-fold with water (8 replicates) Imprecision < 2.8%, bias < 7.6%. **Calibration range(s):** Linearity range (μg/mL): IxS: 0.05–5.18 *p*CS: 0.05–5.12 **Precision and accuracy** Accuracy (based on concentration range 0.53–4.25 µg/mL) within 15% Precision data met the acceptance criteria (below) ^(d)^. intra- and inter-day imprecision tests Within-run, μg/mL, N = 20: IxS QC 1: 0.19 ± 0.01, CV% = 6.4 IxS QC 2: 1.98 ± 0.04, CV% = 1.9 IxS QC 3: 3.84 ± 0.09, CV% = 2.2 *p*CS QC 1: 0.42 ± 0.01, CV% = 1.9 *p*CS QC 2: 2.08 ± 0.02, CV5 = 1.1 *p*CS QC 3: 4.03 ± 0.05, CV% = 1.3 Free IxS QC 1: 0.21 ± 0.01, CV% = 5.7 Free IxS QC 2: 2.64 ± 0.05, CV% = 1.9 Free IxS QC 3: 8.01 ± 0.14, CV% = 1.8 Free *p*CS QC 1: 0.26 ± 0.01, CV% = 3 Free *p*CS QC 2: 1.54 ± 0.02, CV% = 1.5 Free *p*CS QC 3: 4.58 ± 0.04, CV% = 0.9 Between-run, μg/mL, N = 40: IxS QC 1: 0.2 ± 0.01, CV% = 6.9 IxS QC 2: 2.02 ± 0.08, CV% = 3.8 IxS QC 3: 3.96 ± 0.19, CV% = 4.7 *p*CS QC 1: 0.43 ± 0.01, CV% = 2.5 *p*CS QC 2: 2.1 ± 0.05, CV% = 2.2 *p*CS QC 3: 4.03 ± 0.15, CV% = 3.7 Free IxS QC 1: 0.21 ± 0.02, CV% = 10.1 Free IxS QC 2: 2.58 ± 0.21, CV% = 8.1 Free IxS QC 3: 7.37 ± 0.6, CV% = 8.1 Free *p*CS QC 1: 0.24 ± 0.02, CV% = 10.6 Free *p*CS QC 2: 1.46 ± 0.11, CV% = 7.8 Free *p*CS QC 3: 4.19 ± 0.35, CV% = 8.5 **Stability:** Criteria 4 °C for 7 days; −20 °C and −70 °C for 3 months (data not provided) **Carryover:** Criteria: high and low concentrations, 10 and 0.4 mg/L (data not provided) **Recovery:** Three concentrations (0.5, 1, and 4 mg/L) were prepared in distilled water and serum and analyzed for 10 replicates. IxS: 104.7% *p*CS: 101.1% **Matrix effect:** Criteria: Comparing results from 12 serum samples spiked with analytes post-extraction with samples spiked into a “neat solution” (data not provided). **Storage conditions: −**80 °C	Lin et al. 2019 [29]
**IxS** ***p*CS** In addition to other analytes: HA, KA	**Matrix**: Human serum **Internal standards**: isotope-labeled internal standards indoxyl sulfate-d4 (IxS-d4) and *p*-cresol-sulfate-d7 (*p*CS-d7), both at 5 µg/mL **Column**: Acquity BEH C18 (2.1 × 100 mm, 1.7 µm column) pre-column: Acquity BEH C18 VanGuard (2.1 × 5 mm, 1.7 µm). **Temperature**: Column: 35 °C Autosampler: 10 °C **Mobile phase**: A: 10 mM ammonium formate; pH 4.3 Formic acid: used to produce the required pH of A. B: ACN **Gradient**: isocratic elution (A: B, 85:15, *v*/*v*) **Injection volume**: 20 µL **Flow rate**: 0.3 mL/min **Retention time:** IxS: 2.11 min *p*CS: 3.30 min **Total run time:** 4 min **Study authors’ sample extraction procedure:** 1. Mix 50 µL of serum sample with 100 µL ACN containing internal standards; 2. Vortex 30 s, then centrifuge at 10,000× *g* for 5 min (temperature not provided); 3. Transfer the supernatant into a microcentrifuge tube to be evaporated under N_2_ at 40 °C; 5. Reconstitute with 150 µL water: ACN at 80:20%; 6. Vortex 30 s.	**Mass spectrometry: triple-quadrupole (Thermo Scientific)** **Mode of ionization**: ESI, switched from positive to negative mode at 1.4 min. **Ion source conditions**: Spray voltages: 3000 V (+ve) and 2000 V (−ve), vaporizer temperature: 300 °C, ion transfer tube temperature: 300 °C, collision gas pressure: 1.5 mTorr, auxiliary gas: 50 (arbitrary units), sheath gas: 60 (arbitrary units), scan time: 0.05 s, scan width: 0.05 *m*/*z*. **Scanning mode**: MRM ***m*/*z* transitions**: IxS: 212.0 → 80.4 Collision energy: 27 V IxS-d4: 215.9 → 80.4- Collision energy: 31 V *p*CS: 186.9→107.5 Collision energy: 26 V *p*CS-d7: 193.9 →114.6 Collision energy: 24 V	**Validation criteria guideline(s):** FDA ^(d)^ **LLOQ (μg/mL)**: IxS: 0.20 *p*CS: 0.25 **LLOD (μg/mL):** ^(b)^ **Calibration range(s):** Linearity range (μg/mL), correlation coefficient ≥ 0.997: IxS: 0.2–80 *p*CS: 0.25–80 **Precision and accuracy** met the acceptance criteria ^(d)^. (N = 24) Intra-day accuracy and precision: N = 12 Inter-day accuracy and precision: N = 6 IxS QC concentrations: 0.2, 0.6, 15.0, and 60.0 (μg/mL) *p*CS QC concentrations: 0.25, 0.75, 15.0, and 60.0 (μg/mL) Accuracy (% bias): Intra-day: IxS: −8.73, 10.9, 3.08, and 2.11 *p*CS: −19.3, −0.50, 3.30, and −3.70 Inter-day: IxS: −13.35, 9.50, 1.10, and 3.30 *p*CS: −17.4, 0.40, 2.80, and −5.30 Precision (% RSD): Intra-day: IxS: 9.27, 2.11, 5.46, and 6.05 *p*CS: 7.54, 3.10, 1.57, and 1.99 Inter-day: IxS: 10.9, 5.29, 5.29, and 5.51 *p*CS: 6.71, 3.46, 1.77, and 2.74 **Dilution integrity:** Highest concentrations were diluted 1:1, 1:2, and 1:4 Bias = 11.04% and RSD = 6.19% **Stability (N = 3) at LQC and HQC:** Criteria: Long-term stability: 2 months at −80 °C Benchtop stability: 6 h at room temperature. Autosampler stability: 24 h and 72 h at 10 °C Freeze–thaw stability: 3 cycles of 24 h freeze–thaw IxS RSD% at 0.6 and 60.0 μg/mL: Benchtop stability: 4.5 and 4.1 Autosampler stability: 2.9 and 3.3 Freeze–thaw stability: 3.6 and 5.4 Long-term Storage: 3.6 and 1.7 IxS %target at 0.6 and 60.0 μg/mL: Benchtop stability: 96.5 and 98.2 Autosampler stability: 98.4 and 108.9 Freeze–thaw stability: 98.8 and 106.3 Long-term Storage: 98.8 and 99.9 *p*CS RSD% at 0.75 and 60.0 μg/mL: Benchtop stability: 2.9 and 0.9 Autosampler stability: 4.1 and 1.0 Freeze–thaw stability: 1.7 and 5.8 Long-term Storage: 1.7 and 2.0 *p*CS %target at 0.75 and 60.0 μg/mL: Benchtop stability: 102.3 and 97.6 Autosampler stability: 104.6 and 100.9 Freeze–thaw stability: 97.3 and 103.2 Long-term Storage: 97.5 and 101.1 **Recovery/Matrix effect:** Recovery: By comparing samples spiked into the matrix and then extracted to samples spiked into the post-extracted matrix. IxS at 0.6, 15.0, and 60.0 μg/mL: 106, 99.3, 97.4% *p*CS at 0.75, 15.0, and 60.0: 92.8, 87.7, 89.1% Matrix effect (Nn = 3) at 3 QCs: By comparing samples spiked into the post-extracted matrix to samples spiked into the water without extraction. IxS at 0.6, 15.0, and 60.0 μg/mL: 103, 94.7, and 98.4% *p*CS at 0.75, 15.0, and 60.0: 96.2, 100, and 93.1% **Storage conditions: −**80 °C	Prokopienko et al. 2019 [44]
**IxS** ***p*CS** In addition to other analytes: CMPF, HA, IAA, *p*CG, TMAO	**Matrix:** Human serum **Internal standard(s):** Isotope-labeled indoxyl sulfate-^13^C_6_ ([^13^C_6_-IxS) and *p*-cresol sulfate-d4 (*p*CS-d4), 500 ng/mL for all internal standards. **Column:** Ultra PFP Propyl column, (5 μm, 50 × 2.1 mm; Restek, Lisses, France). Pre-column: ultra PFP propyl precolumn (5 μm, Restek) **Temperature(s):** 40 °C **Mobile phase:** A: 0.1% (*v*/*v*) formic acid in water B: 0.1% (*v*/*v*) formic acid in ACN **Gradient:** In the negative ionization mode: 0.0–1.0 min: 40% → 80% B 1.0–2.0 min: 80% B 2.0–2.2 min: 80% → 40% B 2.2–2.5 min: 40% B A different gradient was applied in the positive ionization mode (not applicable to IxS and PCS). **Injection volume:** 15 µL for each mode **Flow rate:** 0.8 mL/min **Retention time:** IxS: ~0.50 min extracted from the provided chromatograms *p*CS: ~0.54 min extracted from the provided chromatograms **Total run time:** Negative mode: 2.5 min Positive mode: 2 min **Study authors’ sample extraction procedure:** 1. Mix a 50 µL sample with 200 µL of an iced ACN solution containing the internal standards; 2. Vortex, then centrifuge at 16,500× *g* for 15 min; 3. Dilute 50 µL of the supernatant with 20-fold water.	**Mass spectrometry: QTRAP tandem mass (Sciex)** **Mode of ionization:** negative and positive mode; negative for IxS and *p*CS **Ion source conditions:** ESI Voltage: −4500 V (negative mode) and 4500 (positive mode) Ion source temperature: 350 °C Heater gas: 70 psi Nebulizer gas: 40 psi Curtain gas: 30 psi **Scanning mode:** MRM ***m*/*z* transitions:** IxS: 211.9 → 79.9, 132 Declustering potential: −55 V Entrance potential: −2.5 V Cell entrance potential: −10 V Collision energy: −34 and −24 eV Collision cell exit potential: 0 V [^13^C_6_]-IxS: 217.7 → 79.7, 138 Declustering potential: −65 V Entrance potential: −9.5 V Cell entrance potential: −12 V Collision energy: −34 and −24 eV Collision cell exit potential: 0 V *p*CS: 186.8 → 106.8, 79.9 Declustering potential: −40 V Entrance potential: −2.5 V Cell entrance potential: −12 V Collision energy: −30 eV Collision cell exit potential: 0 V d4- *p*CS: 190.8 → 111, 79.9 Declustering potential: −30 V Entrance potential: −2.5 V Cell entrance potential: −14 V Collision energy: −30 eV Collision cell exit potential: 0 V	**Validation criteria guideline(s):** EMA ^(f)^ and SWGTOX ^(g)^ **LLOQ (μg/mL)**: IxS: 0.1 *p*CS: 0.1 **LLOD (μg/mL)**: IxS: 0.04 *p*CS: 0.006 **IxS and *p*CS calibration range (μg/mL)**: 0.1–100 for all toxins except indole-3-acetic acid IxS R^2^ = 0.9996 ± 0.0004 *p*CS R^2^ = 0.9999 ± 0.00007 **Inter-sample carryover**: < 1% **Precision and accuracy**: both within-run and between-run met the acceptance criteria. IxS and *p*CS QCs: 0.5, 2.5, 25, and 75 IxS CV% and bias%: Within run: At 0.5 μg/mL: 3.85 and 3.80 (CV% and bias%) At 2.5 μg/mL: 6.82 and −9.84 At 25 μg/mL: 10.73 and −8.16 At 75 μg/mL: 7.74 and −10.29 Between run: At 0.5 μg/mL: 8.47 and 6.10 (CV% and bias%) At 2.5 μg/mL: 14.23 and −9.84 At 25 μg/mL: 10.62 and −14.49 At 75 μg/mL: 14.54 and −14.79 *p*CS CV% and bias%: Within-run: At 0.5 μg/mL: 10.66 and 2.67 (CV% and bias%) At 2.5 μg/mL: 3.03 and 14.48 At 25 μg/mL: 2.98 and 9.07 At 75 μg/mL: 3.02 and 10.33 Between-run: At 0.5 μg/mL: 12.92 and −4.48 (CV% and bias%) At 2.5 μg/mL: 5.51 and 9.73 At 25 μg/mL: 6.10 and 2.98 At 75 μg/mL: 6.23 and 12.62 **Stability as the concentration loss%:** IxS (6 h at 4 °C, 24 h at 4 °C, 1 month at −20 °C, and 3 freeze–thaw cycles): At 2.5 μg/mL: 4.08, 9.48, 2.64, and 25.57% At 25 μg/mL: 6.32, 3.23, 2.73, and 20.86 At 75 μg/mL: 2.83, 0.80, 2.49, and 26.13 *p*CS (6 h at 4 °C, 24 h at 4, 1 month at −20 °C, 3 freeze–thaw cycles): At 2.5 μg/mL: 9.97, 2.62, 4.41, and 4.80% At 25 μg/mL: 5.65, 5.99, 8.48, and 3.33% At 75 μg/mL: 7.65, 4.60, 10.37, and 8.05% **Selectivity:** No interference reported **Recovery/Matrix effect:** Matrix effect at 25 and 75 µg/mL: By comparing samples spiked into extracted blank matrix cleaned with activated charcoal to samples spiked into a neat solution. IxS: 87.33, 74.40% *p*CS: 76.87, 67.02% Recovery: not provided **Storage conditions:** Stock: **−**20 °C	André et al. 2020 [45]
**IxS** ***p*CS** In addition to other analytes: CMPF, HA, KA, Kn, IAA, PAG, *p*CG, Phe, TMAO, Trp, Tyr.	**Matrix:** Human serum **Internal standard(s):** isotope-labeled IxS-d4 (0.1 μg/mL) and *p*CS-d7 (0.01 μg/mL) **Column:** Accucore PFP column, 100 × 2.1 mm, 2.6 μm (Thermo, Les Ulis, France) **Temperature(s):** Column: 40 °C **Mobile phase:** A: 0.1% *v*/*v* formic acid in water B: ACN **Gradient:** 0.0–1.0 min: 1% B 1.0–7.5 min: 1 → 65% B 7.5–7.6 min: 65 → 90% B 7.6–9.0 min: 90% B 9.0–10.9 min: 1% B **Injection volume:** 4 µL **Flow rate:** 0.5 mL/min **Retention time:** IxS: 5.75 min *p*CS: 6.18 min **Total run time:** ^(b)^ **Study authors’ sample extraction procedure:** Collecting blood samples: 1. Collection samples in a vacutainer tube; 2. Centrifuge 3000× *g* at 4 °C for 10 min Total concentration: 1. Mix 50 μL of serum sample with 25 μL of internal standard solution (deuterated internal standards in methanol, IxS-d4 (0.1 μg/mL) and *p*CS-d7 (0.01 μg/mL), in addition to the other internal standards; 2. Add 340 μL of methanol for precipitation; 3. Vortex and centrifuge at 9000× *g* and 4 °C for 10 min; 4. Evaporate the supernatant using an N_2_ stream; 5. Reconstitute the dry residue with 80 µL water. Free-form concentration: Ultrafiltration: 1. Pass a 150 µL serum sample into the ultra-centrifugal filter (30 KDa, 0.5 mL, Amicon, Germany); 2. Centrifuge at 13,300× *g* for 20 min; 3. Obtain the filtrate for the free form of uremic toxin	**Mass spectrometry: triple-quadrupole (Thermo)** **Mode of ionization:** alternating negative and positive; negative for IxS and *p*CS **Ion source conditions:** ESI Nebulization gas: N_2_ Collision gas: Ar Source temperature: 350 °C Vaporization temperature: 100 °C Capillary voltage: 5000 V (positive mode) and 4000 V (negative mode) **Scanning mode:** MRM ***m*/*z* transitions** IxS: 212.1 → 80.0, 81.0 Collision energy: 23 V (80.0 fragments), 17 V (81.0 fragments) *p*CS: 187.1 → 107.1, 105.1 Collision energy: 21 V (107.1 fragments), 34 V (105.1 fragments)	**Validation criteria guideline(s):** EMA ^(f)^ **LLOQ (μg/mL):** IxS: 0.001 *p*CS: 0.001 **LLOD (μg/mL):** ^(b)^ **Calibration range(s), N = 6:** **(μg/mL):** IxS: 0.001–50, R^2^ = 0.9993 ± 0.0006 *p*CS: 0.001–50, R^2^ = 0.9995 ± 0.0004 **QC concentrations:** 0.030, 0.150, 8.000, and 40.000 µg/mL. **Precision and accuracy** met the acceptance criteria ^(g)^, for all the analytes. Intra-day, N = 6: CV% per QCs: IxS: 8.8, 4.3, 10.1, 7.6% *p*CS: 7.8, 4.9, 7.0, 8.1% Bias% per QCs: IxS: 3.3, 6.7, −1.7, 6.7% *p*CS: 10.0, 0.0, 2.4, 1.9% Inter-day, N = 6: CV% per QCs: IxS: 3.3, 5.9, 7.0, 10.9% *p*CS: 4.4, 6.6, 8.0, 6.4% Bias% per QCs: IxS: 1.3, 3.3, −5.1, −2.2% *p*CS: 10.0, −1.3, −2.2, −1.8% **Stability:** Criteria: 1 week at 4 °C 1 week, 2 weeks, and 2 months at −80 °C 1, 2, 3, 4, and 5 freeze–thaw cycles. (numerical data not provided) **Recovery/Matrix effect:** Matrix effect at 0.1 and 1 μg/mL (N = 6): By comparing samples spiked in a matrix to samples spiked in water. IxS-d4: Serum: 113.0, 103.9% Buffer: 86.1, 99.2% ultrafiltrate: 113.6, 106.9% *p*CS-d7: Serum: 85.2, 97.6% Buffer: 96.0, 106.4% Ultrafiltrate: 100.0, 97.7% **Recovery:** not provided **Storage conditions:** Blood samples: −80 °C Stock: −20 °C	Fabresse et al. 2020 [31]
**IxS** ***p*CS** In addition to other analytes: N-acetyl-l-arginine, CMPF, Creatinine, 3-deoxyglucosone, 3-(3,4-dihydroxyphenyl)-L-alanine, N2,N2-dimethyl guanosine, DL-Homocysteine, 4-ethylphenyl sulfate, HA, IAA, 3-indolyl- -d-glucopyranoside, KA, Kn, 1-methyl-inosine, N6-(carboxymethyl)-l-lysine N-acetylcytidine, Orotic acid, PAG, pCG, PG, Pseudo uridine, S-adenosyl-l-homocysteine, symmetric dimethylarginine, Uridine.	**Matrix:** Human serum **Internal standard(s):** isotope-labeled hippuric acid-d5 (HA-d5), stock solution concentration (22.230 μg/mL) **Column:** Agilent Poroshell 120 HILIC column (4.6 × 100 mm, 2.7 µm) **Temperature(s):** Column: 30 °C **Mobile phase**: A: 0.1% acetic acid in water B: ACN **Gradient**: isocratic elution A: B (60:40, *v*/*v*). **Injection volume**: 10 µL **Flow rate**: 0.6 mL/min **Retention time**: IxS: 1.47 min *p*CS: 1.39 min **Total run time:** 8 min/each ion mode (total = 16 min) **Study authors’ sample extraction procedure:** Blank serum preparation: Mix healthy volunteer serum and activated charcoal. Preparation of clinical samples: 1. Centrifuge blood samples at 940× *g* for 12 min to obtain serum 2. Store the serum at −80 °C. Total toxin concentration: 1. Mix 50 µL of the sample with 50 µL of the internal standard mixture; 2. Add 100 µL ACN and vortex for 40 s; 3. Centrifuge at 18,000× *g* for 10 min. Free-form concentration: 1. Add 400 µL serum to an ultrafiltration device (Centrifree membrane, 30 kDa); 2. Centrifuge at 3500× *g* at 37 °C for half an hour; 3. Follow the same procedure as for the total concentration above.	**Mass spectrometry: triple-quadrupole (Agilent)** **Mode of ionization**: negative and positive; negative for IxS and *p*CS **Ion source conditions**: Drying gas temperature: 350 °C Drying gas flow rate: 9 L/min Nebulizer gas pressure: 0.35 MPa Capillary voltage—positive: 4000 V Capillary voltage—negative: 3500 V **Scanning mode**: MRM ***m*/*z* transitions**: IxS: 212.4 → 80.1 (collision energy 14 eV) *p*CS: 187.4 → 80.1 (collision energy 30 eV) HA-d5: 183.4 → 139.4 (collision energy 6 eV)	**Validation criteria guideline(s):** Not specified **LLOQ (μg/mL):** IxS: 0.02014 *p*CS: 0.01954 **LLOD (μg/mL):** ^(b)^ **Calibration range (μg/mL):** IxS: 0.08057–165.000 *p*CS: 0.07817–160.000 **QC concentrations for LLOQ, LQC, MQC, and HQC (μg/mL):** IxS: 0.02014, 0.100, 10.000, and 80.000 *p*CS: 0.01954, 0.100, 20.000, and 80.000 **Selectivity and specificity:** Comparing the response in LLOQ between blank samples and clinical samples (based on < 20% interference). No numerical data were provided but passed validation per the authors. **Accuracy for LQC, MQC, and HQC:** Intra-day: IxS: 106.4, 95.3, and 100.2% *p*CS: 103.5, 90.4, and 93.8% Inter-day: IxS: 104.1, 99.7, and 95.0% *p*CS: 110.7, 99.3, and 99.0% **Precision for LQC, MQC, and HQC:** Intra-day: IxS: 7.64, 10.49, and 4.29% *p*CS: 7.22, 14.43, and 3.95% Inter-day: IxS: 2.01, 14.08, and 12.73% *p*CS: 6.99, 10.03, and 5.08% **Carryover and dilution integrity (10 and 100X)** met the acceptance criteria for *p*CS and IxS **Stability (accuracy%; precision RSD%) at** 1 h at 4 °C 24 h at room (temperature inconsistent between text and table) Two weeks at −80 °C Three freeze–thaw cycles (−80 °C) Results: LQC: IxS: (102.36%; 3.45%), (88.74%; 6.27%), (97.68%; 4.92%), and (108.94%, 2.36%) *p*CS: (98.56; 2.16%), (98.78; 1.37%), (102.79%; 3.21%), and (108.36%; 10.62%) MQC: IxS: (101.97%; 2.79%), (87.62%; 3.49%), (89.02%; 8.33%), and (103.26%; 7.99%) *p*CS: (97.89%; 2.67%), (97.65%; 0.83%), (97.65%; 2.98%), and (103.67%; 11.39%) HQC: IxS: (97.68%; 3.95%), (93.45%; 5.91%), (102.38%; 1.72%), and (106.53%; 1.03%) *p*CS: (101.54%; 0.96%), (102.34%; 1.96%), (100.46%; 3.86%) and (108.94%; 8.91%) **Recovery/Matrix (%) effect for LQC, MQC, and HQC:** (a) Samples spiked to the matrix and extracted; (b) Samples spiked into water and ACN; (c) Samples spiked into the post-extracted matrix. Recovery (%), (a:c): IxS: 85.24 ±1.09; 87.87 ±0.76; 95.99 ±9.62 *p*CS: 92.37 ±2.31; 98.46 ±3.89; 102.34 ±3.89 Matrix effect (%), (c:b): IxS: 102.25 ±2.06; 104.46 ±1.98; 96.14 ±3.29 *p*CS: 103.41 ±5.74; 107.98 ±6.89; 98.74 ±0.96 **Dilution integrity for 10 and 100 folds (accuracy%; precision, RSD%):** IxS: (98.8; 6.09) and (95.5; 12.67) *p*CS: (103.2; 0.41) and (103.0; 5.98) **Storage conditions:** Stock storage: −20 °C Separated serum storage: −80 °C	Ma et al. 2020 [46]
**IxS** In addition to other analytes: CMPF, HA, 4-hydroxyhippuric acid, IAA, KA, Kn, Melatonin.	**Matrix**: Human serum **Internal standard(s)**: Hydrochlorothiazide (DHCT), 30 ng/mL **Column**: Waters Atlantis T3 column 2.1 mm × 100 mm, 3 µm (Milford, MA, USA) **Temperature(s)**: Column: 35 °C **Mobile phase**: A: Water with 0.1% (*v*/*v*) formic acid and 10 mM ammonium acetate. B: ACN **Gradient**: 0.0–5.0 min: 15 → 85% B 5.0–7.0 min: post-run **Injection volume**: 5 µL **Flow rate**: 0.3 mL/min **Retention time**: between 2–2.5 min (extracted from the provided chromatograms) **Total run time:** 5 min **Study authors’ sample extraction procedure:** 1. Mix 100 µL sample with 300 µL ACN with the internal standard; 2. Vortex 1 min; 3. Centrifuge at 13,400× *g* and at room temperature for 10 min.	**Mass spectrometry: triple-quadrupole (Agilent)** **Mode of ionization**: negative and positive mode; negative for IxS **Ion source conditions**: ESI Spray voltage: ±4000 V Nebulizer pressure: 50 psi Drying gas temperature: 350 °C, Drying gas flow rate: 10 L/min, Temperature of sheath gas: 350 °C, Sheath gas flow rate: 12 L/min. Collision gas: N_2_ **Scanning mode**: MRM ***m*/*z* transitions**: IxS: 212.0 → 132.1 Collision energy: 18 DHCT: 295.8 → 205 Collision energy: 21	**Validation criteria guideline(s):** Not specified **LLOQ (μg/mL)**: 0.156250 **LLOD (μg/mL):** ^(b)^ **Calibration range(s):** Linearity range (μg/mL): IxS: 0.156250–20.000 **Specificity:** Criteria: Chromatograms from blank PBS, spiked PBS, and clinical samples were compared (Nn = 6) No interference reported **Carryover:** Criteria: Injecting a blank sample after the highest concentration sample (N = 3) No carryover was reported. **Intra- and inter-day accuracy and precision** met the acceptance criteria for all analytes. LLOQ, low-, medium-, and high-quality control concentrations (N = 5) were used for inter- and intra-day precision (RSD%) and accuracy as relative error % (RE%). Concentrations (μg/mL): 0.15625, 0.3125, 1.250, and 10.000. Intra-day (N = 5): RE%: 8.40, 4.23, 1.64, and −4.40% RSD%: 8.20, 10.85, 5.25, and 7.32% Inter-day (N = 5): RE%: 2.03, 2.47, −0.92, and −4.72% RSD%: 13.76, 9.94, 9.30, and 2.44% **Stability, (N = 5):** QC samples **(μg/mL)**: 0.3125, 1.250, 10.000 Criteria: Autosampler at 4 °C for 24 h Freeze–thaw (3 cycles) −20 °C Long-term 3 months at −80 °C Autosampler stability: 24 h at 4 °C RE%: 3.43, 1.28, 7.47% 3 cycles of freeze–thaw at–20 °C RE%: 1.98, −0.85, 4.92% long term stability: 3 months at −80 °C RE%: 1.96, 2.07, 4.37% **Recovery, 3 QC samples, N = 3:** Response in samples spiked with analytes before extraction procedure to samples spiked with analytes after extraction procedure. QC samples **(μg/mL)**: 0.3125, 1.250, and 10.000 IxS: 74.69 ± 4.87; 69.27 ± 1.85; 70.31 ± 9.86% **Matrix effect, 3 QC samples, N = 3:** Response in samples spiked with analytes in the blank matrix after extraction compared to samples spiked with analytes at the same concentrations in the neat solvent. QC samples **(μg/mL)**: 0.3125, 1.250, and 10.000 IxS: 119.57 ± 13.61; 125.30 ± 12.53; 112.01 ± 11.98% **Storage conditions:** −80 °C	Wang et al. 2020 [47]
**IxS,** ***p*CS,** In addition to dihydroepiandrosterone sulphate, ethyl-β-D-glucuronide, ethyl sulfate, estrone sulfate resveratrol, L-tartaric acid.	**Matrix:** serum, plasma, and urine **Internal standards:** indoxyl sulphate-d4 potassium salt (IxS-d4) and *p*-cresol sulphate potassium salt-d7 (*p*CS-d7) **Column:** Waters Acquity™ UPLC HSS T3 1.8 μm 2.1 × 100 mm Pre-column: Waters Acquity™ UPLC HSS T3 1.8 μm VanGuard 2.1 × 5 mm **Column temperature:** 40 °C **Mobile phase:** A: 0.075% (*v*/*v*) formic acid in mQwater. B: methanol. **Composition gradient:** 0.00–1.00 min: 2% B 1.00–1.50 min: 2–25%B 1.50–1.90 min: 25–50%B 1.90–4.10 min: 50–80%B 4.10–4.50 min: 80–100%B 4.50–4.90 min: 100%B 4.90–5.10 min: 100–2%B 5.10–6.00 min: 2%B **Injection volume:** 7.5 μL **Flow rate (mL/min):** 0.0–1.50 min: 0.55 1.50–4.10 min: 0.43 4.10–5.10 min: 0.60 5.10–6.00 min: 0.55 **Retention time:** IxS: 2.54 min IxS-d4: 2.53 min *p*CS: 2.68 min *p*CS-d7: 2.80 **Total run time:** ^(b)^ **Study authors’ sample extraction procedure:** Urine samples: 1. Collected and stored at −80 °C (up to 36 months); 2. Thawed samples were centrifuged at 10,000 crf for 3 min; 3. Dilute supernatant by 15-fold using mobile phase A containing the internal standards. Blood samples (plasma and serum): 1. Serum isolated from blood; 2. Stored at −20 °C (for up to 1–14 weeks). The following steps are according to the authors’ previous publication [48]: 3. Thaw plasma samples on ice; 4. Mix 40 µL of the sample with 180 µL of 90% methanol 0.1% formic acid in a 96-well Sirocco™ plasma protein filtering plate; 5. Vortex for 5 min; 6. Keep at 4 °C for 10 min, then 5 min re-equilibration at room temperature; 7. Place an ultra-performance liquid chromatography (UPLC) autosampler 96-well plate underneath the protein filtering plate; 8. Apply a vacuum to collect the filtrate; 9. Once the filtering plate is dry, re-extract with 180 µL of a 20:80 acetone: ACN mixture with 0.1% formic acid; 10. Repeat Step 8; 11. Evaporate the solvent/filtrates in the UPLC plates using a cooled vacuum centrifuge; 12. Reconstitute with 200 µL mQ acidic water.	**Mass spectrometry: tandem mass (Waters).** **Mode of ionization**: negative **Ion source conditions**: ESI **Scanning mode**: MRM collision gas: Argon capillary voltage: 3.50 kV source temperature: 150 °C desolvation temperature: 350 °C desolvation gas flow: 900 L/h cone gas flow: 50 L/h ***m*/*z* transitions:** IxS: 212→80, 132 IxS-d4: 216→80, 136 *p*CS: 186.7→107, 80 *p*CS-d7: 194.1→114.1, 80 **Cone voltage and collision energy**: IxS: 30 and 20 IxS-d4: 30 and 20 *p*CS: 30 and 20 *p*CS-d7: 30 and 20	**Validation criteria guideline(s):** ICH ^(h)^, EMA ^(f)^, and FDA ^(d)^ **LLOD (μg/mL):** IxS: 0.0002 μg/mL *p*CS: 0.003 μg/mL **LLOQ (μg/mL):** IxS: 0.019 μg/mL *p*CS: 0.024 μg/mL **Linearity range (μg/mL):** IxS: 0.019–9.86, R^2^ = 0.9992 *p*CS: 0.024–12.47, R^2^ = 0.9960 **Accuracy/Bias:** data not provided **Repeatability/Precision (CV%), (N = 6):** IxS: 2.5 μg/mL *p*CS: 0.88 μg/mL in urine and plasma. CV% for urine and plasma: IxS: 2.1 and 2.7% *p*CS: 2.1 and 3.8% **Intermediate precision (inter-day):** The same QC concentrations as the “repeatability” experiment Triplicates on 3 separate days, total N = 9. CV% urine and plasma: IxS: 1.8 and 1.9% *p*CS: 2.3 and 19% **Recovery:** By comparing the spiked samples in the matrix to nominal values at several concentrations. In urine for the highest concentration tested: IxS: 114% *p*CS: 90% In plasma for the highest concentration tested: IxS: between 82% and 114% (exact value not provided) *p*CS: 82% **Matrix effect:** By comparing the slope of the calibrators set prepared in the matrix to the same set prepared in solvent. The following equation was used: (slope in matrix/slope in solvent) × 100%) − 100% In urine: IxS: 10.0% *p*CS: 23.1% In plasma: IxS: 15.6% *p*CS: 26.9% In serum: IxS: 10.0% *p*CS: 9.6% **Stability:** Criteria: 4 weeks storage at −20 °C Freeze thaw: 3 cycles 5 °C for 15–20 h autosampler No decrease in sample integrity was reported **Storage**: −20 °C (validated criteria)	Monosık, R. et. al. 2016 [49]
**IxS** In addition to other analytes: AnA, 3-hydroxykynurenine, 3-hydroxy anthranilic acid, 5-hydroxy-indole-3-acetic acid, IAA, indole-3-acetyl-glycine, indole-3-lactic acid, indole-3-propionic acid, KA, Kn, melatonin, nicotinamide, nicotinic acid, quinolinic acid, serotonin, tryptamine, Trp, XA	**Matrix**: Human serum, human urine, cell culture (monocytes and dendritic cells) **Internal standard**: Isatin for IxS (1 μM) **Column**: Atlantis T3 (2.1 × 150-mm i.d., 3 μm) reversed-phase column (Waters, Eschborn, Germany) **Temperature(s):** Column: 25 °C. **Mobile phase**: A: Water with 0.1% formic acid B: ACN with 0.1% formic acid **Gradient**: 0.0–1.0 min: 0.0 → 50% B 1.0–6.0 min: 50% B 6.0–6.1 min: 50 → 0.0% B 6.1–10.1 min: 0.0% B A different gradient was used for the positive mode. **Injection volume**: 10 μL **Flow rate**: 0.4 mL/min. **Retention time:** Isatin: 4.86 min IxS: 5.47 min **Total run time:** 10.1 min based on gradient conditions **Study authors’ sample extraction procedure**: Preparation of serum and cell culture supernatant: 1. Thaw samples at room temperature; 2. Within 30 min of thawing, add 10 µL of the internal standard mixture to a 50 µL sample; 3. Dilute with water with 0.1% formic acid up to 100 µL; 4. Vortex; 5. Add 400 μL of cold methanol; 6. Vortex; 7. Incubate at −20 °C for 1 h; 8. Centrifuge at 10,000× *g* and 4 °C for 10 min; 9. Centrifuge the supernatant again; 10. Dry the supernatant with a vacuum evaporator; 11. Dissolve the residue in 100 µL of 0.1% formic acid in water; 12. Centrifuge at 5000× *g* and 4 °C for 5 min; Preparation of urine samples: 1. Mix 10 µL of urine sample with 10 µL of the internal standard mixture. 2. Dilute the sample up to 100 µL with water containing 0.1% formic acid. 3. Centrifuge at 10,000× *g* and 4 °C for 10 min.	**Mass spectrometry: QTrap (Applied Biosystems)** **Mode of ionization**: negative mode and positive mode, negative for IxS and Isatin **Ion source conditions**: ESI Ion spray voltage: −4500 V (negative mode), 5500 V (positive more) Curtain gas: 10 psig Ion source temperature: 500 °C Ion source gas 1 and 2: 50 psig Collision gas: medium. (not specified) **Scanning mode**: MRM ***m*/*z* transitions**: isatin: 145.9 → 118.0 Declustering potential: −60 Collision energy: −30 Cell exit potential: −11 IxS: 212.0 → 80.0 Declustering potential: −45 Collision energy: −16 Cell exit potential: −21	**Validation criteria guideline(s):** FDA ^(d)^ **LLOQ (μg/mL):** 0.001 **LLOD (μg/mL):** 0.0002 **Linearity range (μg/mL):** 0.001–8.528, R^2^ = 0.9991 **Stability:** ^(b)^ **Recovery concentrations (based on the *“slope”* method according to the original manuscript):** **In serum and cell culture, QCs (μg/mL):** Low: 1 μM = 0.21321 μg/mL Medium: 2 μM = 0.42642 μg/mL High: 4 μM = 0.85284 μg/mL **In urine:** not performed Slope 0.79–1.23 (1 indicating 100% recovery based on authors’ definition) **Matrix effect defined in *“recovery”* (according to the original manuscript):** **In urine, QC:** Low: 0.1 μM = 0.0213 μg/mL Medium: 0.2 μM = 0.0426 μg/mL High: 0.4 μM = 0.0852 μg/mL Recovery for IxS between 99–115% **In Serum:** 2 μM = 0.42642 μg/mL Relative recovery: recovery (%) based on analyte/internal standard area ratio: 90.43% Absolute recovery (%) based on analyte area: 64.22% **Storage:** Stock solutions storage: −80 °C	Zhu et al. 2011 [32]
**IxS** ***p*CS** In addition to other analytes: Creatinine, 4-ethylphenyl sulfate, 1-methyladenosine, PS, TMAO	**Matrix**: Human plasma/serum **Internal standards**: isotope-labeled IxS-d4 (100 ng/30 µL) and *p*CS-d4 (50 ng/30 µL) **Column**: Scherzo SS-C18 (2 mm i.d. × 50 mm, 3 μm, Imtakt) Also tested: XSELECT CSH C18 (2.1 mm i.d. × 150 mm, 3.5 μm; Waters) Luna Phenyl-Hexyl (2.0 mm i.d. × 100 mm, 3 μm; Phenomenex) SeQuant ZIC-HILIC (2.1 mm i.d. × 50 mm, 3.5 μm; Merck) Scherzo SM-C18 (2 mm i.d. × 50 mm, 3 μm; Imtakt) Scherzo SW-C18 (2 mm i.d. × 50 mm, 3 μm; Imtakt) Pre-column: (2 mm i.d. × 5 mm, 3 μm) **Temperature:** Column: 55 °C **Mobile phase**: A: 0.1% acetic acid/20% ACN in water B: 10 mM ammonium acetate/80% ACN in water **Gradient**: 0.0–1.0 min: 25% B 1.0–4.0 min: 25 → 34% B 4.0–4.1 min: 34 → 60% B 4.1–16.0 min: 60% B 16.0–17.5 min: 100% B 17.5–20.0 min: 100 → 25% B **Injection volume**: 1 µL **Flow rate**: 0.2 mL/min; increased to 0.6 mL/min between 16.1 and 18.5 min **Retention time**: For XSELECT CSH C18, Luna Phenyl-Hexyl, SeQuant ZIC-HILIC, Scherzo SM-C18, Scherzo SW-C18, and Scherzo SS-C18, respectively IxS: 9.66, 8.12, 1.67, 6.60, 6.17, 12.66 min *p*CS: 10.42, 8.86, 1.19, 6.87, 6.65, 11.51 min **Total run time:** 20 min **Study authors’ sample extraction procedure**: 1. Mix 10 µL of plasma/serum with 30 μL of internal standard mixture and 100 µL of ACN containing 0.1% formic acid; 2. Sonicate the mixture for 10 min; 3. Centrifuge at 16,400× *g* and 4 °C for 15 min; 4. Aliquot 100 µL of supernatant and evaporate to dryness under N_2_ (15 min, 60 °C); 5. Reconstitute the dry sample with 20 µL of 30% ACN in water.	*Note: To enhance ionization, post-column addition of ACN with flow rate 0.1 mL/min, 0.4 mL/min between 1 and 16 min* **Mass spectrometry: triple-quadrupole (Thermo Fisher Scientific)** **Mode of ionization**: positive mode [0.0–6.5 min] and negative mode [6.5–20 min]. **Ion source conditions**: ESI Nebulizing and de-solvation gas: N_2_ Collision gas: Ar Capillary voltage: 4000 V in positive mode and 2500 V in negative mode Sheath gas pressure: 60 psi Auxiliary gas pressure: 60 psi Vaporizer temperature: 500 °C Capillary temperature: 350 °C Collision gas pressure: 2.0 mTorr **Scanning mode**: MRM ***m*/*z* transitions, collision energy (CE), and tube lens (TL)**: IxS: 212.1 → 131.9 CE:24 TL: 95 IxS-d4: 216.0 → 136.0 CE: 24 TL: 95 *p*CS: 186.8 → 106.9 CE: 26 TL: 73 *p*CS-d4: 191.0 → 111.1 CE: 26 TL: 73	**Validation criteria guideline(s):** Not specified **LLOQ (μg/mL):** IxS: 1.9 μM = 0.405 μg/mL *p*CS: 2.7 μM = 0.508 μg/mL **LLOD (μg/mL):** ^(b)^ **Linear range (μg/mL):** IxS: 0.405–41.490, R = 0.998 *p*CS: 0.508–24.993, R = 0.999 **Intra- and inter-day accuracy and precision:** intra-day precision (%) and accuracy (%) (N = 5): IxS (1.95, 7.79, 19.5, and 195 μM = 0.416, 1.661, 4.158, and 41.576 μg/mL**)**: LLOQ: −8.3 and 5.5% Low QC: −8.2 and 2.4% Medium QC: −8.8 and 1.4% High QC: 0.6 and 0.6% *p*CS (6.64, 13.3, 26.6, 66.4 μM = 1.250, 2.503, 5.006, and 12.496 μg/mL): LLOQ: −8.4 and 2.5% Low QC: −3.0 and 1.9% Medium QC: 1.0 and 2.2% High QC: −3.6 and 2.1% Inter-day precision (%) and accuracy (%) (N = 15): IxS: LLOQ: −7.2 and 9.4% Low QC: −10.0 and 3.8% Medium QC: −8.6 and 2.9% High QC: −1.7 and 3.5% *p*CS: LLOQ: −4.8 and 6.7% Low QC: −7.4 and 4.4% Medium QC: −1.5 and 5.7% High QC: −1.7 and 6.6% **Stability (“mid-range” concentration):** Benchtop stability (60 °C, room temperature, and on ice) for 0.5, 1, 3, and 24 h before sample extraction (N = 4). On ice (0.5, 1, 3, and 24 h): IxS: 108.9 ± 2.1%, 103.7 ± 2.6%, 103.3 ± 4.5%, and 92.2 ± 2.5% *p*CS: 100.6 ± 1.3%, 102.5 ± 1.9%, 107.6 ± 1.9%, and 100.7 ± 1.5% At room temperature: IxS: 109.7 ± 2.7%, 103.8 ± 2.5%, 100.2 ± 5.5%, and 100.9 ± 6.8% *p*CS: 101.5 ± 0.9%, 103.5 ± 1.4%, 108.0 ± 2.3%, and 100.0 ± 1.8% At 60 °C: IxS: 102.1 ± 3.1%, 97.9 ± 5.7%, 93.5 ± 4.0%, and 102.7 ± 1.7% *p*CS: 99.0 ± 2.3%, 104.2 ± 3.1%, 104.5 ± 0.7%, and 103.7 ± 2.9% Processed sample stability (60 °C) for (1 and 3 h) after deproteinization. IxS: 102.3 ± 3.6% and 106.3 ± 2.8% *p*CS: 98.9 ± 3.6% and 95.3 ± 1.3% **Matrix effect:** Based on a numerical factor calculated as the ratio of the slope between spiked sample matrix to spiked water. IxS: 1.00 *p*CS: 0.99 **Storage:** Stock solution storage: −20 °C	Kanemitsu et al. 2017 [50]
**PLASMA**				
***p*** **CS**	**Matrix**: Human plasma **Internal standards**: *p*-toluene sulfonic acid sodium salt (*p*TS) **Column**: Zorbax SB-C18 column 210 × 30 mm I.D., 3.5 μm, (Agilent Technologies, Waldbronn, Germany). **Temperature(s):** Column: 40 °C Autosampler: 4 °C **Mobile phase**: A: 99.9% water/0.1% formic acid B: 99.9% ACN/0.1% formic acid **Gradient**: 0.0–2.0 min: 20% B 2.0–3.0 min: 20 → 95% B 3.0–7.0 min: 95% B 7.0–7.5 min: 95 → 20% B **Injection volume**: 1 µL **Flow rate**: 0.2 mL/min **Retention time:** *p*CS: 1.4–1.9 min *p*TS: 0.8–1.2 min **Total run time:** 7 min **Study authors’ sample extraction procedure:** 1. Centrifuge blood at 3000× *g* at 4 °C for 10 min to obtain plasma; 2. Internal standard was added to the sample (10 µL); 3. Mix 30 µL of sample with 90 µL methanol; 4. Incubate for 20 min at room temperature; 5. Centrifuge at 4000× *g* and 4 °C for 10 min; 6. Collect and dry supernatant; 7. Reconstitute in the mobile phase.	**Mass spectrometry: triple quadrupole (Agilent)** **Mode of ionization**: negative mode **Ion source conditions**: ESI Gas temperature +300 °C Gas flow: 9 L/min, Nebulizer gas pressure: 35 psi Capillary voltage: 1500 V. **Scanning mode**: MRM ***m*/*z* transitions**: *p*TS: 171 → 80, 107.1 CE: 28 and 16 V Fragmentor: 106 V *p*CS: 187 → 80, 107 CE: 20 and 16 V Fragmentor: 94 V	**Validation criteria guideline(s):** Not specified **LLOQ (μg/mL)**: ^(b)^ **LLOD (μg/mL):** ^(b)^ **Calibration range(s):** Linearity range (μg/mL): *p*CS: 0–320 μg/mL R^2^ = 0.997 ± 0.002 **Intra- and inter-day precision (N = 4 preparations):** Mean ± 1 standard deviation, C.V.% n1: 0.352 ± 0.006, 1.83 (intra) and 0.342 ± 0.015, 4.28 (inter) n2: 0.435 ± 0.001, 0.21 and 0.441 ± 0.007, 1.63 n3: 0.486 ± 0.022, 4.57 and 0.501 ± 0.033, 6.67 n4: 0.521 ± 0.002, 0.32 and 0.606 ± 0.025, 4.16 **Stability:** Criteria: Plasma samples: −80 °C for more than 3 months (data not provided) Processed samples: room temperature for 24 h (CV = 9%; accuracy data not provided) **Recovery %:** Concentrations tested: 20, 40, 80, 160, 320 μg/mL “The theoretical concentrations were compared with the measured concentration values, and the percentage recoveries were calculated for each spiked concentration”. 20: 100.2% 40: 102.0% 80: 100.4% 160: 107.1% 320: 104.0% **Storage conditions:** Samples solutions storage: −80 °C Stock storage: −20 °C	Cuoghi et al. 2012 [38]
**IxS**	**Matrix:** Human plasma In addition to the following: 1. other plasma sources: goat, pig, rat, and diabetic nephropathy mouse model; 2. ciPTEC-OAT1 lysate. **Internal standard**: isotope-labeled IxS potassium salt [^13^C_6_]-IxS, 0.5 μg/mL. **Column**: Polaris 3 C18-A column, 50 × 2 mm, dp = 3 μm, average pore diameter = 10 nm (Varian, Middelburg, The Netherlands). pre-column: 10 × 2 mm **Temperature**: Column: 40 °C Sample rack: 4 °C **Mobile phase**: A: 0.1% (*v*/*v*) formic acid in water B: ACN **Gradient**: 0.0–1.5 min: 15 → 35%B 1.5–2.0 min: 100% B 2.0–3.0 min: 15% B **Injection volume**: 10 µL **Flow rate**: 0.5 mL/min **Total run time**: 3 min **Retention time**: 1.3–1.38 min (In all matrices) **Study authors’ sample extraction procedure:** 1. Mix 20 µL of plasma with 80 μL of ACN containing 0.5 μg/mL of internal standard; 2. Vortex 2 min; 3. Centrifuge at 2 min at 10,000× *g*; 4. 64 μL of supernatant mixed with 200 µL of water in a polypropylene 96-deep well plate.	**Mass spectrometry: triple quadrupole (Thermo electron)** **Mode of ionization**: negative mode **Ion source conditions**: ESI Spray voltage: 5000 V Capillary temperature: 320 °C Skimmer voltage: off Collision gas: Ar at 1.4 mTorr **Scanning mode**: MRM ***m*/*z* transitions**: IxS: 212.0 → 80.0 and 132.0 CE: −24 V, −21 V [^13^C_6_]-IxS: 218.0 → 80.0 and 138.0 CE: −27 V, −21 V	**Validation criteria:** FDA ^(d)^ and EMA ^(f)^ **LLOQ (μg/mL)**: 0.1 **LLOD (μg/mL)**: 0.03 **Precision and accuracy** met the acceptance criteria ^(d, g)^ for all matrices. Human plasma: 10 and 50 μg/mL. Accuracy: 104.8% and 99.0% Within-day precision: 2.6% and 2% Between day precision: 4% and 3.3% Data for other matrices are available in the original manuscript. **Calibration range prepared in 4.2% BSA in** **water (μg/mL):** 0.1–100 R^2^ = 0.994 ± 0.002 **Stability:** Criteria (N = 4): Benchtop: 24 h at room temperature Freeze–thaw (3 cycles) Long-term stability: 2 months at −30 °C Human plasma at 2.5 and 75 μg/mL: Benchtop: 99.1 ± 2.3 and 99.8 ± 1.9% Freeze–thaw: 97.9 ± 3.4 and 99.1 ± 0.8% Long-term stability: 96.5 ± 0.6 and 97.5 ± 0.7% **Recovery and matrix effect (N = 6):** (a) Spiked plasma, then sample extraction; (b) Processed plasma, then spiked with analytes and extracted; (c) No treatment. **Matrix:** Human plasma: Recovery: 90% ± 3 (a:b ratio) Matrix effect (IxS): 110% ± 2 (b:c ratio) Matrix effect ([^13^C_6_]-IxS): 100% ± 3 (b:c ratio) **Storage**: −30 °C	Ahmed et al. 2022 [39]
**IxS** In addition to other analytes: CMPF	**Matrix**: Human plasma **Internal standards**: isotope-labeled IxS-d4, 5000 ng/mL **Column**: Waters Acquity BEH Shield RP18 column, dp = 1.7 μm, 2.1 × 50 mm. Pre-column: Waters Acquity BEHShield RP18 VanGuard (1.7 μm, 2.1 × 5 mm). **Temperature**: Column: 40 °C **Mobile phase**: A: 0.1% formic acid, 5% ACN, and 2 mM ammonium formate in water, B: 0.1% formic acid, 5% water, and 2 mM ammonium formate in ACN. **Gradient**: 0.50–4.50 min: 5 → 85% B 4.50–4.51 min: 85 → 95% B 4.51–6.50 min: 95% B 6.50–6.51 min: 95 → 5% B 6.51–8.50 min: 5% B **Injection volume**: 10 µL **Flow rate**: 0.3 mL/min **Retention time**: IxS: 2.3 min **Total run time:** 8.5 min **Study authors’ sample extraction procedure:** 1. Mix 20 µL sample with 280 µL phosphoric acid (4%), 50 µL internal standard, and 100 µL methanol (50%*v*/*v*); 2. Vortex; 3. Solid-phase extraction (SPE) with Oasis WAX μElution plate [pre-conditioned with 200 μL of methanol and water]: load 400 μL sample, wash with 200 μL water (2% formic acid), and elute using 50 μL of aqueous solution of 0.25% ammonia in methanol; 5. Dilute the extract with 50 μL water.	**Mass spectrometry: triple quadrupole (Shimadzu)** **Mode of ionization**: negative mode **Ion source conditions**: ESI Nebulizer flow rate: 3 L/min Drying flow rate 15 L/min Collision-induced dissociation gas pressure: 230 kPa De-solvation line temperature: 250 °C Heat block temperature: 400 °C. Collision gas: Ar. **Scanning mode**: MRM ***m*/*z* transitions**: IxS: 212.0 → 132.1 Dwell time: 0.10 s CE:21 V IxS-d4: 216.1 → 136.1 Dwell time: 0.10 s CE: 20 V	**Validation criteria guideline(s):** FDA ^(d)^ **LLOQ (μg/mL)**:0.05 (units in the original manuscript seem to be inconsistent) **LLOD (μg/mL)**: ^(b)^ **Linearity range (μg/mL):** 0.05–200 μg/mL, R^2^ ≥ 0.9997 **Precision and accuracy** met the acceptance criteria ^(d)^. QC (concentrations + endogenous), concentrations: 0.05, 0.15, 4.5, 150 μg/mL Within batch (endogenous, LLOQ, LQC, MQC, HQC): Sample 1: Accuracy (%): –, 93.3, 92.5, 107.3, and 103.5 Precision (%CV): 8.1, 8.2, 4.4, 1.1, and 2.5 Sample 2: Accuracy (%): –, 97.9, 98.2, 101.0, and 96.2 Precision (%CV): 5.2, 6.4, 5.2, 3.9, and 3.5 Sample 3: Accuracy (%): –, 100.8, 102.9, 106.2, and 99.7 Precision (%CV): 3.9, 8.7, 2.9, 2.5, and 3.4 Batch-to-batch (endogenous, LLOQ, LQC, MQC, HQC): Accuracy (%): –, 97.3, 97.9, 104.9, and 99.8 Precision (%CV): 9.7, 9.3, 6.0, 4.1, and 4.3 **Specificity:** Tested at LLOQ: no interference was reported. **Stability:** Autosampler stability at MQC and HQC: 24 h at 20 °C. Accuracy: 91.2–103.4% Freeze–thaw cycles at MQC and HQC: 3 cycles. Accuracy: 97.0–104.7% **Carryover:** IxS: less than 0.04% **Recovery at LQC, MQC, and HQC**: mean (range) Processed QCs vs. extracted plasma spiked with QCs (as a percentage) IxS recovery rate: 80.6 (68.6–90.5), 62.5 (46.4–102.6), and 84.7 (47.8–102.8) % IxS mean recovery rate corrected by the internal standard: 100.7 (95.9–107.8), 101.9 (98.8–107.2), and 101.6 (99.7–108.8) **Matrix effect at LQC, MQC, and HQC:** Extracted plasma spiked with QCs vs. liquid chromatography diluents spiked with QCs (as a percentage) IxS mean (range) matrix effect: 103.8 (96.9–110.6), 100.0 (93.6–104.3), and 103.5 (99.1–107.5) % IxS mean (range) matrix effect corrected by the internal standard: 101.9 (94.6–108.5), 105.5 (104.1–107.4), and 101.1 (97.2–104.5)% **Storage:** −80 °C	Oda et al. 2022 [51]
**IxS** ***p*CS** In addition to other analytes: catechol sulfate, guaiacol sulfate, phenyl sulfate.	**Matrix**: Human plasma **Internal standards**: *p*- p-toluene sulfonic acid (PTSA), 10 μg/mL **Column**: Agilent eclipse plus C18 column (150 × 4.6 mm; 5 μm particle size), Agilent Technologies (Santa Clara, California, USA) Pre-column: Agilent ZORBAX HILIC PLUS column (100 × 4.6 mm; 3.5 μm particle size), Agilent Technologies (Santa Clara, California, USA) **Temperature**: column: 25 °C, autosampler: 10 °C **Mobile phase**: A: water with 5 mM ammonium acetate. B: methanol **Gradient**: 0.00–2.00 min: 20% → 60%B 2.00–5.00 min: 60% → 90%B 5.00–11.00 min: 90%B 11.00–12.00 min: 90% → 20%B 12.00–15.00 min: 20%B **Injection volume**: 5 µL **Flow rate:** 0.6 mL/min **Retention time**: IxS: 6.26 min *p*CS: 6.84 min **Total run time:** 15 min **Study authors’ sample extraction procedure:** 1. Thaw samples on ice and vortex (1 min); 2. Mix 200 µL plasma sample with 170 µL of ice-cold ACN/0.1% formic acid; 3. Add 30 µL of internal standard (10 µg/mL in ACN); 4. Vortex (5 min); 5. Centrifuge at 8000 rpm for 30 min; 6. Evaporate to dryness (4–6) h under speed vac; 8. Reconstitute with 200 µL ACN in water (1:1 *v*/*v*).	**Mass spectrometry: triple-quadrupole (Agilent)** **Mode of ionization**: Negative ion mode **Ion source conditions**: ESI capillary voltage: 4000 V Gas temperature: 2800 C Drying gas flow: 45 L/min Nebulizer gas flow: 8 L/min Capillary current: 4400 nA. **Scanning mode**: MRM ***m*/*z* transitions**: IxS: 212 → 132, CE: 15 eV, Fragmentor: 100, Dwell time: 200 (unit not provided) *p*CS: 187 → 107, CE: 22 eV, Fragmentor: 100, Dwell time: 200 PTSA: 171 → 80, CE: 25 eV, Fragmentor: 75, Dwell time: 200	**Validation criteria guideline(s):** ICH M10 bioanalytical method validation guideline ^(h)^. **LLOQ (μg/mL)**: IxS: 0.05 *p*CS: 0.05 **LLOD (μg/mL):** IxS: 0.02 *p*CS 0.02 **Linearity range (μg/mL):** 0.05–10, R^2^ ≥ 0.997 **QCs concentrations (μg/mL):** LQC: 0.05 μg/mL MQC: 0.2 μg/mL HQC: 1 μg/mL ULOQ: 10 μg/mL **Precision and accuracy**: Tested with LQC, MQC, and HQC concentrations (N = 6). Accuracy (mean ± SD), Precision (%CV): IxS intra-day: LQC: 0.049 ± 0.004 μg/mL, 8.470% MQC: 0.232 ± 0.0061 μg/mL, 5.534% HQC: 1.175 ± 0.014 μg/mL, 3.706% *p*CS intra-day: LQC: 0.047 ± 0.008 μg/mL, 0.183% MQC: 0.228 ± 0.0038 μg/mL, 3.601% HQC: 1.093 ± 0.031 μg/mL, 7.821% IxS inter-day: LQC: 0.042 ± 0.0036 μg/mL, 6.864% MQC: 0.204 ± 0.0077 μg/mL, 7.505% HQC: 1.188 ± 0.013 μg/mL, 3.466% *p*CS inter-day: LQC: 0.049 ± 0.0017 μg/mL, 3.638% MQC: 0.195 ± 0.001 μg/mL, 1.631% HQC: 1.095 ± 0.041 μg/mL, 10.632% **Recovery**: Processed QCs vs. solvents spiked with QCs (as a percentage) Tested with LQC, MQC, and HQC concentrations. IxS intra-day: LQC: 97.7% MQC: 116.3% HQC: 117.2% *p*CS intra-day: LQC: 94.1% MQC: 114.2% HQC: 109.3% IxS inter-day: LQC: 85.7% MQC: 101.9% HQC: 118.7% *p*CS inter-day: LQC: 99.3% MQC: 97.8% HQC: 109.5% **Selectivity:** By testing the appearance of peaks at the retention times of the analytes when the blank matrix is injected. No peaks appeared. **Specificity:** tested for analytes at 1 μg/mL with an internal standard of 10 μg/mL. “Chromatograms obtained in the specificity experiment showed clear separation of all the uremic toxins with good resolution”. **Carryover:** tested with blank sample after LQC, MQC, HQC, and ULOQ. No carryover was reported. **Storage: −**80 °C	Ragi, N. et al. 2023 [52]
**IxS** In addition to other analytes in plasma: AnA, KA, Kn, IAA, nicotinic acid, serotonin, Trp. In addition to other analytes in gastric juices: AnA, KA, Kn IAA, Nicotinic acid, Trp.	**Matrix**: IxS only in plasma (serum also mentioned in the original text) **Internal standards**: Tryptophan-d3 (Trp-d3; negative 20 μM) **Column**: Atlantis T3 2.1 × 150 mm, 3 μm (Waters, Milford, MA, USA). **Temperature(s):** Column: 35 °C Autosampler: 4 °C **Mobile phase**: A: 0.1% formic acid in 95% water B: 0.1% formic acid in 95% methanol **Gradient**: 0.0–0.5 min: 5% B 0.5–5.0 min: 5 → 15% B 5.0–8.0 min: 15 → 50% B 8.0–14.0 min: 50 → 90% B 14.0–15.0: 90% B 15.0–22.0: 5% B **Injection volume**: 5 µL **Flow rate**: 0.2 mL/min **Retention time**: IxS: 13.5 min Trp-d3: 11.3 min **Total run time:** 22 min **Study authors’ sample extraction procedure:** Plasma samples: 1. Mix 50 µL plasma with 10 µL of internal standard; 2. Add 400 μL of ACN and 20 μL of 0.1% formic acid in water; 3. Vortex 30 s and centrifuge at 20,817× *g* for 10 min (4 °C); 4. Collect 400 µL of the supernatant and evaporate under N_2_; 5. Reconstitute in 100 μL of 0.1% formic acid in water.	**Mass spectrometry: triple quadrupole (AB Sciex)** **Mode of ionization**: negative ionization mode **Ion source conditions**: Ion Spray Polarity-switch pause time: 700 ms Curtain gas pressure: 35 psi Source temperature: 550 °C Gas source 1: 55 psi Gas source 2: 45 psi Ion spray voltage: +5500 V and −4500 V **Scanning mode**: MRM ***m*/*z* transitions**: IxS: 211.8 → 79.9 Dwell time: 85 ms “DP” (not defined): −35 V “EP” (not defined): −7 V “CEP” (not defined): −12 V “CE” (not defined): −32 V “CXP” (not defined): 0 V Trp-d3: 205.9 → 115.9 Dwell time: 85 ms “DP”: −40 V “EP”: −10 V “CEP”: −10 V “CE”: −24 V “CXP”: 0 V	**Validation criteria guideline(s):** FDA ^(d)^ **LLOQ (μg/mL):** 0.021 **LLOD (μg/mL):** ^(b)^ **Linearity range (μg/mL):** IxS: 0.021–4.264, R^2^ = 0.9979 **QCs [LLOQ (μM) Low (μM) Medium (μM) High (μM)]:** IxS: 0.1, 0.25, 2.5, 15 **In μg/mL:** 0.0213, 0.053, 0.533, and 3.198 **Precision and accuracy** met the acceptance criteria ^(d)^. Accuracy (%): LLOQ, low, medium, and high QC Inter-day (N = 5): 104, 101, 103, and 103 Intra-day (N = 8): 98.1, 95.1, 104, and 97.0 Precision (CV, %): LLOQ, low, medium, and high QC Inter-day (N = 5): 8.59, 6.19, 7.15, and 4.84 Intra-day (N = 8): 6.20, 8.80, 3.40, and 3.00 **Stability** Freeze–thaw stability: 3 cycles Benchtop stability: 3 h at room temperature Long-term stability: 2, 4, 6 weeks at–80 °C Post-operative stability: 12 and 24 h at 4 °C in the autosampler Processed stability (before reconstitution): 24 h at 4 °C In plasma (IxS) (LQC, MQC, and HQC): Freeze–thaw stability %: Cycle 1: 115%, 102%, and 103% Cycle 2: 108%, 104%, and 98.4% Cycle 3: 117%, 114%, and 109% Benchtop stability (%): 97.3%, 94.1%, and 102% Long-term stability (%): 2 weeks: 106%, 91.3%, and 100% 4 weeks: 102%, 102%, and 103% 6 weeks: 115%, 134%, and 133% Autosampler stability (%): Post-operative stability: 12 h: 103%, 103%, and 99.1% 24 h: 102%, 113%, and 112% Processed stability: 24 h: 97%, 106%, and 101% **Recovery/Matrix effect:** Recovery: the ratios of analyte peak areas when spiked into a matrix and extracted to analytes spiked into the extracted matrix. IxS: 82.1%, 85.2%, and 83.6% for the low, medium, and high QCs, respectively. Matrix effect: the ratios of analyte peak areas when spiked into the extracted matrix to analytes spiked to neat solution. IxS: 92.6%, 95.8%, and 93.3% for the low, medium, and high QCs, respectively. **Storage:** stock solutions: −20 °C stability: −80 °C	Choi et al. 2016 [53]
**IxS,** ***p*CS,** In addition to: dihydroepiandrosterone sulphate, ethyl-β-D-glucuronide, ethyl sulfate, estrone sulfate resveratrol, L-tartaric acid.	**Matrix:** serum, plasma, and urine **Details are mentioned in the serum section.**	**Details are mentioned in the serum section.**	**Details are mentioned in the serum section.**	Monosık, R. et. al. 2016 [49]
**IxS** ***p*CS** In addition to other analytes: Creatinine, 4-ES, 1-methyladenosine, PS, TMAO.	**Matrix**: Human plasma/serum **Details are mentioned in the serum section.**	**Details are mentioned in the serum section.**	**Details are mentioned in the serum section.**	Kanemitsu et al. 2017 [50]
**OTHER MATRICES**				
***p*CS** In addition to other analytes: HA, PAG	**Matrix**: Human urine **Internal standards**: ^2^H_3_-4-cresol sulfate (30 ng/mL) **Column**: Waters Acquity UPLC BEH C18 column (1.7 μm, 2.1 × 100 mm) **Temperature**: Column: 60 °C **Mobile phase**: A: 0.1% formic acid in water B: 0.1% formic acid in ACN **Gradient**: 0:00–1:30 min: 5% B 1:30–2:20 min: 5 → 95% B 95%--> 5% B (duration is not clear) **Injection volume**: 1 µL **Flow rate**: 0.6 mL/min **Retention time**: *p*CS: 1.63–1.64 min **Total run time:** 5 min [to include both positive mode and negative mode runs, 2 injections] **Study authors’ sample extraction procedure:** **Sample extraction**: 1. Collect urine into standard 1-L plastic jars with boric acid; 2. Store aliquots at –20 °C; 3. Specimens were thawed; 4. Dilute the sample 1:1000 in an analytical well plates; 4. 10 µL of each internal standard was added to each well.	**Mass spectrometry: triple quadrupole (Waters)** **Mode of ionization**: *p*CS: negative mode. **Ion source conditions**: ESI Capillary voltage: 3000 V Extractor voltage: 3 V Source temperature: 150 °C De-solvation temperature: 600 °C Cone gas flow: off De-solvation gas flow: 800 L/hour Collision gas flow: 0.15 mL/min **Scanning mode**: MRM ***m*/*z* transitions**: *p*CS: 187 → 106 Cone voltage (CV) = 34 V Collision energy (CE): 22 eV ^2^H_3_-4-cresol sulfate: 190 → 110 Cone voltage (CV): 34 Collision energy (CE): 22 eV	**Validation criteria guideline(s):** FDA ^(d)^ **LLOQ (μg/mL)**: 0.001 **LLOD (μg/mL)**: ^(b)^ **Linearity range (μg/mL):** *p*CS: 0.001–1 [prepared in water and not in urine] R^2^ > 0.997 **Quality control (QCs) concentrations (μg/mL):** Low: 0.003, medium: 0.030, high: 0.300 **Precision and accuracy:** 6 replicates of QCs. **Intra-assay:** 3.3 (0.1), 28.8 (2.2), and 316.4 (11.1) Precision (CV) for low, medium, and high QC, %: 3.0, 7.6, and 3.5 Accuracy for low, medium, and high QC, %: 110.0, 96.0, and 105.5 **Inter-assay:** N = 18. Precision for low, medium, and high QC, %: 16.7, 8.6, and 5.5 Accuracy for low, medium, and high QC, %: 100.0, 104.0, and 105.3 **Stability:** ^(b)^ **Matrix effect:** By serial dilution of real human urine samples and comparing for back-calculated concentrations with the dilution factors (no difference concentrations noted). Assay recovery: not provided **Carryover:** No significant interfering peaks were observed in blank samples (after each batch run: not clear in the original paper if conducted per standard protocol of following a high QC).	Wijeyesekera et al. 2012 [54]
**IxS** In addition to other analytes Inosine, IAA, Methyl guanidine, N-acetyl arginine, XA.	**Matrix**: Urine. **Internal standards**: C6-indoxyl sulfate **Column**: reverse phase column 1.6 μm Polar C18 (100 × 1.0 mm) (Phenomenex, Luna^®^ Omega) **Temperature**: Column: 40 °C **Mobile phase**: A: 0.1% formic acid in water B: ACN **Gradient**: 0.0–1.0 min: 5% B 1.0–3.5 min: 55% B 3.5–5.0 min: 95% B 5.0–7.5 min: 5% B and re-equilibration **Injection volume**: 5 µL **Flow rate**: 0.15 mL/min **Retention time**: IxS: 2.79 min **Total run time:** 8.5 min **Study authors’ sample extraction procedure:** 1. Centrifuge urine samples at 30,000× *g* for 10 min; 2. Internal standard added to 50 μL of supernatant; 3. Mixture diluted to 150 μL with water.	**Mass spectrometry: triple-quadrupole (Waters)** **Mode of ionization**: negative for IxS **Ion source conditions**: ESI Capillary voltage: 2000 V De-solvation gas flow: 650 L/h De-solvation temperature: 350 °C Source temperature: 150 °C **Scanning mode**: MRM ***m*/*z* transitions**: IxS: 212.00 → 132.02, CV: 30 V, CE: 20 eV. IxS-C6: 217.85 → 137.99, CV: 30 V, CE: 25 eV.	**Validation criteria guideline(s):** **LLOQ (μg/mL)**: 0.480 **LLOD (μg/mL)**: 0.0158 **Linearity range (μg/mL):** IxS: 0.480 to 48 R^2^ = 0.99977 **QC concentrations (μg/mL):** Low: 0.1, medium: 1, and high: 5 **Intra-day precision (RSD%) and accuracy (%) (N = 5):** 5 Replicates in one day for low, medium, and high QCs. RSD = 9.3%; accuracy = 91.8% RSD = 2.8%; accuracy = 97.8% RSD = 0.8%; accuracy = 99.1% **Inter-day precision (RSD%) and accuracy (%) (N = 12):** 3 replicates in 4 days for low, medium, and high QCs. RSD = 13.2%; accuracy = 90.3% RSD = 5.1%; accuracy = 98.0% RSD = 6.1%; accuracy = 94.0% **Stability, 4 QCs (0, low, medium, and high):** Autosampler stability (change %): 24 h at 10 °C −0.15%, −0.74%, 1.41%, and −0.36%. Freeze–thaw stability (change %): 3 cycles at −20 °C at 24 h storage 20.10%, 19.47%, 17.08%, and 18.68% **Recovery (%):** The preparation methods tested are as follows: SPE: OASIS PRIME HLB 30 mg and Oasis MCX 30 mg. Dilution: 1:2 Comparing the concentrations of the analytes in urine QCs (low, medium, and high) to the concentrations obtained from samples prepared in the initial mobile phase solution. SPE (OASIS HLB): 81.8% SPE (Oasis MCX): 7.9% Dilution (1:2): 108.7% Dilution provided better recoveries and was used for later analysis. **Matrix effect:** Comparing the internal standard intensity in urine to the same concentrations prepared in standard solutions. IxS: 17% **Storage**: −20 °C	Olesova et al. 2020 [55]
**IxS** In addition to other analytes: AnA, 3-hydroxykynurenine, hydroxy anthranilic acid, 5-hydroxy-indole-3-acetic acid, IAA, indole-3-acetyl-glycine indole-3-lactic acid, indole-3-propionic acid, KA, Kn, melatonin nicotinamide, nicotinic acid, quinolinic acid, serotonin, tryptamine, Trp, XA.	**Matrix**: Human serum, human urine, cell culture **Details are mentioned in the serum section.**	**Details are mentioned in the serum section.**	**Details are mentioned in the serum section.**	Zhu et al. 2011 [32]
**IS,** ***p*CS,** In addition to: dihydroepiandrosterone sulphate, ethyl-β-D-glucuronide, ethyl sulfate, estrone sulfate resveratrol, L-tartaric acid.	**Matrix:** serum, plasma, and urine **Details are mentioned in the serum section.**	**Details are mentioned in the serum section.**	**Details are mentioned in the serum section.**	Monosık, R. et. al. 2016 [49]
**IxS** ***p*CS**	**Matrix**: Human saliva **Internal standards**: isotope-labeled IxS-d4, *p*CS-d7 (stock 1 mg/mL) **Column**: Kinetex C-18 column (100 mm, 4.6 mm, particle size 2.6 mm) (Phenomenex, Torrance, CA, USA). **Temperature(s)**: Column: 40 °C **Mobile phase**: A: formic acid 0.1% in water B: formic acid 0.1% in methanol **Gradient**: 0.00–0.50 min: 10% B 0.50–4.50 min: 10 → 95% B 4.50–8.50 min: 95% B **Injection volume**: 10 µL **Flow rate**: 0.5 mL/min **Retention time**: IxS: 6.29 min *p*CS: 6.98 min **Total run time:** ^(b)^ **Study authors’ sample extraction procedure:** Blank samples were obtained by incubating saliva with activated charcoal for 30 min. Samples were collected using cotton swabs. 1. Centrifuge the cotton swabs at 945× *g* at 20 °C for 3 min; 2. Store the saliva at–80 °C before analysis; 3. Add ACN 1:4, *v*/*v*; 4. Incubate at −20 °C for 20 min; 5. Centrifuge at 9300× *g* and 4 °C for 10 min; 6. Use the supernatant for the assay.	**Mass spectrometry: hybrid triple quadrupole/linear ion trap (AB Sciex)** **Mode of ionization**: **Ion source conditions**: ESI Curtain gas: N_2_, 241 kPa Ion source gas 1: N_2_, 413 kPa Ion source gas 2: N_2_, 275 kPa Ion spray voltage: 4500 V Ion source temperature: 600 °C **Scanning mode**: MRM ***m*/*z* transitions**: IxS: 211.9 → 79.8 Declustering potential: −60 V CE: −38 V Entrance potential: −10 V Collision exit potential: −5 V IxS-d4: 216.0 → 79.9 Declustering potential: −65 V CE: −28 V Entrance potential: −10 V Collision exit potential: −7 V *p*CS: 186.9 → 106.9 Declustering potential: −65 V CE: −28 V Entrance potential: −10 V Collision exit potential: −7 V *p*CS-d7: 194.0 → 114.0 Declustering potential (DP): −60 V CE: −30 V Entrance potential: −10 V Collision exit potential: −7 V	**Validation criteria guideline(s):** EMA ^(f)^ and FDA ^(d)^ **LLOQ (μg/mL)**: IxS: 0.0015 *p*CS: 0.0015 **LLOD (μg/mL)**: ^(b)^ **Linearity range (μg/mL):** IxS: 0.0015–0.2, R^2^ = 0.999 *p*CS: 0.0015–0.5, R^2^ = 0.999 **QCs concentrations:** IxS: 1.5, 2, 80, and 150 ng/mL *p*CS: 1.5, 4.5, 250, and 500 ng/mL **Within-run (N = 5) and between run (N = 20) precision and accuracy** IxS: Within-run precision (%): 3.2–6.4, 2.6–6.5, 2.2–5.5, and 2.2–5.8 Within-run accuracy (%): 110–114, 87–92, 98–103, and 101–105 Between-run precision (%): 4.8, 4.7, 4.2, and 4.3 Between-run accuracy (%):113, 90, 102, and 103 *p*CS: Within-run precision (%):1.7–2.7, 0.9–4.7, 0.5–4.1, and 2.2–6.9 Within-run accuracy (%):103–118, 85–88, 91–102, and 97–105 Between-run precision (%):3.4, 3.5, 3.3, and 4.7 Between-run accuracy (%):108, 85, 92, and 103 **Stability** Performed for low and high QCs. Expressed as stability% (calculation method not defined in the manuscript) and accuracy% - freeze–thaw stability (3 cycles, N = 10). IxS: low QC (109 and 99)%, high QC (103 and 108)% *p*CS: low QC (114 and 99)%, high QC (105 and 109)% - short-term stability (4 h at room temperature, N = 10). IxS: low QC (95 and 86)%, high QC (110 and 98)% *p*CS: low QC (110 and 96)%, high QC (100 and 104)% - long-term stability (30 days at −20 °C, N = 30). IxS: low QC (99–103 and 90–103)%, high QC (92–115 and 96–114)% *p*CS: low QC (111 and 96–97)%, high QC (85–102 and 88–106)% - autosampler stability (24 h, N = 10): IxS: low QC (98 and 89)%, high QC (99 and 104)% *p*CS: low QC (98 and 85)%, high (96 and 99)% - autosampler stability (48 h, N = 10): IxS: low QC (98 and 89)%, high QC (101 and 106) *p*CS: low QC (99 and 86)%, high QC (101 and 105) - dilution integrity, n = 5 (dilution factor not provided). IxS: RSD 4.6% and accuracy 105% *p*CS: RSD 1.7% and accuracy 90% **Matrix effect:** The difference between standard solution and post-extraction spiked samples. Absolute matrix effect for IxS and the internal standard: Low concentration: 99% and 99% Medium concentration: 93% and 92% High concentration: 95% and 95% Absolute matrix effect for *p*CS and the internal standard: Low concentration: 100% and 99% Medium concentration: 103% and 101% High concentration: 99% and 96% **Storage:** Storage (long-term stability): −20 °C stock solutions, QCs samples, and calibration standards: −26 °C	Giebułtowicz et al. 2016 [56]
**IxS** ***p*CS** **In addition to:** CMPF, HA, 3-IAA, KA, Kn, *p*CG, PAG, TMAO, Tyr, Phenylalanine, Trp.	**Matrix**: saliva **According to the original manuscript, the analytical method was similar to their previously published assay [31]. It was discussed earlier in the serum section.** The following details were provided. **Column**: Accucore^TM^ PFP column (100 × 2.1 mm, 2.6 μm, Thermo Fisher Scientific). **Temperature**: 40 °C **Mobile phase**: A: 0.1% formic acid in water. B: ACN. **Gradient**: 0.0–1.0 min: 1% B 1.0–6.5 min: 65% B 6.6–8.0 min: 90% B 8.1 min: 1% B **Flow rate**: 0.5 mL/min **Retention time**: IxS: 5.75 min *p*CS: 6.18 min **Study authors’ sample extraction procedure:** Unstimulated saliva was collected (1 mL) into a plastic tube and frozen at −80 °C. 1. Mix 50 µL of saliva with 25 µL of the internal standard mixture and methanol (340 µL); 2. Homogenize and centrifuge at 9000× *g* and 4 °C for 10 min; 3. completely dry the supernatant; 4. Reconstitute the residue with water (80 µL).	**According to the original manuscript, the analytical method was similar to their previously published assay [31]. It was discussed earlier in the serum section.** The following details were mentioned. **Mass spectrometry: triple-quadrupole (Thermo)** **Mode of ionization**: Negative mode for IxS and *p*CS. **Ion source conditions**: ESI **Scanning mode**: MRM ***m*/*z* transitions:** IxS: 212.1 → 80.0 and 81.0 Collision energy: 23 V and 17 V *p*CS: 187.1 → 107.1 V and 105.1 V Collision energy: 21 V and 34 V	**Validation criteria guideline(s):** **LLOQ (μg/mL)**: IxS: 0.001 μg/mL *p*CS: 0.001 μg/mL **LLOD (μg/mL)**: IxS: 0.0003 μg/mL *p*CS: 0.0003 μg/mL **Linearity range (μg/mL):** IxS: 0.001–50 μg/mL, R^2^= 0.9993 ± 0.0006 *p*CS: 0.001–50 μg/mL, R^2^= 0.9995 ± 0.0004 **QCs concentrations:** 30, 150, 8000, 40,000 ng/mL **Accuracy and precision (N = 6) on 3 different days:** **Intra-day for 30, 150, 8000, and 40,000 ng/mL, respectively:** Accuracy (Bias %): IxS: 3.3%, 6.7%, −1.7%, and 6.7%, respectively *p*CS: 10.0%, 0.0%, 2.4%, and 1.9%, respectively Precision (CV%): IxS: 8.8%, 4.3%, 10.1%, and 7.6%, respectively *p*CS: 7.8%, 4.9%, 7.0%, and 8.1%, respectively **Inter-day for 30, 150, 8000, and 40,000 ng/mL, respectively:** Accuracy IxS: 1.3%, 3.3%, −5.1%, and −2.2%, respectively *p*CS: 10.0%, −1.3%, −2.2%, and −1.8%, respectively Precision IxS: 3.3%, 5.9%, 7.0%, and 10.9%, respectively *p*CS: 4.4%, 6.6%, 8.0%, and 6.4%, respectively **Carryover:** Chromatography of blank injection after 3 injections of high concentration (not determined) had no peaks (no carryover) **Stability** (numerical data not provided in the original manuscript)**:** 20 °C: **not stable** for 5 h 4 °C: stable up to 24 h −20 °C: stable for up to three months Freeze–thaw: stable up to 3 cycles **Matrix effect% with (CV%), N = 6, for 100 and 1000 ng/mL, respectively:** By comparing the peak area of internal standards prepared in saliva to the peak area of the same concentrations prepared in water. IxS: +4 (11%) and −7 (13%), respectively *p*CS: +4 (13%) and −2 (11%), respectively **Storage**: Saliva (not processed): −80 °C Stability storage: −20 or −80 °C (not specified)	Fabresse N, et al. 2023 [57]
***p*** **CS** **In addition to other 50 salivary metabolites**	**Matrix**: saliva **Internal standards:** L-phenylalanine-^13^C_6_ or L-leucine-^13^C_6_ **Column**: Hypersil GOLD™ VANQUISH™ C18 UHPLC column (2.1 mm × 150 mm, 1.9 µm; Thermo scientific) **Temperature(s)**: 45 °C **Mobile phase**: A: 0.1% formic acid in water B: 0.1% formic acid in methanol **Gradient**: 0.0–1.0 min: 0.0%B 1.0–8.0 min: 0.0–50.0%B 8.0–10.0 min: 50.0–100.0%B 10.0–12.0 min: 100.0%B 12.0–12.1 min: 100.0–0.0%B 12.1–16.0 min: 0.0%B **Injection volume**: 5 μL **Flow rate**: 0.3 mL/min **Retention time**: 7.85 min **Total run time:** 16 min **Study authors’ sample extraction procedure:** Unstimulated saliva: 1. Collected in a 50 mL Falcon^®^ polypropylene tube; 2. Collect at least 3 mL of saliva; 3. Centrifuge at 12,000 rpm and 4 °C for 5 min. Stimulated saliva: 1. Collect the sample by chewing salivettes (Sarstedt Co. Ltd., Nu¨mbrecht, Germany) for 2 min; 2. Centrifuged at 12,000 rpm and 4 °C for 5 min; Supernatants are extracted or stored at –80 °C. Sample preparation/extraction: 1. Mix 100 µL of the sample with 400 µL of ACN (1:4); 2. Vortex (2 min); 3. Centrifuge at 12,000 rpm and 4 °C for 15 min; 4. Supernatant is completely evaporated using N_2_; 5. reconstitute with water (volume not specified).	**Mass spectrometry: QTOF (AB Sciex)** **Mode of ionization**: negative **Ion source conditions**: ESI **Scanning mode**: MRM Ion spray voltage: −4500 V Temperature: 500 °C, Curtain gas: 30 psi, De-clustering potential: ±90 V, Collision energy: −20 V. Drying gas: N_2_ Nebulizing gas: N_2_ Collision gas: N_2_ ***m*/*z* transitions**: *p*CS: 187.01 → 79.9665 CE: −20	**Validation criteria guideline(s):** ***The following tests were applied using artificial saliva.*** **LLOQ (μg/mL)**: *p*CS: 0.02 μM = 0.003764 μg/mL **LLOD (μg/mL)**: *p*CS: 0.01 μM = 0.001882 μg/mL **Linearity range (μg/mL):** *p*CS: 0.1–5 μM = 0.0188–0.9410 μg/mL, R^2^ = 0.9988 **QCs concentrations:** Low QC: 0.5 μM = 0.0941 μg/mL Middle QC: 2 μM = 0.3764 μg/mL High QC: 4 μM = 0.7528 μg/mL **Accuracy (error, %) and precision (RSD, %):** Intra-day (N = 5) for low-, middle-, and high-quality control concentration: Accuracy: 4.88%, 1.90%, and 0.16% Precision: 5.75%, 3.78%, and 3.62% Inter-day (N = 5) for low-, middle-, and high-quality control concentration: Accuracy: 0.32%, 3.42%, and −3.83% Precision: 2.72%, 3.50%, and 6.64% **Extraction solvent:** Saliva/ACN (1:4): lowest intensity for large molecules Saliva/Methanol (1:4): highest intensity for large molecules ACN: the best in protein precipitation and cleaning the sample (more suitable for salivary extraction)	Nam M, et al. 2023 [58]

Abbreviation list: ACN: acetonitrile. AnA: anthranilic acid. Ar: Argon. BSA: bovine serum albumin. CMPF: 3-carboxy-4-methyl-5-propyl-2-furan propionate. CV%: coefficient of variation percentage. DHCT: hydrochlorothiazide. dp: diameter of particles. EPA: United States Environment Protection Agency. ESI: electrospray ionization. FDA: Food and Drug Administration. HA: hippuric acid. HD: hemodialysis patients. IA: indoxyl acetate. IAA-d5: isotope-labeled indole-3-acetic acid. IAA: indole-3-acetic acid. IxS-d4: isotope-labeled indoxyl sulfate-d4. IxS: indoxyl sulfate. KA: kynurenic acid. Kn: kynurenine. LLOD: lower limit of detection. LLOQ: lower limit of quantification. MQwater: milliQ water. MRM: multiple reaction monitoring. MWCO: molecular weight cut-off. N_2_: Nitrogen gas. PAG: phenylacetylglutamine. *p*CG: *p*-cresyl glucuronide. *p*CS-d7: isotope-labeled *p*-cresyl sulfate-d7. *p*CS: *p*-cresyl sulfate. PG: phenyl glucuronide. Phe: phenylalanine. PS: phenyl sulfate. QC: quality control. R^2^: regression factor. S/N: signal-to-noise ratio. SPE: solid phase extraction. TMAO: Trimethylamine N-oxide. Trp: tryptophan. Tyr: tyrosine. UA-d5: isotope-labeled hippuric acid-d5. UA: uric acid. XA: xanthurenic acid. ^(a)^: the United States Environment Protection Agency (EPA): https://www.epa.gov/guidance. ^(b)^: Not reported in the original manuscript. ^(c)^: NCCLS EP5-T guidelines: https://www.document-center.com/standards/show/NCCLS-EP5. ^(d)^: FDA: Food and Drug Administration, https://www.fda.gov/regulatory-information/search-fda-guidance-documents/bioanalytical-method-validation-guidance-industry. ^(e)^: CLIA guideline: https://www.cdc.gov/clia/index.html. ^(f)^: EMA: European Medicine Agency. Bioanalytical method validation–Scientific guideline: https://www.ema.europa.eu/en/bioanalytical-method-validation-scientific-guideline. ^(g)^: SWGTOX: Scientific Working Group for Forensic Toxicology: https://doi.org/10.1093/jat/bkt054. ^(h)^: ICH: International Council of Harmonization: https://www.ich.org/products/guidelines.

**Table 2:** Associated biological applications for the validated LC-MS/MS assays for quantifying indoxyl sulfate and *p*-cresol sulfate.

SERUM	
**OBJECTIVE:** - To develop and validate a liquid chromatography-mass spectrometry (LC-MS/MS) assay for the quantification of seven uremic toxins in serum samples obtained from chronic kidney disease (CKD) patients (stages 1–5) and healthy volunteers. **STUDY DESIGN:** analysis of previously collected samples. **SAMPLE POPULATION (N = 78):** - Healthy volunteers (N = 19; 12 men) and CKD patients (N = 59; 32 men) - CKD stages were determined according to the estimated glomerular filtration rate (eGFR) CKD-1 patients (N = 10; 4 men) CKD-2 patients (N = 8; 2 men) CKD-3 patients (N = 8; 5 men) CKD-4 patients (N = 8; 6 men) CKD-5 non-dialysis (ND) patients (N = 6; 2 men) CKD-5 on dialysis (HD) (N = 19; 13 men) **AUTHORS’ KEY FINDINGS:** - **IxS concentrations at different CKD stages (μg/mL):** CKD-1: 0.5 ± 0.3 CKD-2: 0.7 ± 0.5 CKD-3: 1.7 ± 1.1 * (*p* < 0.01 vs. healthy control, 0.5 ± 0.3 µg/mL) CKD-4: 2.7 ± 2.1 * CKD-5 (ND): 4.6 ± 2.1 * CKD-5 (HD): 19.2 ± 9.9 * - ***p*CS concentrations at different CKD stages (μg/mL):** CKD-1: 9.5 ± 4.9 CKD-2: 10.3 ± 6.2 CKD-3: 20.0 ± 7.3 * (*p* < 0.01 vs. healthy control, 6.6 ± 3.7 µg/mL) CKD-4: 21.9 ± 13.7 * CKD-5 (ND): 26.5 ± 9.8 * CKD-5 (HD): 55.9 ± 15.1 * **ADDITIONAL PUBLISHED BIOLOGICAL APPLICATIONS:** - **Jongers B. et al., 2022 [74] cited this assay by Boeleart et al. [40] but in a non-targeted metabonomics analysis.**	Boelaert et al. 2013 [40]
**OBJECTIVE:** - To develop and validate an ultra-performance liquid chromatography-tandem mass spectrometry (UPLC-MS/MS) assay to simultaneously quantify 15 uremic toxins in patients with CKD stages 1–5 but not on dialysis (N = 488). **STUDY DESIGN:** Open-label, observational **SAMPLE POPULATION (N = 488):** - CKD patients not on dialysis at stages 1–5 (the study did not provide the data in each CKD stage). - CKD stages were determined using eGFR according to the Chronic Kidney Disease Epidemiology Collaboration (CKD-EPI) equation. **AUTHORS’ KEY FINDINGS:** - Serum concentrations (μM): IxS: 15.6 ± 17.1 (mean ± standard deviation (SD)), range <LLOQ–140.8, N < LLOQ = 14 equivalent in µg/mL to: 3.326 ± 3.65 [range <lower limit of quantification (LLOQ)–30.02] *p*CS: 75.7 ± 74.7, range <LLOQ–499.4, N < LOQ = 4 equivalent in µg/mL to: 14.246 ± 14.058 [<LLOQ–93.987] - Inverse correlations between eGFR and IxS (ρ −0.76, *p* < 0.0001) or *p*CS (ρ −0.68, *p* < 0.0001) **ADDITIONAL PUBLISHED BIOLOGICAL APPLICATIONS CITING THIS ASSAY:** **Hobson S, et al., 2022 [75]:** **OBJECTIVE:** ▪ To investigate the relationship between lipid profile [total, high-density lipoprotein (HDL), non-HDL, low-density lipoprotein (LDL) and triglycerides] and five uremic toxins [IxS, *p*CS, indole-3-acetic acid, trimethylamine N-oxide (TMAO), and phenylacetylglutamine] in 611 patients diagnosed with late-stage chronic kidney disease and/or kidney failure. **STUDY DESIGN:** Open-label, observational/cross-sectional **SAMPLE POPULATION (N = 611):** - Baseline parameters for the entire cohort (N = 611): Age (years) = 55 (43–67) [median (interquartile range)], body mass index (BMI) (kg/m^2^) = 24.8 (22.6–27.5), diabetes mellitus (N [%]) = 53 [8.7%], statin usage (N [%]) = 231 [37.8%], creatinine (μmol/L) = 637 (427–848), eGFR (mL/min/1.73 m^2^) = 7 (5–11). - Lipid profile: Total cholesterol (mmol/L) = 4.6 (3.9–5.5), HDL cholesterol (mmol/L) = 1.3 (1.0–1.7), non-HDL cholesterol (mmol/L) = 3.2 (2.5–4.1), LDL cholesterol (mmol/L) = 2.5 (1.8–3.3), remnant cholesterol (mmol/L) = 0.7 (0.5–0.9), triglycerides (mmol/L) = 1.4 (1.0–2.0). - Uremic toxin concentrations: - IxS: 87.8 (43.1–135.4) µM; males 92.8 (54.1–143.9) µM vs. females 80.5 (31.4–126.6) µM [*p* = 0.005]; dialysis 106.4 (73.0–154.3) vs. non-dialysis 27.9 (12.7–74.9) [*p* < 0.001]. Equivalent in µg/mL to 18.7 (9.2–28.9); males 19.8 (11.5–30.7) vs. females 17.2 (6.7–27); dialysis 22.7 (15.6–32.9) vs. non-dialysis 5.9 (2.7–15.9). - *p*CS: 133.4 (68.0–202.7) µM; males 137 (67.2–202.3) µM vs. females 127.7 (69.2–203.0) µM [*p* = 0.615]; dialysis 146.2 (85.8–207.6) vs. non-dialysis 101.1 (45.4–187.1) [*p* < 0.001]. Equivalent in µg/mL to 25.1 (12.8–38.1); males 25.8 (12.6–38.1); females 24.0 (13.0–38.2); dialysis 27.5 (16.4–39.0) vs. non-dialysis 19.0 (8.5–35.2). **AUTHORS’ KEY FINDINGS:** - Univariate correlations: - Negative correlations between IxS and total cholesterol (−0.276. *p* < 0.001666), HDL (−0.232, *p* < 0.001666), non-HDL (−0.204, *p* < 0.001666), LDL (−0.227, *p* < 0.001666) based on Spearman correlation; no significance observed with remnant cholesterol and triglycerides. - Negative correlations between *p*CS and total cholesterol (−0.203. *p* < 0.001666), non-HDL (−0.182, *p* < 0.001666), and LDL (−0.164, *p* < 0.001666); no significance observed with HDL, remnant cholesterol, and triglycerides. - Multivariate regression analysis: No significant correlations for IxS and *p*CS **te Linde E, et al., 2020 [76]:** **OBJECTIVE:** - To investigate whether kidney transplantation-associated changes in uremic toxin concentrations are correlated with improvements in cognitive functions within 3 months post-transplant. **STUDY DESIGN:** prospective, observational, matched control with CKD and HD patients **SAMPLE POPULATION (N = 28):** - Group 1: Cognitive functions assessed before transplantation (~4 weeks), after transplantation (4–6 days), and after transplantation (12 weeks): Groups 2–3: Neuropsychological assessments (NPAs) assessed at 3 distinct visits over 3–5 months (but not immediately after dialysis). - Group 1: patients with a living donor kidney transplant (N = 10; 7 males); age 53.1 (22–65, range) years old, 6.8 ± 3.1 (mean ± SD) months on dialysis. - Groups 2 and 3: matched controls on age, sex, and education (N = 18). Group 2: CKD patients on hemodialysis (HD) (N = 9; 6 males); age 60.8 (43–72) years old; 33.3 ± 35.8 months on dialysis. Group 3: CKD patients at stages 4–5, not on dialysis (N = 9; 4 males); age 59.7 (42–69) years old. **AUTHORS’ KEY FINDINGS:** Cognitive Assessments: - NPAs included “Memory, attention and concentration, information processing speed, abstract reasoning, and executive function”. - No differences between groups 1–3 in all NPAs at any visit. - Improvements in processing speed in transplant patients (−0.15, 0.19, and 0.33 for time points 1, 2, and 3, respectively) and CKD stages 4–5 patients (0.01, 0.38, and 0.46 for time points 1, 2, and 3, respectively). UT concentrations: - Serum concentrations of 16 uremic toxins were obtained within 24 h of NPAs. In kidney transplant subjects, samples were also obtained from 1–2 days before and 2–3 days after transplantation. - Uremic toxins concentrations were lower in group 3 compared to groups 1 and 2. - Group 1: IxS: 97.94, 115.66, 4.03, 5.05, and 5.57 µM equivalent to 20.88, 24.66, 0.86, 1.08, and 1.19 µg/mL (visit 1, pre-transplantation, post-transplantation, visit 2, and visit 3). *p*CS: 153.65, 203.59, 28.98, 11.66, and 13.38 µM equivalent to 28.92, 38.32, 5.45, 2.19, and 2.52 µg/mL (visit 1, pre-transplantation, post-transplantation, visit 2, and visit 3). - Groups 2 and 3 did not show significant changes in uremic toxin concentrations over time. **Biruete A, et al., 2020 [77]:** **OBJECTIVE:** - To investigate the effect of inulin administration on the gut microbiota composition and microbiota-derived metabolites in hemodialysis patients. **STUDY DESIGN:** prospective, randomized, double-blind, cross-over **SAMPLE POPULATION (N = 12):** - Hemodialysis patients (HD): age: 55 ± 10 years old, 6 males, 58% African American, BMI 31.6 ± 8.9 kg/m^2^, 33% diabetes mellitus. - Group 1: administration of inulin; 10 g/d for females; 15 g/d for males (4 weeks). - Group 2: administration of maltodextrin; 6 g/d for females; 9 g/d for males (4 weeks). **AUTHORS’ KEY FINDINGS:** - Inulin had an impact on the gut microbiota composition. - Inulin was not able to reduce the fecal concentrations of *p*-cresol or indoles or plasma concentrations of *p*CS or IxS (compared to maltodextrin, which also has an effect on gut microbiota). **Vandenbosch I, et al., 2021 [78]:** **OBJECTIVE:** - To investigate the effects of clotting with asymmetric cellulose triacetate (ATA) dialyzers in the absence of anticoagulation. **STUDY DESIGN:** prospective, open-label, randomized, cross-over **SAMPLE POPULATION (N = 20):** - Hemodialysis patients: age ≥ 18 years old (mean ± SD of 75.1 ± 12.3), on 3 sessions of dialysis/week, 4 h duration), 9 males, dialysis vintage (months): median 32 (min-max: 5–172), hemoglobin (g/dL): 10.0 ± 1.1, thrombocytes (×10^9^/L): 210 ± 66, Calcium (mmol/L): 1.11 ± 0.07, Magnesium (mmol/L): 0.92 ± 0.11, anticoagulation, %: acetylsalicylic acid (80%0), clopidogrel (5%), vitamin K antagonists (0), non-vitamin K oral anticoagulants (0). - Group 1 (Arm A): ATA dialyzer with citrate-containing dialysate. - Group 2 (Arm B): ATA dialyzer with high-volume predilution hemodiafiltration but no anticoagulation. - All patients took part in both treatment arms, 6 sessions/arm, 12 sessions/patient. **AUTHORS’ KEY FINDINGS:** - No significant differences were observed in IxS and *p*CS reduction ratios between groups 1 and 2. **Nazzal L, et al., 2021 [79]:** **OBJECTIVE:** - To investigate the effects of vancomycin on the gut microbiome and uremic toxin concentrations in patients with end-stage renal disease. **STUDY DESIGN:** Prospective, single-blinded, randomized, cross-over **SAMPLE POPULATION (N = 10):** - Hemodialysis (HD) patients: 9 males, age 56.6 ± 10.1, 3 sessions/week, ethnicity (White/Black/Asian/Hispanic/ > 1 or other): 1/2/2/4/1, duration on HD (years): 7.4 ± 7.7, length of HD session (hours): 3.6 ± 0.4, heparin (yes/no/unknown): 6/3/1, hypertension (HTN) (yes/no): 9/1, type II diabetes mellitus (DM) (yes/no): 4/6, coronary artery disease (yes/no): 2/8, diet (nonrestricted/low sodium and/or low potassium): 6/4. - Group 1 (N = 10): vancomycin oral 250 mg, once weekly post-dialysis, for 12 weeks. - Group 2 (N = 5, carryover problem resulted in half of the placebo being excluded): placebo, once weekly, for 12 weeks. **AUTHORS’ KEY FINDINGS:** - Changes in uremic toxin concentrations (changes between weeks 0–4 (μM/mL)): - IxS: placebo = 9.71 μM/mL, *p* = 0.558 and vancomycin = –25.31 μM/mL, *p* = 0.033. - *p*CS: placebo = 0.64 μM/mL, *p* = 0.980 and vancomycin = –73.51 μM/mL, *p* = 0.010. **Dai L, et al., 2020 [80]:** **OBJECTIVE:** - To investigate the association between sevelamer and uremic toxins. **STUDY DESIGN**: prospective, observational. **SAMPLE POPULATION (N = 423):** - Total cohort: CKD patients at ESRD, 277 males, age (median) 54 years old, BMI (kg/m^2^) = 24.2 (22.0–26.6), diastolic BP (mmHg): 80 (73–89), dialysis vintage (months): 24.6 (6.1–42.6), non-dialysis, N (%): 37 (9%), HD, N (%): 261 (62%), peritoneal dialysis, N (%):125 (29%) - Group 1: Sevelamer non-users, N = 251, 170 males, BMI (kg/m^2^) = 23.6 (21.5–26.0), diastolic BP (mmHg): 80 (74–89), dialysis vintage (months): 24.2 (9.4–41.1), non-dialysis, N (%): 22 (9%), HD, N (%): 152 (60%), peritoneal dialysis, N (%): 77 (31%) - Group 2: Sevelamer users, N = 172, 107 males, BMI (kg/m^2^) = 24.9 (22.5–27.8), diastolic blood pressure (DBP) (mmHg): 79 (72–89), dialysis vintage (months): 25.6 (1.7–49.8), non-dialysis, N (%):15 (9%), HD, N (%):109 (63%), peritoneal dialysis, N (%):48 (28%) - Biochemical measurements for all the patients, group 1, and group 2 (mean ± standard deviation or median with range): - Hemoglobin, g/dL: 11.8 (1.6), 11.6 (1.6), and 12.0 (1.6). *p* = 0.06 between groups 1&2 - Creatinine, mg/dL: 7.6 (5.9–9.3), 7.3 (5.4–8.8), and 7.9 (6.4–10.2). *p* < 0.001 - Calcium, mg/dL: 9.1 (8.5–9.6), 9.1 (8.5–9.6), and 9.1 (8.6–9.7). *p* = 0.34 - Phosphate, mg/dL: 4.6 (3.8–5.6), 4.3 (3.6–5.3), and 5.0 (4.1–5.9). *p* < 0.001 - Serum albumin, g/L: 40.7 (36.0–45.1), 41.7 (37.0–45.4), and 40.0 (35.0–44.8). *p* = 0.05 - Parathyroid hormone, ng/L: 168 (87–289), 166 (81–269), and 173 (96–320). *p* = 0.11 - Vitamin K status, dp-ucMGP (pmol/L): 1050 (712–1565), 952 (655–1353), and 1180 (837–1832). *p* < 0.001 - Uremic toxins for all the patients, Group 1 and Group 2: - IxS, µM: 101 (62–151), 89 (55–136), and 123 (76–161). *p* < 0.001. - *p*CS, µM: 166 (112–230), 164 (108–230), 170 (119–226). *p* = 0.60. **AUTHORS’ KEY FINDINGS:** - Sevelamer associated with higher IxS concentrations in multivariate regression analysis. **Etinger A, et al., 2018 [81]:** **OBJECTIVE:** - To investigate the effects of blood/dialysis buffer pH on the clearance of uremic toxins. **STUDY DESIGN**: Prospective, observational, self-controlled (“each patient underwent a mid-week “isohydric dialysis” followed by a “standard dialysis” two days later). **SAMPLE POPULATION (N = 8):** - Hemodialysis patients: 7 males, ages 40–71 years old; ethnicity: African-American (5), Asian (1), Caucasian (1), and Hispanic (1); months on hemodialysis: 25–77 months; etiology of renal disease: lupus nephritis (1), IgA nephropathy (1), HIV-nephropathy (1), hypertensive nephropathy (4), and diabetic nephropathy (1); patients on heparin with hemodialysis (6). - Condition 1: reduced dialysate bicarbonate buffer (i.e., 25 mmol/L) for 90 min (isohydric dialysis), then standard buffer (37 mmol/L). - Condition 2: the use of a standard buffer. **AUTHORS’ KEY FINDINGS:** - The clearance of IxS and *p*CS was not significantly affected by the dialysis conditions. **Deltombe O, et al., 2017 [82]:** **OBJECTIVE:** - To investigate the binding characteristics of uremic toxins, including IxS and *p*CS, in the following conditions: healthy control serum, blank serum from hemodialysis patients (post in vitro dialysis, charcoal-treated), and non-treated serum from hemodialysis patients. **STUDY DESIGN**: Observational blood sample collection from HD patients with healthy volunteer control **SAMPLE POPULATION (N =79):** - Healthy volunteers, N = 12: - Hemodialysis patients (HD), N = 67: blood was collected pre-dialysis, - The serum samples were pooled into 4 groups: - Group 1: healthy serum. - Group 2: blank HD serum (cleared of uremic toxins). - Group 3: non-treated HD serum - Group 4: blank serum for uremic toxin competition experiment (effects of varying concentrations of one toxin on the binding of other toxins). - Biochemical properties in groups 1 and 3, respectively: - Total Protein (g/L): 74.2 and 65.3 - Albumin (g/L): 48.1 and 40.1 **AUTHORS’ KEY FINDINGS:** - IxS and *p*CS binding capacity is higher in healthy serum than in blank HD serum (treated or untreated). - Uremic toxins concentrations in groups 1 and 3, respectively: - IxS (µM): 4.54 and 111.35, equivalent to 0.97 and 23.74 µg/mL. - *p*CS (µM): 11.63 and 196.16, equivalent to 2.19 and 36.91 µg/mL. **Keddar M, et al., 2020 [83]:** **OBJECTIVE:** - To investigate the relationship between muscle radiation attenuation (inverse relationship to amount of muscle fat) and cardiovascular events in association with *p*CS concentrations in CKD patients on peritoneal dialysis. **STUDY DESIGN**: Prospective, observational **SAMPLE POPULATION (N = 101):** - CKD patients at ESRD, N = 101 (4 patients were lost to follow-up), males: 67, age: 56 ± 18, Ethnicity% (Caucasian: 93%, Asian: 3%, African: 4%). - No CVD cohort, N = 67, males: 46, age: 53 ± 19, Ethnicity% (Caucasian: 93%, Asian: 4%, African: 3%). - CVD cohort, N = 34, males: 21, age: 62 ± 13, Ethnicity% (Caucasian: 94%, Asian: 0%, African: 6%). **AUTHORS’ KEY FINDINGS:** - Muscle radiation attenuation of 38 ± 12 Hounsfield units in no CVD vs. 31 ± 11 Hounsfield units in CVD (*p* = 0.006). - Reduction in muscle attenuation radiation is associated with an increase in serum *p*CS.	de Loor et al. 2016 [42]
**OBJECTIVE:** - To determine the associations between free or total *p*CS or IxS with kidney function markers in subjects on peritoneal dialysis. **STUDY DESIGN**: Observational, with healthy volunteer controls. **SAMPLE POPULATION (N = 230):** - CKD patients: N = 205, age 43.5 (28–61) years, body weight 64.5 Kg (52–75 Kg) - Healthy volunteers: N = 25, age 35.8 (26–48) years, body weight 65.4 Kg (48–83 Kg) - Renal function parameters (serum creatinine (SCr), blood urea nitrogen (BUN), eGFR, and serum albumin) were available in only 64 patients (numerical data not provided). **AUTHORS’ KEY FINDINGS:** - Samples were obtained after 12 h fasting for both groups (8:00 a.m.). - IxS and *p*CS concentrations in CKD patients, free and total concentrations: - IxS: 3043 ± 4253 and 32,776 ± 23,075 (CKD) vs. 2217 ± 2494 (healthy, total only) - *p*CS: 2746 ± 6042 and 20,300 ± 18,210 (CKD) vs. 1313 ± 581 (healthy, total only) [Note: unit was not provided; however, it was likely ng/mL based on the unit used to define the LLOQ] Spearman’s correlations in CKD subjects: IxS-free with: - IxS-total = 0.851 (*p* < 0.05), *p*CS-free = 0.218, SCr = 0.538 (*p* < 0.05), BUN = 0.059, eGFR = −0.549 (<0.05), Albumin = 0.136. IxS-total with: - *p*CS-free = 0.132, SCr = 0.563 (*p* < 0.05), BUN = 0.182, eGFR = −0.511(*p* < 0.05), Albumin = 0.295 (*p* < 0.05). *p*CS-free with: - *p*CS total = 0.584 (*p* < 0.05), IxS-free = 0.218, SCr = 0.106, BUN = 0.005, eGFR = 0.113, Albumin = 0.184. *p*CS-total with: - IxS-free= −0.013, SCr = −0.168, BUN = −0.233, eGFR = 0.230, Albumin = 0.124. **ADDITIONAL PUBLISHED BIOLOGICAL APPLICATIONS CITING THIS ASSAY:** **Chen X, et al., 2018 [84]:** **OBJECTIVE:** - To investigate the effects of Shen-Shuai-Ning (SSN) on the serum concentrations of IxS and *p*CS in patients on peritoneal dialysis. **STUDY DESIGN**: Prospective, randomized, controlled **SAMPLE POPULATION (N = 60):** - Group 1: N = 30, 22 males, peritoneal dialysis (PD) patients administered 5 g of SSN granules 3 × day post meals for 12 weeks. - Group 2 (control): N = 30, 18 males, peritoneal dialysis, lack of SSN. - Baseline characteristics for patients in group 1 and group 2, respectively: - Age (years): 46.23 ± 11.21 (mean ± SD) and 45.57 ± 12.41. *p* = 0.83. - PD duration (months): 18.77 ± 13.19 and 17.57 ± 17.92. *p* = 0.77. - Mean arterial pressure (MAP) (mmHg): 99.18 ± 14.17 and 103.72 ± 12.43. *p* = 0.20. - BMI (kg/m^2^): 22.70 ± 3.11 and 22.37 ± 3.24. *p* = 0.69. - Cause of ESRD. Chronic glomerulonephritis (CGN): 18 (60.0%) and 16 (53.4%). *p* = 0.85. Diabetes: 2 (10.0%) and 4 (13.3%). Hypertensive nephropathy: 4 (10.0%) and 4 (13.3%). - Baseline uremic toxins concentrations for Groups 1 and 2: Total IxS: 32.33 ± 20.18 and 29.40 ± 16.85 µg/mL (*p* = 0.54) Free IxS: 2.94 ± 2.88 and 1.99 ± 1.9 µg/mL (*p* = 0.14) Total *p*CS: 23.90 ± 19.87 and 17.90 ± 15.03 µg/mL (*p* = 0.19) Free *p*CS: 2.03 ± 1.92 and 1.41 ± 1.14 µg/mL (*p* = 0.14) **AUTHORS’ KEY FINDINGS:** - Concentration results at 4 weeks, µg/mL, for Groups 1 and 2: Total IxS: 27.28 ± 18.19 and 39.25 ± 20.23 (*p* = 0.02) Free IxS: 2.23 ± 2.42 and 2.76 ± 2.70 (*p* = 0.44) Total *p*CS: 18.85 ± 13.79 and 26.87 ± 22.99 (*p* = 0.12) Free *p*CS: 1.72 ± 1.68 and 2.08 ± 2.52 (*p* = 0.53) - Concentration results at 8 weeks, µg/mL, for Groups 1 and 2: Total IxS: 29.73 ± 19.10 and 43.86 ± 23.91 (*p* = 0.02) Free IxS: 2.54 ± 3.39 and 3.27 ± 2.33 (*p* = 0.36) Total *p*CS: 13.47 ± 8.50 and 17.60 ± 12.36 (*p* = 0.16) Free *p*CS: 1.03 ± 1.16 and 1.49 ± 1.46 (*p* = 0.21) - Concentration results at 12 weeks, µg/mL, for Groups 1 and 2: Total IxS: 29.41 ± 17.61 and 45.34 ± 33.52 (*p* = 0.04) Free IxS: 2.40 ± 2.26 and 3.49 ± 2.53 (*p* = 0.11) Total *p*CS: 19.12 ± 16.31 and 20.73 ± 12.94 (*p* = 0.70) Free *p*CS: 1.45 ± 1.35 and 1.49 ± 1.37 (*p* = 0.70) **Wang SC, et al., 2021 [85]:** **OBJECTIVE:** - To investigate the relationship between serum IxS concentrations and arterial stiffness (AS; defined as carotid-femoral pulse wave velocity (cmpVW) > 10 m/s) in CKD patients. **STUDY DESIGN**: Prospective, observational (cross-sectional) **SAMPLE POPULATION (N = 155):** - CKD patients: N = 155, age 66.65 ± 13.51 (mean ± SD) years old, 82 men, eGFR 40.40 ± 21.86 mL/min (patients with missing data, acute infection, acute myocardial infarction or heart failure and cancer were not included) - CKD stage 1: 8 patients, stage 2: 14 patients, stage 3: 75 patients, stage 4: 41 patients, and stage 5: 17 patients - **Group 1**: do not have AS, N = 104/155, - Age 63.75 ± 13.39 years, 53 men, BMI 26.30 ± 4.78 (sample size for underweight, normal, overweight, and obesity were 4, 37, 43, and 20) - cmpVW 7.68 ± 1.46 m/s - Patients with diabetes are 38, and patients with hypertension are 85 - Systolic blood pressure (SBP), mmHg: 143.18 ± 20.71 - Creatinine, mg/dL: 1.60 (1.20–2.28) - eGFR 43.41 ± 23.54 mL/min, CKD stage 1: 7 patients, CKD stage 2: 12 patients, CKD stage 3: 50 patients, CKD stage 4: 25 patients, and CKD stage 5: 10 patients - Total cholesterol, mg/dL: 159.48 ± 44.53 - **Group 2**: have AS, N = 51/155 - age 72.55 ± 11.81 years, 29 men, BMI 26.25 ± 3.53 (sample size for underweight, normal, overweight, and obesity were 0, 20, 23, and 8) - cmpVW 12.75 ± 1.96 m/s - patients with diabetes are 28, patients with hypertension are 37 - Systolic blood pressure (SBP), mmHg: 154.02 ± 22.81 - Creatinine, mg/dL: 1.70 (1.40–2.20) - eGFR 34.26 ± 16.52 mL/min, stage 1: 1 patient, stage 2: 2 patients, stage 3: 25 patients, stage 4: 16 patients, and stage 5: 7 patients - Hypertension (N = 122): blood pressure > 140/90 mmHg - Diabetes (N = 66): fasting plasma glucose concentration of 126 mg/dL - Carotid-femoral pulse wave velocity was measured by an applanation tonometer. **AUTHORS’ KEY FINDINGS:** - IxS concentrations, mean (minimum–Maximum), µg/mL, for all cohorts, Group 1 and Group 2, respectively: - 1.06 (0.55–2.17), 0.88 (0.43–1.76), and 1.53 (0.70–3.68). *p* < 0.001 for Groups 1 and 2 - CKD patients with AS are older compared to patients without AS (*p* < 0.001), with a higher proportion of diabetes (*p* = 0.03), higher SBP (*p* = 0.004), and lower eGFR (*p* = 0.014), respectively - A significant negative correlation is between IxS and eGFR (r = 0.57, *p* < 0.001) - IxS concentrations in different CKD stages, µg/mL (*p* < 0.001): Stage 1: 0.143 ± 0.160 (N = 8), stage 2: 0.622 ± 0.558 (N = 14), stage 3: 1.045 ±0.852 (N = 75), stage 4: 2.202 ± 1.413 (N = 41), stage 5: 5.560 ± 2.935 (N = 17) - The correlation based on multiple regression between log IxS and AS in CKD patients was R^2^ = 0.133 (*p* < 0.003)	Shu et al. 2016 [37]
**OBJECTIVE:** - To quantify tryptophan and its metabolites in human serum in patients with ESRD **STUDY DESIGN**: Prospective, matched-control (matching by age, gender, and ethnicity) **SAMPLE POPULATION (N = 10):** - Patients with end-stage renal disease (ESRD) on hemodialysis, males, age 43 (27–51) years, body mass index 26.1 (17–31) - Underlying cause for ESRD: diabetes and/or hypertension - Control: matched age, gender, and ethnic background - Serum samples were obtained before performing the dialysis procedure **AUTHORS’ KEY FINDINGS:** - IxS concentrations in ESRD (~60 µM or 12.8 µg/mL) were higher than the control (~3 µM or 0.64 µg/mL, *p* = 0.0001) (data provided in figure form in the original manuscript) - Tryptophan had lower concentrations in pre-dialysis samples from ESRD patients compared to controls (*p* = 0.007) **ADDITIONAL PUBLISHED BIOLOGICAL APPLICATIONS CITING THIS ASSAY:** **Kolachalama VB, et al., 2018 [86]:** **OBJECTIVE:** - To determine the relationships between the uremic solute–aryl hydrocarbon receptor (AHR)–tissue factor (TF) thrombosis in CKD patients after a vascular injury. **STUDY DESIGN**: cross-sectional **SAMPLE POPULATION (N = 470):** - Group 1: CKD patients with thrombosis, N = 60, 33 men, age 54.69 ± 15.85 years, BMI 29.76 ± 8.20 kg/m^2^ - SBP 138 ± 20.77 mmHg (mean ± SD); patients with diabetes, cardiovascular disease (CVD), cerebrovascular disease, and venous thromboembolism disease were 30, 11, 3, and 3, respectively - 47 patients were on aspirin, 19 patients were on statin, and 22 patients were on clopidogrel - 39 are on HD (65%) - Group 2: CKD patients without thrombosis, N = 410, 278 men, age 52.35 ± 13.88 years, BMI 30.02 ± 8.49 kg/m^2^ - SBP 140 ± 21.28 mmHg, patients with diabetes, CVD, cerebrovascular disease, and venous thromboembolism disease were 208, 90, 28, and 11, respectively - 337 patients were on aspirin, 108 patients were on statins, and 203 patients were on clopidogrel - 240 on HD (58.5%) **AUTHORS’ KEY FINDINGS:** - The correlations were IxS-AHR activity (R^2^ = 0.59, *p* < 0.001) and IxS-TF activity (R^2^ = 0.24, *p* < 0.05)	Zhang et al. 2017 [43]
**OBJECTIVE:** - To compare total and free IxS and *p*CS in serum samples between healthy volunteers and patients with CKD. - To determine the relationships between IxS or *p*CS concentrations and stages of CKD. **STUDY DESIGN**: cross-sectional with healthy volunteer control **SAMPLE POPULATION:** - Group 1: clinically stable CKD, N = 205, age > 20 years, patients were classified into CKD stages according to the renal function determined by their eGFR. CKD 1: N = 29, 4 males, age 62.2 ± 10.4 CKD 2: N = 49, 32 males, age 64.4 ± 7.4 CKD 3: N = 64, 40 males, age 66.4 ± 8.2 CKD 4: N = 40, 24 males, age 69.1 ± 10.9 CKD 5: N = 22, 12 males, age 68.35 ± 8.0 Healthy volunteer: N = 45 (no demographic provided) **AUTHORS’ KEY FINDINGS:** - Concentrations of IxS and *p*CS in healthy volunteers (min–max), patients with CKD stages at stages 1, 2, 3, 4, and 5 (mean ± SD) (µg/mL): Total IxS: <0.05–3.02, 1.03 ± 0.85, 1.54 ± 1.11, 2.22 ± 1.79, 4.74 ± 4.34, 18.21 ± 15.06 (*p* < 0.001 for the non-parametric reference range ≤0.05–1.15 µg/mL) Total *p*CS: <0.05–9.87, 2.69 ± 4.34, 4.42 ± 4.47, 6.45 ± 7.12, 16.10 ± 13.98, 27.00 ± 17.66 (*p* < 0.001) Free IxS: not detectable in 43 healthy volunteers, 0.08 ± 0.06, 0.11 ± 0.09, 0.17 ± 0.13, 0.49 ± 0.72, 2.36 ± 2.64 (*p* < 0.001) Free *p*CS: not detectable in 38 healthy volunteers, 0.15 ± 0.20, 0.24 ± 0.29, 0.36 ± 0.37, 1.36 ± 2.58, 2.38 ± 2.03 (*p* < 0.001) **ADDITIONAL PUBLISHED BIOLOGICAL APPLICATIONS CITING THIS ASSAY:** **Lai YR, et al., 2022 [87]:** **OBJECTIVE:** - To investigate the relationships between IxS or *p*CS and peripheral nerve function in pre-dialysis CKD patients. **STUDY DESIGN:** Observational. **SAMPLE POPULATION (N = 54):** - Group 1: diabetic CKD, N = 27. - Group 2: non-diabetic CKD, N = 27. Baseline parameters for group 1 and group 2, respectively: - Age (year): 69.7 ± 12.4 and 67.0 ± 10.2. *p* = 0.22. - Males: 16 and 18. *p* = 0.57. - Diabetes duration (year): 16.2 ± 11.1 and not diabetic. - BMI: 26.5 ± 5.4 and 26.5 ± 5.4. *p* = 0.11. - SBP (mmHg): 140.5 ± 23.2 and 151.9 ± 25.6. *p* = 0.09. - DBP (mmHg): 75.3 ± 14.9 and 81.9 ± 16.5. *p* = 0.13. - Patients on ACE inhibitor or angiotensin II receptor blocker: 20 and 22. *p* = 0.51. - Patients on beta-blockers: 13 and 13. *p* = 1.0. - Patients on calcium channel blocker: 18 and 14. *p* = 0.27. - Patients on diuretics: 8 and 1. *p* = 0.02. - Patients on alpha-blocker: 4 and 3. *p* = 1.0. - Patients on antiplatelet medications: 13 and 9. *p* = 0.27. - Patients on lipid-lowering medications: 22 and 15. *p* = 0.04. - Composite amplitude scores (CAS) were used to measure the severity of nerve conductions. **AUTHORS’ KEY FINDINGS:** - IxS and *p*CS concentrations in Group 1 and Group 2, respectively (μg/mL): Free-form IxS: 0.24 ± 0.17 and 0.21 ± 0.15, *p* = 0.54 Free-form *p*CS: 0.50 ± 0.49 and 0.45 ± 0.41, *p* = 0.73 Total-form IxS: 4.0 ± 3.3 and 3.9 ± 2.3, *p* = 0.88 Total-form *p*CS: 11.6 ± 10.4 and 10.4 ± 9.8, *p* = 0.65 - CAS was correlated with free-IxS in single-variable regression (R = 0.39, *p* = 0.009). Data for *p*CS not provided. **Wu IW, et al. 2020, a [88]:** Assay: cited Lin 2019, but used an apparently different ultra-centrifugal filter **OBJECTIVE:** - To investigate the effects of a low protein diet (LPD) on gut microbiota and gut-derived metabolites (including IxS and *p*CS) in CKD patients. **STUDY DESIGN:** Prospective, controlled study, non-randomized, open-label, with comparison to healthy volunteers matched by age, gender, and diabetes or hypertension. **SAMPLE POPULATION (N = 77):** - Group 1: CKD patients at LPD, N = 16, <0.8 g/kg body weight/day for 3 months. - Group 2: CKD patients on a normal protein diet (NPD). N = 27, 1 g/kg body weight/day for 3 months. - Group 3: matched normal non-CKD controls, N = 34. **AUTHORS’ KEY FINDINGS:** - Significant changes in microbiota composition were recorded between Group 1 and 2. (*p* < 0.01) - No significant differences in IxS and *p*CS concentrations between the CKD groups and when compared to the non-CKD group (graphically). - IxS and *p*CS concentrations in Groups 1 and 2, respectively (μg/mL): Total IxS: 2.7 ± 5.87 and 4.54 ±13.84, *p* = 0.257 Total *p*CS: 8.66 ± 23.76 and 5.45 ± 20.5. *p* = 0.946 Free IxS: 0.26 ± 0.94 and 0.34 ± 1.56. *p* = 0.257 Free *p*CS: 0.64 ± 1.23 and 0.39 ± 1.30. *p* = 0.796 **Wu IW, et al. 2020, b [89]:** Assay: cited Lin 2019, but used an apparently different ultra centrifugal filter and a slightly different mobile phase (addition of 1 mM ammonium acetate to organic phase) **OBJECTIVE:** - To investigate the correlations between gut microbiota, uremic toxins, and renal function in CDK patients. **STUDY DESIGN:** Prospective, controlled study, non-randomized, open-label, with comparison to healthy volunteers matched by age, gender, and diabetes or hypertension. **SAMPLE POPULATION (N = 130):** - Group 1: healthy control, N = 30. - Group 2: mild CKD, N = 31. - Group 3: moderate CKD, N = 30. - Group 4: advanced CKD, N = 31. - Group 5: advanced CKD treated with AST-120 (Kremezin 6 g/day for 12 weeks), N = 8. The baseline parameters for Groups 1, 2, 3, 4, and 5, respectively: - Age (years): 61.6 ± 8.7, 62.4 ± 4.1, 63.6 ± 6.1, 66.2 ± 7.4, and 67 ± 10.1 - Male (%): 12 (40%), 14 (45.2%), 18 (60%), 15 (48.4%), and 2 (25%) - Diabetes, N (%): 19 (63.3%), 16 (51.6%), 15 (50%), 17 (54.8%), and 3 (37.5%) - HTN, N (%): 27 (87.1%), 23 (76.7%), 26 (86.7%), 30 (96.8%), and 6 (75%) - DBP, mm Hg: 74.1 ± 9.5, 75.4 ± 12.1, 75 ± 8.4, 75.3 ± 12.3, and 71.6 ± 5.6 - SBP, mm Hg: 130.1 ± 18.4, 132.3 ± 17.2, 127.3 ± 13.9, 137.9 ± 14.3, and 139.8 ± 16.2 - BMI, Kg/m^2^: 25.5 ± 3.4, 27.5 ± 3.6, 26.5 ± 4.2, 25.9 ± 4.3, and 25.9 ± 4.3 - BUN, mg/dL: 13.4 ± 3.9, 16.5 ± 4.8, 19.6 ± 5.9, 61 ± 26.1 *, and 69.3 ± 39.4 * - SCr, mg/dL: 0.7 ± 0.2, 1.0 ± 0.2, 1.4 ± 0.5, 4.4 ± 2.3 *, and 6.1 ± 2.9 * - eGFR, mL/min/m^2^: 112.4 ± 54.4, 71.4 ± 22.1 *, 49.9 ± 10.2 *, 16.2 ± 10 *, and 14.1 ± 18.6 * - Hemoglobin, g/dL: 13.3 ± 0.9, 13.9 ± 1.3, 12.9 ± 1.3, 10.0 ± 1.9 *, and 9.6 ± 1.6 * - Serum albumin, mg/dL: 4.6 ± 0.3, 4.6 ± 0.3, 4.5 ± 0.3, 4.1 ± 0.6 *, and 4.2 ± 0.4 * - Total cholesterol, mg/dL: 191.42 ± 30.77, 186.52 ± 25.32, 171.5 ± 29.26, 193.66 ± 47.84, and 152.56 ± 46.35 - LDL-cholesterol, mg/dL: 111.46 ± 30.61, 107.55 ± 22.58, 95.35 ± 25.33, 109.05 ± 35.16, and 86.06 ± 40.58 - Triglyceride, mg/dL: 159.39 ± 99.92, 129.61 ± 65.29, 141.8 ± 83.11, 182.25 ± 153.55, and 115 ± 81.04 - Estimated protein intake, g/day: 77.9 ± 28.2, 70.6 ± 22.6, 58.6 ± 21, 58.6 ± 21.1, and 57.5 ± 20.6 * *p* < 0.005 vs. non-CKD **AUTHORS’ KEY FINDINGS:** - 7 genera and 2 species of microbiota were correlated with CKD stages. - Free and total IxS and *p*CS concentrations significantly elevated in advanced CKD (graphical data). - Several bacterial genera correlated with total and free IxS and *p*CS concentrations. **Cheng BC, et al. 2020 [90]:** Assay: cited Lin 2019 but with different sample/acetonitrile volume, centrifugation duration, and vortex duration. **OBJECTIVE:** - To determine the relationships between peritoneal dialysis (PD), cardiovascular autonomic function (CAF), and serum concentrations of IxS and *p*CS. **STUDY DESIGN**: Prospective, controlled with pre-dialysis CKD patients and with healthy volunteers matched on age and sex. **SAMPLE POPULATION (N = 75):** - Group 1: ESRD patients undergoing PD, N = 26 (PD group) - Group 2: ND CKD patients, N = 19 (disease group) - Group 3: matched healthy volunteers, N = 30 (control group) Baseline characteristics for Groups 1, 2, and 3, respectively: - Age (years) (mean ± SD): 56.4 ± 9.6, 66.8 ± 8.0, and 59.9 ± 6.5. *p* = 0.12 - Males: 15, 12, and 20. *p* = 0.14 - Underlying diseases: HTN (N, %): 19 (73.1), 13 (68.4), no data for healthy control DM (N, %): 12 (46.1), 14 (73.7), no data for healthy control Dyslipidemia (N, %): 8 (30.7), 6 (31.6), no data for healthy control CVD history (N, %): 2 (7.7), 1 (5.2), no data for healthy control Alcoholism: 4 (15.4), no data for disease, no data for healthy control Smoking: 3 (11.5), no data for disease, no data for healthy control - Biochemistry data: Total cholesterol (mmol/L): 4.86 ± 1.19, 4.44 ± 1.36, and 4.92 ± 0.72. *p* = 0.43 LDL-cholesterol (mmol/L): 2.61 ± 0.91, 4.05 ± 0.79, and 2.90 ± 0.67. *p* = 0.59 Hemoglobin, g/L: 99 ± 15, 123 ± 20, and 133 ± 15. *p* = 0.41 hematocrit (%): 29.8 ± 4.9, 36.9 ± 5.4, and 40.4 ± 3.6. *p* = 0.20 eGFR (mL/min/1.73 m^2^): 5.2 ± 2.1, 39.8 ± 13.6, and 102.7 ± 15.4. *p* < 0.001 BUN (mmol/L): 35.1 ± 17.9, 12.0 ± 11.6, and 5.2 ± 1.2. *p* < 0.001 Creatinine (mmol/L): 1043.1 ± 300.6, 167.9 ± 88.4, and 61.9 ± 8.8. *p* < 0.001 Calcium (µmol/L): 2.18 ± 0.3, 2.25 ± 0.05, and 2.28 ± 0.08. *p* = 0.001 Phosphorus (mmol/L): 1.52 ± 0.48, 1.19 ± 0.32, NA - Protein-bound uremic toxin: IxS (μg/mL): 16.0 ± 8.7, NA, and 2.1 ± 1.4. *p* < 0.001 *p*CS (μg/mL): 4.4 ± 2.6, NA, and 2.7 ± 2.7. 0.026 The CAF parameters were assessed one day before PD and 6 months after maintained PD. The CAF parameters included heart rate, arterial blood pressure, heart rate response to deep breathing (HR_DB), Valsalva ratio, and baroreflex sensitivity. CAF in Group 1 (one day before PD), Group 2 (disease group), and Group 3 (control group), respectively: - Spectral analysis: Low frequency (LF), normalized unit: 40.3 ± 21.3, 41.9 ± 22.4, and 54.4 ± 17.7. *p* = 0.042 (Group 1 vs. Group 3) High frequency (HF), normalized unit: 57.1 ± 20.1, 57.8 ± 22.5, 45.4 ± 17.7. *p* = 0.047 (Group 1 vs. Group 3) LF/HF ratio: 1.0 ± 0.93, 1.1 ± 1.1, and 1.6 ± 1.1. *p* = 0.045 (Group 1 vs. Group 3) - Cardio-vagal autonomic function: Valsalva ratio: 1.3 ± 0.3, 1.3 ± 0.1, and 1.5 ± 0.2. *p* = 0.0008 (Group 1 vs. Group 3) and *p* = 0.011 (Group 2 vs. Group 3) HR_DB (beats/minute): 5.3 ± 3.9, 5.4 ± 3.7, and 10.6 ± 7.3. *p* < 0.0001 (Group 1 vs. Group 3) and *p* = 0.001 (Group 2 vs. Group 3) Baroreflex sensitivity: 4.8 ± 2.5, 4.3 ± 2.8, and 7.6 ± 3.2. *p* < 0.0001 (Group 1 vs. Group 3) and *p* < 0.0001 (Group 2 vs. Group 3) **AUTHORS’ KEY FINDINGS:** - CAF parameters for group 1 before PD and 6 months after PD: Frequency domain LF, normalized unit: 40.3 ± 21.3 and 55.3 ± 20.7. *p* = 0.03 HF, normalized unit: 57.1 ± 20.1 and 43.1 ± 20.3. *p* = 0.045 LF/HF: 1.1 ± 0.9 and 2.0 ± 1.4. *p* = 0.03 Valsalva ratio: 1.2 ± 0.3 and 1.3 ± 0.2. *p* = 0.97 HR_DB (beats/min): 5.1 ± 3.9 and 5.8 ± 3.6. *p* = 0.26 Baroreflex sensitivity: 4.5 ± 2.5 and 5.6 ± 3.7. *p* = 0.26 - IxS and *p*CS for group 1 before PD and 6 months after PD, µg/mL: IxS: 17.6 ± 8.7 and 11.7 ± 5.3. *p* = 0.004 *p*CS: 4.6 ± 2.7 and 3.7 ± 1.9. *p* = 0.17 - Biochemistry characteristics for group 1 before PD and 6 months after PD: SBP (mmHg): 157.4 ± 40.9 and 134.2 ± 14.9. *p* = 0.192 DBP (mmHg): 87.0 ± 17.9 and 77.6 ± 8.4. *p* = 0.191 Hemoglobin (g/L): 98 ± 11 and 105 ± 13. *p* = 0.054 Albumin (g/L): 35 ± 5 and 35 ± 4. *p* = 0.587 - Correlations between IxS and *p*CS and CAF, Pearson correlations: - IxS with: Change in *p*CS: R = 0.653, *p* = 0.001 Change in sequence of baroreflex sensitivity: R = 0.357, *p* = 0.175 Change in LF/HF ratio: R = −0.510, *p* = 0.036 Change in HR_DB: R = −0.294, *p* = 0.237 Change in Valsalva ratio: R = −0.021, *p* = 0.965 - *p*CS with: Change in IS: R = 0.653, *p* = 0.001 Change in sequence of baroreflex sensitivity: R = −0.340, *p* = 0.198 Change in LF/HF ratio: R = −0.435, *p* = 0.081 Change in HR_DB: R = −0.263, *p* = 0.291 Change in Valsalva ratio: R = 0.078, *p* = 0.867 **Hsu CK, et al., 2022 [91]:** Assay: cited Lin 2019 but used a different ultra centrifugal filter. **OBJECTIVE:** - To investigate the effects of AST-120 on gut microbiota and uremic toxins in advanced CKD patients. **STUDY DESIGN:** Prospective, controlled with CKD patients not administered AST-120 and healthy volunteers matched by age, sex, diabetes, and hypertension. **SAMPLE POPULATION (N = 56):** - Group 1: healthy control, N = 24, Age 64.04 ± 6.54 years old, 12 males, 9 with diabetes, 15 with hypertension, none with gout, 11 with hyperlipidemia, SBP (mmHg) = 128.42 ± 17.02, BMI (kg/m^2^) = 25.87 ± 4.09, BUN (mg/dL) = 14.42 ± 3.62, creatinine (mg/dL) = 0.78 ± 0.19, eGFR (mL/min/m^2^) = 102.7 ± 63.94, fasting sugar (mg/dL) = 119.42 ± 31.28, total cholesterol(mg/dL) = 192.71 ± 25.05, Urine protein-creatinine ratio (mg/g) =81.5 (122.21; median and range). - Group 2: CKD, N = 24, 7 at CKD stage 4, 17 at CKD stage 5, Age 68.04 ± 7.31 years, 10 males, 12 with diabetes, 23 with hypertension, 4 with gout, 12 with hyperlipidemia, SBP (mmHg) = 138.71 ± 14.59, BMI (kg/m^2^) = 25.4 ± 4.1, BUN (mg/dL) = 62.96 ± 22.99, creatinine (mg/dL) = 4.51 ± 1.95, eGFR (mL/min/m^2^) = 13.94 ± 7.09, fasting sugar (mg/dL) = 116.58 ± 45.98, total cholesterol (mg/dL) = 191.04 ± 46.66, Urine protein-creatinine ratio (mg/g) = 2144 (1582.46). - Group 3: CKD + AST (6 g/day for 3 months), N = 8, 1 at CKD stage 4, 7 at CKD stage 5, - Age 67.5 ± 10.67 years, 2 males, 2 with diabetes, 6 with hypertension, 2 with gout, 2 with hyperlipidemia, SBP (mmHg) = 138.57 ± 17.13, BMI (kg/m^2^) = 26.23 ± 4.08, BUN (mg/dL) = 59.96 ± 29.57, creatinine (mg/dL) = 5.68 ± 2.83, eGFR (mL/min/m^2^) = 15.24 ± 19.48, fasting sugar (mg/dL) = 105.43 ± 16.72, total cholesterol (mg/dL) = 152.5 ± 49.55, Urine protein-creatinine ratio (mg/g) = 1279.5 (701). Clinical samples were collected after 3 months of administering AST-120. - *p* > 0.05 for all group comparisons **AUTHORS’ KEY FINDINGS:** - The gut microbial architecture: the predominance genus is Escherichia-Shigella, 11.9% in group 3, 8.9% in group 2, and 3.5% in group 1 (*p* = 0.002). - Reduction in microbial abundance in Group 3 compared to Group 2 and Group 1 for Fusicatenibacter (1.5% vs. 2.5% vs. 2.9%, *p* = 0.029), Subdoligranulum (1.3% vs. 2.6% vs. 3.3%, *p* = 0.005) and Faecalibacterium (3.3% vs. 4.4% vs. 7.5%, *p* = 0.003). - Uremic toxins: Total and free *p*CS: based on graphical assessment, a trend in reduction was observed in Group 3 vs. Group 2 (*p* > 0.05). Total and free IxS: No significant changes were reported between Groups 2 and 3.	Lin et al. 2019 [29]
**OBJECTIVE:** - To quantify uremic toxins (including IxS and *p*CS) from human serum samples from one healthy volunteer and one CKD patient. **SAMPLE POPULATION (N = 2):** **AUTHORS’ KEY FINDINGS:** - Data were available for one CKD patient and one healthy volunteer. IxS: in one CKD patient = 4.73 µg/mL and in one healthy volunteers 0.25 µg/mL *p*CS: in one CKD patient = 22.2 µg/mL and in one healthy volunteers 0.29 µg/mL **[Note: no additional human biological applications were identified]**	Prokopienko et al. 2019 [44]
**OBJECTIVE:** - To quantify uremic toxins serum concentrations in patients with acute kidney injury (AKI) post-cardiac surgery. **STUDY DESIGN:** Observational. **SAMPLE POPULATION (N = 8):** - Adults hospitalized in the intensive care unit (ICU) after cardiac surgery, N = 8, 7 males, age (year): 67.75 (42–83; mean with range), BMI (kg m^−2^): 28.60 (22.9–39.1). - Patients with AKI started within 72 h of ICU admission. - Creatinine: Basal concentration = 98.875 (70–171) μM Peak concentration = 254.25 (107–493) μM Time to creatinine peak concentration (h): 64.25 (34–137) Time to return to baseline concentration (h): 114.25 (23–181) - IxS: Basal concentration = 0.56 (0.23–0.97) μg/mL Peak concentration = 1.52 (0.35–2.62) μg/mL Time to IxS peak concentration (h): 63.50 (23–174) Time to return to baseline concentration (h): 39.83 (7–87) - *p*CS: Basal concentration = 3.30 (1.24–8.75) μg/mL Peak concentration = 9.33 (4.30–16.00) μg/mL Time to *p*CS peak concentration (h): 73.26 (23–213) Time to return to baseline concentration (h): 95.20 (27–148) **AUTHORS’ KEY FINDINGS:** - Serum peak IxS and *p*CS concentrations occurred later than peak creatinine concentrations. - According to the authors, when compared to external references, IxS and *p*CS concentrations in AKI patients were higher than in patients with no renal impairment but less than the concentrations reported in patients with chronic kidney disease (CKD) at stages 4 and 5. **ADDITIONAL PUBLISHED BIOLOGICAL APPLICATIONS CITING THIS ASSAY:** **André C, et al., 2021 [92]:** **OBJECTIVE:** - To investigate the effects of concurrent OAT1/OAT3 inhibitors on uremic toxin serum concentrations in adult kidney transplant patients. **STUDY DESIGN:** Observational. **SAMPLE POPULATION (N = 403):** - All cohort (N = 403): kidney transplant patients on calcineurin inhibitors as part of the combination immunosuppression. - Group 1: Patients not receiving concurrent OAT1/OAT3 inhibitors. (N = 92), age 52 (43–61, median with range) years, 59 males, BMI 24.6 (22.5–28.4) kg/m^2^, time since transplantation 76.0 (39.8–218.3) months, CKD 1 (1), CKD 2 (17), CKD 3 (57), CKD 4 (16), and CKD 5 (1); underlying cause of CKD: Vascular disease (1), CGN (12), polycystic kidney disease (15), interstitial nephritis (1), autoimmune disorder (17), genetic disorder (15), other causes (31); SBP 142 (130–156) mmHg, DBP 81 (74–90) mmHg, hypertension (90). Patients on cyclosporin: 38, patients on tacrolimus: 54. - Group 2: Patients receiving at least one concurrent OAT1/OAT3 inhibitor. (N = 311). age 58 (49–670) years (*p* = 0.002), 191 males, BMI 26.9 (23.9–30.4) (*p* < 0.001), time since the kidney transplantation 78.0 (40.0–146.5) months, CKD 1 (12), CKD 2 (51), CKD 3 (171), CKD 4 (68), and CKD 5 (9); the underlying cause of CKD: diabetes (20), vascular disease (25), CGN (26), polycystic kidney disease (47) (*p* = 0.043), interstitial nephritis (4), autoimmune disorder (55), genetic disorder (30), other causes (104); SBP 142 (132–158) mmHg, DBP 80 (72–86) mmHg, hypertension (301). Patients on cyclosporin: 117, patients on tacrolimus: 194. **AUTHORS’ KEY FINDINGS:** Group 1 and Group 2 uremic toxins concentrations and biochemistry data: - eGFR (mL/min/1.73 m^2^): 42.5 (34–57) and 39 (30–57), (*p* = 0.128) - Creatinine (μmol/L): 142 (116–177) and 148 (116–192), (*p* = 0.387) - Calcium (mM): 2.41 (2.33–2.50) and 2.41 (2.32–2.49), (*p* = 0.769) - Phosphate (mM): 1.03 (0.89–1.13) and 1.05 (0.91–1.23), (*p* = 0.084) - Uric acid (μM): 483 (399–540) and 458 (388–573), (*p* = 0.906) - C-reactive protein (mg/L): 1.80 (0.30–4.20) and 4.50 (0.90–11.20), (*p* = 0.007) - Protein (g/L): 67 (63–69) and 67 (64–70), (*p* = 0.984) - Albumin (g/L): 39.8 (38.0–41.7) and 38.8 (36.5–40.9), (*p* = 0.010) - Glucose (mM): 5.1 (4.6–5.8) and 5.5 (4.9–6.5), (*p* < 0.001) - IxS (μg/mL): 1.86 (1.09–3.04) and 2.30 (1.29–3.60), (*p* = 0.030) - *p*CS (μg/mL): 2.90 (1.52–5.48) and 4.96 (1.57–8.56), (*p* = 0.008) Univariate logistic regression of IxS and concurrent OAT1/OAT3 inhibitor (*p* = 0.062). Univariate logistic regression of *p*CS and concurrent OAT1/OAT3 inhibitor (*p* = 0.001); Multi-variate logistic regression (incorporating age, eGFR, albumin) of *p*CS and concurrent OAT1/OAT3 (*p* = 0.007). **André C, et al., 2022 [93]:** **OBJECTIVE:** - To determine relationships between immunosuppressants (tacrolimus and cyclosporin) and uremic toxin serum concentrations in adult kidney transplant patients. **STUDY DESIGN:** Cross-sectional. **SAMPLE POPULATION (N = 403):** - Kidney transplant recipients: - Group 1 (N = 203): eGFR > 40 mL/min/1.73 m^2^ - Group 2 (N = 200): eGFR ≤ 40 mL/min/1.73 m^2^ - Population parameters for all cohort, group 1, and group 2, respectively: Males, n (%): 250 (62.0), 142 (69.9), and 108 (54.0). *p* = 0.001 Age (years), median (IQR): 56.0 (48.0–66.0), 54.00 (44.5–64.0), and 59.0 (50.8–90.1). *p* = 0.0001 BMI (kg/m^2^), median (IQR): 26.5 (23.4–29.7), 26.3 (23.0–29.4), and 26.5 (23.7–30.4). *p* = 0.153 - Clinical characteristics: CKD stage, N (%): CKD1: 13 (3.2), 13 (6.4), NA, *p* = NA CKD2: 268 (16.9), 68 (33.5), NA, *p* = NA CKD3: 228 (56.5), 122 (60.1), 106 (53.0), *p* = NA CKD4: 84 (20.8), NA, 84 (42.0). *p* = NA CKD5: 10 (2.5), NA, 10 (5.0). *p* = NA - Cause of CKD, N (%), *p* = 0.840: Diabetes: 20 (4.9), 12 (5.9), and 8 (4.0) Vascular disorder: 26 (6.5), 10 (4.9), and 16 (8.0) CGN: 38 (9.4), 16 (7.9), and 22 (11.0) Polycystic kidney disease: 63 (15.4), 31 (15.3), and 31 (15.5) Interstitial nephritis: 5 (1.2), 2 (1.0), and 3 (1.5) Autoimmune disease: 72 (17.9), 36 (17.7), and 36 (18.0) Genetic disease: 45 (11.2), 24 (11.8), and 21 (10.5) Other causes: 106 (26.3), 57 (28.1), and 49 (24.5) Several causes: 29 (7.2), 15 (7.4), and 14 (7.0) - Time since transplantation (months), median (IQR): 78.0 (40.0–158.5), 65.0 (32.5–138.0), and 85.0 (48.0–179.2). *p* = 0.003 - SBP (mmHg), median (IQR): 142 (132–158), 140 (131–154), and 144 (132–160). *p* = 0.160 - DBP (mmHg), median (IQR): 80 (72–87), 80 (74–85), and 80 (70.0–90). *p* = 0.720 - HTN, n (%): 392 (97), 195 (96), and 196 (98). *p* = 0.381 - Liver disease, n (%): 1 (0.3), 0 (0.0), and 1 (0.5). *p* = 0.496 - Immunosuppressant treatments: - Cyclosporine, N = 155: Plasma concentration (ng/mL), median (IQR): 94.30 (74.20–123.00) Dose (mg/day), median (IQR): 150.00 (120.00–175.00) Dose (mg/kg/day), median (IQR): 1.82 (1.51–2.25) Plasma concentration (ng/mL)/dose (mg/kg/day), median (IQR): 52.89 (36.42–69.25) - Tacrolimus, N = 248: Plasma concentration (ng/mL), median (IQR): 7.19 (5.65–8.51) Dose (mg/day), median (IQR): 4.00 (3.00–6.00) Dose (mg/kg/day), median (IQR): 0.06 (0.04–0.08) Plasma concentration (ng/mL)/dose (mg/kg/day), median (IQR): 123.70 (81.32–177.05) - Concomitant treatments, n (%) At least one strong CYP3A4/5 inhibitor: 14 (3.23) At least one weak CYP3A4/5 inhibitor: 5 (1.24) At least one strong CYP3A4/5 inducer: 6 (1.49) At least one weak CYP3A4/5 inducer: 3 (0.74) At least one P-gp inhibitor: 87 (21.34) At least one P-gp inducer: 5 (1.24) At least one drug that binds strongly to plasma proteins: 399 (98.76) **AUTHORS’ KEY FINDINGS:** - Uremic toxins serum concentration, median (inter-quartile range IQR) in Group 1 and Group 2: - *p*CS (μg/mL): 3.02 (1.10–5.65) and 5.80 (2.88–10.63), (*p* < 0.0001) - IxS (μg/mL): 1.39 (0.86–2.21) and 3.11 (2.24–5.45), (*p* < 0.0001) - Tacrolimus concentration-to-dose ratio, but not cyclosporin, is associated with IxS and *p*CS concentrations in multivariate analysis (*p* < 0.05), with the exception that the addition of “age” in multiple regression reduces the strength of association for *p*CS (*p* > 0.05) **Batteux B, et al., 2021 [94]:** **OBJECTIVE:** - To determine the relationships between uremic toxin concentrations at the time of kidney transplantation and osteoporosis or bone marrow density (BMD). **STUDY DESIGN:** Observational **SAMPLE POPULATION (310):** - Age 51.1 ± 12.8 years (mean ± SD), 194 males, ethnic group: Caucasian 292, Black 18, BMI (kg/m^2^): 26.2 ± 4.3, thyroid disorders: 17, prior osteoporotic fractures: 30, family history of fracture of upper extremely femur (FUEF): 2, DM: 50, chronic inflammatory rheumatism: 4, autoimmune diseases: 20, primary hyperparathyroidism (HPT): 8, secondary HPT: 266, smoking: [never 159, current 88, past 63], alcohol consumption: 22, menopausal women: 51, serum calcium (mg/L): 93.8 ± 8.0, serum phosphate (mg/L): 45.3 ± 14.6, serum [25(OH) vitamin D] (ng/mL): 33.4 ± 16.2, serum PTH (pg/mL): 334.0 (1.3–2646.0), serum bone alkaline phosphatases (µg/L): 12.3 (2.4–99.0), serum osteocalcin (ng/mL): 74.7 (2.0–2970.0), serum creatinine at 12 months (mg/L): 16.2 ± 7.5, GFR at 12 months (mL/min): 51.3 ± 20.2, time on hemodialysis (years): 2.5, previous kidney transplant: 1 (37), 2 (6) - Underlying cause of CKD: Glomerulonephritis: 92 Hereditary disease: 58 Polycystic kidney disease: 55 Renal and urinary tract malformations: 29 Hypertensive kidney disease: 24 Diabetic kidney disease: 23 Interstitial nephritis: 13 Vascular nephropathy: 11 Indeterminate: 44 Other: 16 - Maintenance therapy: Mycophenolate (MMF) + tacrolimus: 208 MMF + cyclosporine: 87 Tacrolimus + everolimus: 13 MMF + everolimus: 1 Tacrolimus + azathioprine: 1 Early steroid withdrawal: 41 - Uremic toxin concentrations at transplantation *p*CS (mg/mL or µg/mL on Figures), median (IQR): 16.1 (9.02–25.60) CMPF (mg/mL), median (IQR): 2.57 (0.97–5.08) IxS (mg/mL), median (IQR): 19.80 (12.82–28.32) *p*CG (mg/mL), median (IQR): 0.80 (0.25–1.58) HA (mg/mL), median (IQR): 25.45 (10.90–50.15) TMAO (mg/mL), median (IQR): 4.27 (2.54–7.78) IAA (mg/mL), median (IQR): 0.75 (0.58–1.06) - Occurrence of fractures: Within 12 months of transplantation: 4 Within 24 months of transplantation: 4 **AUTHORS’ KEY FINDINGS:** - IxS, but not *p*CS, correlated with femoral neck and the total hip bone mineral density at 1 month (rho = +0.13, *p* = 0.049 and rho = +0.13, *p* = 0.037, respectively). - IxS and *p*CS concentrations did not change significantly between patients with reduced BMD and patients with increased BMD at month 12. - No clear relationship was observed between uremic toxin serum concentrations at transplantation and osteoporosis or BMD changes up to 24 months post-transplant.	André et al. 2020 [45]
**OBJECTIVE:** - To compare rapid equilibrium dialysis (RED) and ultrafiltration (UF) in the determination of free serum concentrations of uremic toxins. **STUDY DESIGN:** Not applicable **SAMPLE POPULATION (N = NA):** - Serum samples from patients with CKD (N = 30) **AUTHORS’ KEY FINDINGS:** - UF and RED show comparable results for free fraction determination for IxS and *p*CS. **ADDITIONAL PUBLISHED BIOLOGICAL APPLICATIONS CITING THIS ASSAY:** **El Chamieh C, et al., 2023 [95]:** **OBJECTIVE:** - To determine the relationships between proton pump inhibitor (PPI) usage and serum concentrations of uremic toxins. **STUDY DESIGN:** cross-sectional **SAMPLE POPULATION (N = 680):** - CKD patients: median age: 68 years; median eGFR: 32 mL/min/1.73 m^2^. - Group 1: with PPI, N = 211. - Group 2: no PPI, N = 469. Baseline parameters for total population, group 1, and group 2, respectively: - Age (years): 68 [61–77] (median, range), 71 [65–78], and 67 [59–75]. *p* < 0.001. - Women: 31%, 37%, and 28%. *p* = 0.02. - eGFR (mL/min/1.73 m^2^): 32 [23–41], 30 [22–39], and 32 [24–42]. *p* = 0.02. - Albuminuria categories, *p* = 0.38: A1 (Normal to mildly increased): 30%, 27%, and 31% A2 (Moderately increased): 30%, 33.5%, and 28% A3 (Severely increased): 40%, 39.5%, and 41% - History of acute kidney injury: 21%, 28%, and 17.5%. *p* = 0.002. - Smoking status: Never smoker: 38%, 39.5%, and 37.5%. *p* = 0.69. Current smoker: 13%, 13.5%, and 12% Former smoker: 49%, 47%, and 50.50% - HTN: 96.6%, 98%, and 96%. *p* = 0.16. - Diabetes: 40%, 44%, and 38.5%. *p* = 0.16. - Dyslipidemia: 73%, 79.4%, and 69.6%. *p* = 0.008. - History of CVD: 52%, 63.5%, and 47.3%. *p* < 0.001. - Serum albumin (g/L): 40.5 [37.8–43] (median, range), 40 [37.5–42], and 41 [38–43]. *p* = 0.03. - Hemoglobin (g/dl): 13.1 (1.64) (mean ± SD), 12.8 (1.7), and 13.2 (1.5). *p* = 0.002. - High-sensitivity C-reactive protein (mg/L): 2 [1–6], 4 [2–8], and 2 [1–5]. *p* < 0.001. - BMI (kg/m2): 28 [25.2–31.4], 28.5 [25.1–32.4], and 27.8 [25.2–31]. *p* = 0.38. - Total number of medications: 8 [5–10], 10 [8–12], and 7 [4–9]. *p* < 0.001. **AUTHORS’ KEY FINDINGS:** - Patients on PPI had higher concentrations of total and free IxS and *p*CS compared to the no-PPI group. - Multiple linear regression analysis incorporating numerous clinical factors indicated only IxS, but not *p*CS, remain significantly correlated with PPI. **Fabresse N, et al., 2023 [57]:** **OBJECTIVE:** - To explore the associations between the saliva and free serum concentrations for multiple uremic toxins. **STUDY DESIGN:** observational, with simultaneous collection of saliva and serum **SAMPLE POPULATION (N = 18):** - Healthy volunteers **AUTHORS’ KEY FINDINGS:** - The saliva-serum correlation is statistically significant for IxS and *p*CS. - Median salivary concentration (ng/mL): IxS: 7 (2–56), *p*CS: 12 (3–69). - Median serum concentration (ng/mL): IxS: 14 (5–45), *p*CS: 31 (1–67). - Serum saliva ratio: IxS: 2.4 (0.8–4.6), *p*CS: 2.4 (0.4–5.3). - Spearman Correlation Coefficient: IxS (0.78, *p* < 0.0001) and *p*CS (0.68, *p* = 0.002). **Massy ZA, et al., 2021 [96]:** **OBJECTIVE:** - To determine the relationship between uremic toxin concentrations and CKD symptoms in men and women (≥65 years old) at stages 4 and 5 CKD but not on dialysis. **STUDY DESIGN:** Observational. **SAMPLE POPULATION (795):** - CKD patients at stages 4 and 5, not on hemodialysis, 510 males, age ≥ 65 years, eGFR < 20 mL/min/1.73 m^2^ Demographic and clinical characteristics for the total populations, for females and for males, respectively: - Primary renal disease (*p* = 0.02): Glomerular disease: 91, 25, and 66 Tubulointerstitial disease: 79, 40, and 39 Diabetes: 159, 49, and 110 Hypertension: 276, 102, and 174 Miscellaneous renal disorders: 190, 69, and 121 - Weight (kg): 81.47, 74.76, and 85.22 (*p* < 0.001) - BMI (kg/m^2^): 28.60, 29.26, and 28.23 (*p* = 0.01) - eGFR (mL/min/1.73 m^2^): 17.70 ± 5.42 (mean ± SD), 18.47 ± 5.44, and 17.28 ± 5.37 (*p* = 0.003) - SBP (mmHg): 146.49 ± 22.22, 146.35 ± 23.46, and 146.57 ± 21.53 (*p* = 0.90) - DBP (mmHg): 74.72 ± 11.43, 74.07 ± 11.63, and 75.08 ± 11.32 (*p* = 0.23) - Smoking status: Current smokers: 59, 23, and 36 Ex-smoker: 410, 111, and 299 Never: 312, 146, and 166 (*p* < 0.001) - Cholesterol (mM): 4.67 ± 1.35, 4.98 ± 1.44, and 4.49 ± 1.27 (*p* < 0.001) - Hb (g/dL): 11.6 ± 1.5, 11.5 ± 1.5, and 11.6 ± 1.6 (*p* = 0.25) - Calcium (mM): 2.28 ± 0.16, 2.32 ± 0.15, and 2.26 ± 0.15 (*p* < 0.001) - PO_4_ (mM): 1.31 ± 0.33, 1.30 ± 0.30, and 1.31 ± 0.35 (*p* = 0.84) - Albumin (g/dL): 37.56 ± 5.48, 37.73 ± 5.33, and 37.46 ± 5.57 (*p* = 0.51) - Potassium (mM): 4.59 ± 0.60, 4.55 ± 0.60, and 4.61 ± 0.60 (*p* = 0.17) - Sodium (mM): 140.36 ± 3.24, 140.16 ± 3.47, and 140.47 ± 3.11 (*p* = 0.20) - Bicarbonate (mM): 22.48 ± 3.86, 23.28 ± 4.01, and 22.03 ± 3.71 (*p* < 0.001) - Hypertension: 698, 248, and 450 (*p* = 0.70) - Diabetes: 318, 101, and 217 (*p* = 0.06) - Cerebrovascular disease: 121, 41, and 80 (*p* = 0.70) - Peripheral vascular disease: 104, 28, and 76 (*p* = 0.05) - Chronic heart failure: 142, 50, and 92 (*p* = 0.94) - Myocardial infarction: 139, 37, and 102 (*p* = 0.02) - Left ventricular hypertrophy: 141, 41, and 100 (*p* = 0.08) - Atrial fibrillation: 148, 51, and 97 (*p* = 0.77) - Country (*p* = 0.04): Germany: 133, 55, and 78 Poland: 69, 22, and 47 Sweden: 286, 86, and 200 UK: 307, 122, and 185 **AUTHORS’ KEY FINDINGS:** - UT concentrations in total cohort, female, and male participants [median (IQR)]: - IxS (µg/mL): 3.917 (2.204–7.615), 3.440 (1.797–5.708), 4.352 (2.448–8.516) (*p* < 0.001) - *p*CS (µg/mL): 24.441 (16.196–36.951), 21.682 (14.386–32.698), 27.438 (17.106–40.276) (*p* < 0.001) Prevalence of CKD symptoms (%) in total, female, and male participants: - Constipation: 33.5, 41.9, 28.8 (*p* < 0.001) - Decreased appetite: 29.3, 34.2, 26.6 (*p* = 0.03) - Muscle cramps: 57.1, 59.5, 55.8 (*p* = 0.35) - Swelling in legs: 56.5, 63.7, 52.4 (*p* = 0.003) - Shortness of breath: 46.8, 52.5, 43.6 (*p* = 0.02) - Dizziness: 37, 40.8, 34.8 (*p* = 0.11) - Restless legs: 32.3, 41.5, 27.2 (*p* < 0.001) - Tingling in feet: 34.5, 40.5, 31.1 (*p* = 0.01) - Fatigue: 76.2, 79.9, 74.2 (*p* = 0.082) - Cough: 38.6, 38.7, 38.6 (*p* = 0.99) - Dry mouth: 55.8, 62.3, 52.3 (*p* = 0.01) - Bone or joint pain: 58.6, 69.0, 52.8 (*p* < 0.001) - Headache: 25.7, 34.9, 20.5 (*p* < 0.001) - Muscle soreness: 35.6, 42.6, 31.7 (*p* = 0.003) - Dry skin: 57.9, 69.4, 51.5 (*p* < 0.001) - Itching: 54.6, 55.6, 54.0 (*p* = 0.71) - Trouble falling asleep: 40.3, 49.3, 35.2 (*p* < 0.001) - Trouble staying asleep: 54.1, 61.6, 49.9 (*p* = 0.002) - Decreased interest in sex: 58.9, 50.7, 63.4 (*p* = 0.001) - Difficulty becoming sexually aroused: 57.9, 45.4, 64.8 (*p* < 0.001) - Loss of strength: 64.3, 67.6, 62.4 (*p* = 0.17) - *p*CS is associated with constipation in men, odds ratio of 1.46 (*p* < 0.01). - IxS is associated with difficulty becoming sexually aroused in men, odds ratio of 1.34 (*p* < 0.01).	Fabresse et al. 2020 [31]
**OBJECTIVE:** - To evaluate the efficiency of hemodialysis in uremic toxin removal in end-stage renal disease patients (ESRD). **STUDY DESIGN:** Observational. **SAMPLE POPULATION (66):** - CKD patients at ESRD (N = 10), 6 males. - Healthy volunteers (N = 56). - Samples were collected before and after hemodialysis. Patients’ characteristics at diagnosis for Sex, Age (years), Height (cm), Weight (Kg), Creatinine (µM), Cystatin C (mg/L), and Urea nitrogen (µM): - 1: Male, 63, 168, 74, 1182, 8.58, and 29.05 - 2: Male, 42, 175, 68, 760, 7.12, and 23.32 - 3: Female, 52, 162, 42, 883, 7.21, and 28.43 - 4: Female, 50, 166, 56.5, 858, 6.61, and 21.78 - 5: Female, 51, 160, 52.8, 701, 6.25, and 31.53 - 6: Female, 26, 166, 54, 1105, 7.72, and 27.69 - 7: Male, 22, 182, 59.5, 902, 9.6, and 24.21 - 8: Male, 55, 177, 64, 977, 6.2, and 24.21 - 9: Male, 45, 170, 58, 719, 6.18, and 19.88 - 10: Male, 71, 176, 73, 1001, 8.89, and 27.7 **AUTHORS’ KEY FINDINGS:** - Serum concentrations of UTs: normal concentration (µg/mL) (N = 56 healthy volunteers included in this study): IxS: 1.7559 ± 1.2859 *p*CS: 2.269 ± 2.629 - Protein-binding ratio of uremic toxins in vivo and in vitro: IxS: Patients with ESRD in vivo (%) (N = 10): 92 ± 13 *p*CS: Patients with ESRD in vivo (%) (N = 10): 93 ± 3 - According to the following equations: - Efficiency of hemodialysis (%) = (concentration before hemodialysis−concentration after hemodialysis)/concentration before hemodialysis *100% - Accumulation of hemodialysis (fold) = (concentration after hemodialysis−mean concentration of healthy people)/mean concentration of healthy people IxS: efficiency = 24.00 ± 18.29, accumulation = 33.26 ± 18.02 *p*CS: efficiency = 34.28 ± 24.38, accumulation = 33.22 ± 23.09 **[Note: no additional human biological applications were identified]**	Ma et al., 2020 [46]
**OBJECTIVE:** - To assess the concentrations of PBUTs in the serum of chronic kidney disease (CKD) patients on peritoneal dialysis. **STUDY DESIGN:** Observational. **SAMPLE POPULATION (N = 81):** - Patients at CKD 5, on peritoneal dialysis, N = 81, 49 males, age 45.3 (18–77; mean and range), body weight 62.6 (40.7–99.9) kg, dialysis duration 11(1–65) months. Samples were taken pre-dialysis. **AUTHORS’ KEY FINDINGS:** - Large variability of IxS observed (~up to 20 µg/mL). **[Note: no additional human biological applications were identified]**	Wang et al. 2020 [47]
No uremic toxin concentrations or biological findings were reported in Monosik 2016 [49].	Monosık, R. et. al, 2016 [49]
No uremic toxin concentrations or biological findings were reported by Zhu et al., 2011 [32] **ADDITIONAL PUBLISHED BIOLOGICAL APPLICATIONS CITING THIS ASSAY:** **Kuo SZ, et al., 2021 [97]:** **OBJECTIVE:** - To determine the relationship between urinary IxS concentrations and intensive care unit (ICU) patient outcomes. **STUDY DESIGN:** Observational **SAMPLE POPULATION (N = 78):** - Adults ICU patients at admission. ≥18 years old. Urine samples were collected within 4 h of the ICU admission and after 72 h. - Group 1: patients with low IxS concentrations, < 6.9 µmol/mmol creatinine (N = 22). - Group 2: patients with high IxS concentrations (N = 56). - Characteristics at ICU admission for Group 1 and Group 2: - Age, years: tertiles, *p* = 0.64 30–59: 6 (27%) and 20 (36%) 60–70:9 (41%) and 17 (30%) 70–90: 7 (32%) and 19 (34%) - Gender, *p* = 0.02 Male: 9 (41%) and 39 (70%) Female: 13 (59%) and 17 (30%) - Reasons for admission, *p* < 0.01 Sepsis: 17 (77%) and 12 (21%) Surgical procedure: 3 (14%) and 18 (32%) Cardiac: 0 (0%) and 14 (25%) Gl bleed: 2 (9%) and 3 (5%) Neurologic: 0 (0%) and 5 (9%) Other: 0 (0%) and 4 (7%) - Modified Charlson’s Comorbidity Index, *p* = 0.25 0–1 point: 4 (18%) and 21 (37%) 2–3 points: 10 (45%) and 21 (37%) 4+ points: 8 (36%) and 14 (25%) - Sequential organ failure assessment score (SOFA), *p* = 0.50 0–4 points: 9 (41%) and 23 (41%) 5–8 points: 8 (36%) and 14 (25%) ≥9 points: 5 (23%) and 19 (34%) - Hematocrit at admission: 28.8 (24.3–36.7) and 34.5 (28.6–40), *p* = 0.09 - Albumin at admission 3.1 (2.5–3.8) and 3.6 (2.9–3.9), *p* = 0.22 **AUTHORS’ KEY FINDINGS:** - Urinary IxS concentrations, median (IQR): - At ICU admission: 17.1 μmol/mmol (IQR 9.5 to 26.2), N = 78 - At 72 h after ICU: 15.6 μmol/mmol (IQR 4.2 to 30.7), N = 13 - Group 1 represents significantly higher proportions of females - Lower urinary IxS concentrations in ICU patients on immunosuppressants compared to patients not having immunosuppressants during the initial 72 h (*p* = 0.04) - Low urinary IxS at 72 h correlated with reduced ICU-free days (*p* = 0.03) and reduced survival within one year (*p* = 0.09)	Zhu et al. 2011 [32]
**OBJECTIVE(S):** - To quantify serum uremic toxin concentrations in CKD patients not on dialysis. **STUDY DESIGN:** Observational. **SAMPLE POPULATION (N = 19):** - Patients with chronic kidney disease (CKD) at stages 2–5 and not yet on dialysis (N = 19). **AUTHORS’ KEY FINDINGS:** - Concentrations in serum samples from stage 2–5 CKD patients, mean ± SD [minimum–maximum]: - IxS (µM): N = 18, 21.3 ± 20.5 [< LOQ–73.3], 1 sample had a concentration lower than LLOQ, LLOQ = 1.95. Equivalent in µg/mL to 4.54 ± 4.37 [< LOQ–15.63], 3 samples had a concentration lower than LLOQ, LLOQ = 0.42. - *p*CS (µM): N = 16, 29.0 ± 21.4 [< LOQ–80.6], 3 samples had a concentration lower than LLOQ, LLOQ = 2.66. Equivalent in µg/mL to 5.46 ± 4.03 [< LOQ–15.17], 3 samples had a concentration lower than LLOQ, LLOQ = 0.5. **[Note: no additional human biological applications were identified]**	Kanemitsu et al. 2017 [50]
**PLASMA**	
**OBJECTIVE(S):** - To quantify plasma *p*CS concentrations in CKD patients. **STUDY DESIGN:** Observational. **SAMPLE POPULATION (N = 25):** - CKD patients. Samples were processed and analyzed using the developed LCMSMS method and a previously published HPLC method. **AUTHORS’ KEY FINDINGS:** - Plasma *p*CS concentration μg/mL, mean ± SD: Using HPLC: 106.86 ± 44.60 Using LCMS: 192.99 ± 70.26 - UPLC and MRM methods agreement R^2^ = 0.7182 **[Note: no additional human biological applications were identified]**	Cuoghi et al. 2012 [38]
**OBJECTIVE:** - To measure the baseline concentration of IxS in the human plasma of healthy volunteers. **STUDY DESIGN:** Observational. **SAMPLE POPULATION (N = 6):** - Healthy volunteers. **AUTHORS’ KEY FINDINGS:** - IxS concentrations in healthy human plasma: mean 0.5 μg/mL (data provided in the original manuscript in Figure form indicate that IxS concentrations were less than 2 μg/mL in all 6 patients). **[Note: no additional human biological applications were identified]**	Ahmed et al. 2022 [39]
**OBJECTIVE:** - To quantify plasma IxS concentrations in CKD patients and healthy volunteers. **STUDY DESIGN:** Observational. **SAMPLE POPULATION (N = 40):** - The demographic data is provided as mean ± SD [range]. - Group 1: healthy volunteers: N = 11, 8 males, age 24.5 ± 4.4 [21–36], weight (Kg) 61.0 ± 9.6 [46.0–75.0], ALT (IU/L) 17.8 ± 6.7 [8.0–28.0], total bilirubin (mg/dL) 0.71 ± 0.30 [0.30–1.3], serum creatinine (mg/dL) 0.77 ± 0.17 [0.52–1.0] - Group 2: CKD patients at stages 3, 4, and 5 (not on dialysis): N = 15, 13 males, age 44.9 ± 13.0 [27–66], weight (Kg) 66.2 ± 15.7 [39.5–90.6], ALT (IU/L) 17.6 ± 11.0 [5.5–46.2], total bilirubin (mg/dL) 0.55 ± 0.23 [0.29–1.2], serum creatinine (mg/dL) 1.7 ± 0.36 [1.1–2.4] - Group 3: CKD patients at stage 5 (undergoing hemodialysis): N = 14, 12 males, age 48.1 ± 13.8 [31–73], weight (Kg) 64.3 ± 13.3 [38.9–90.0], ALT (IU/L) 7.7 ± 4.0 [3.8–16.4], total bilirubin (mg/dL) 0.47 ± 0.12 [0.27–0.72], serum creatinine (mg/dL) 10.4 ± 3.0 [6.0–17.2] - *p* values: gender 0.604, age <0.0001, weight 0.623, ALT 0.0005, total bilirubin 0.085, serum creatinine <0.0001 **AUTHORS’ KEY FINDINGS:** - IxS concentrations, median (range), *p* < 0.0001 among the three groups: Group 1: 0.6 (0.2–1.8) μg/mL, Group 2: 2.4 (0.3–8.0) μg/mL, Group 3: 21.8 (6.0–43.6) μg/mL. **[Note: no additional human biological applications were identified]**	Oda et al. 2022 [51]
**OBJECTIVE:** - To quantify uremic toxin concentrations in the Indian population in stage 5 pre-hemodialysis (pre-HD), post-hemodialysis (post-HD), and healthy controls. **STUDY DESIGN:** Observational. **SAMPLE POPULATION (N = 164):** - Group 1: HD patients, N = 90, 63 males, age (years) 47.23 ± 12.93, diabetes (DM) = 27, Hypertension (HTN) = 81, HTN duration (months) = 32.68 ± 11.9, HD duration (years) = 2.51 ± 1.89, Creatinine (mg/dL) = 5.77 ± 2.27 (PRE-HD), Urea (mg/dL) = 16.59 ± 3.24 (PRE-HD) - Group 2: healthy controls, N = 74, 40 males, age (years), 48.2 ± 17.14, Creatinine (mg/dL) = 0.85 ± 0.28, Urea (mg/dL) = 10.89 ± 3.66. - *p-*values between Group 1 and Group 2 ≤0.003, except for age *p* = 0.745 **AUTHORS’ KEY FINDINGS:** - Concentrations found in group 1 PRE-HD, group 1 POST-HD, and group 2 (μg/mL), mean ± SD: - IxS: 3.5 ± 1.5, 2.34 ± 0.6, and 0.17 ± 0.05 - *p*CS: 16.68 ± 8.8, 12.59 ± 7.1, and 1.94 ± 1.09 **[Note: no additional human biological applications were identified]**	Ragi, N. et al. 2023 [52]
**OBJECTIVE:** - To quantify tryptophan metabolites in plasma and in gastric juices to aid in the diagnosis of gastric cancer. **STUDY DESIGN:** Observational. **SAMPLE POPULATION (N = 52):** - Group 1: gastric tumor patients had a subtotal or total gastrectomy, N = 35, 18 men, median age of 60 years. - Group 2: patients with gastritis, control, N = 17, 11 men, mean age of 46. - 12 h fasting. **AUTHORS’ KEY FINDINGS:** - Plasma IxS concentrations (*p* = 0.003): Group 1: 2.91 ± 2.28 µM = 0.620 ± 0.49 µg/mL Group 2: 4.94 ± 3.15 µM = 1.053 ± 0.67 µg/mL - IxS was not detected in gastric juices. **[Note: no additional human biological applications were identified]**	Choi et al. 2016 [53]
**Details are mentioned in the serum section.**	Monosık, R. et al. 2016
**Details are mentioned in the serum section.**	Kanemitsu et al. 2017 [50]
**OTHER MATRICES**	
**OBJECTIVE:** - To establish reference ranges of *p*CS concentrations in human urine. **STUDY DESIGN:** Observational. **SAMPLE POPULATION (N = 2000):** - Group 1: men (N = 1019) - Group 2: women (N = 981) - Group 3: age 40–49 (N = 982) - Group 4: age 50–59 (N = 1018) Two samples per participant **AUTHORS’ KEY FINDINGS:** *p*CS concentrations, mean ±SD: Group 1: 63.0 ±47.4 μmol/mmol creatinine and 1002.5 ± 737.1 μmol/24-h. Group 2: 103.1 ±71.2 μmol/mmol creatinine and 1031.8 ± 687.9 μmol/24-h. *p*CS concentration in each group, mean ± SD (median) µg/mL: - Group 1: 0.113 ± 0.092 (0.092) - Group 2: 0.121 ± 0.092 (0.10) - Group 3: 0.118 ± 0.094 (0.099) - Group 4: 0.116 ± 0.090 (0.093) **[Note: no additional human biological applications were identified]**	Wijeyesekera et al. 2012 [54]
**OBJECTIVE:** - To characterize the association between urinary IxS concentrations and autism spectrum disorder (ASD). **STUDY DESIGN:** Observational, controlled with healthy age-matched children. **SAMPLE POPULATION (N = 90):** - Children diagnosed with ASD (N = 61), age [mean ± SD]: 4.5 ± 1.8 years, and healthy children (N = 29) of matched age as a control, age [mean ± SD]: 5.7 ± 2.1 years, all males. - Group 1 (N = 37): children with ASD, < 6 years old, age 4.1 ± 0.8 (mean ± SD) years, the total calibrated Autism Diagnostic Observation Schedule 2nd revision (ADOS-2) score: 7.6 ± 1.8 (mean ± SD). - Group 2 (N = 16): aged-matched children, < 6 years old, age-matched to group 1, age 4.7 ± 0.7 (mean ± SD). - Group 3 (N = 24): children with ASD, 6–10 years, age (7.7 ± 0.9, mean ± SD). The total calibrated score ADOS-2: 7.7 ± 0.9 (mean ± SD). - Group 4 (N = 13): age-matched children, 6–10 years, age-matched to group 3, age 8.2 ± 1.2 (mean ± SD). **AUTHORS’ KEY FINDINGS:** - IxS concentrations in urine (μmol/mmol Cr) (mean ± SD): - Group 1: 37.22 ± 24.38 and group 2: 45.81 ± 22.89, *p* = 0.228 - Group 3: 32.63 ± 10.38 and group 4: 18.95 ± 7.11, *p* = 0.00004 **[Note: no additional human biological applications were identified]**	Olesova et al. 2020 [55]
**Details are mentioned in the serum section.**	Monosık, R. et al. 2016
**Details are mentioned in the serum section.**	Zhu et al. 2011 [32]
**OBJECTIVE:** - To assess the utility of saliva for measuring/monitoring IxS and *p*CS. **STUDY DESIGN:** Observational **SAMPLE POPULATION (N = 70):** - Group 1: healthy volunteers, N = 70, age 16–89 (median = 50), resting saliva, - Group 1.1: age < 30 years, N = 17. - Group 1.2: age 30–60 years, N = 28. - Group 1.3: age > 60 years, N = 25. - Group 1 was further divided into < 65 years old (N = 50) and ≥ 65 years old (N = 20). **AUTHORS’ KEY FINDINGS:** Group 1: < 30 years, 30–60 years, and > 60 years old - IxS (median values): 0.0086 (IQR= 0.0072), 0.0092 (IQR= 0.0074), and 0.012 (IQR= 0.013) μg/mL (*p* = 0.106). - *p*CS (mean ± SD): 0.017 ± 0.014, 0.023 ± 0.021, and 0.075 ± 0.074 μg/mL (*p* < 0.0001). Group 1 (second classification, <65 and ≥ 65 years) - IxS (median values): 0.0094 (IQR = 0.0076) and 0.014 (IQR= 0.019) μg/mL (*p* = 0.0498). - *p*CS (median values): 0.016 (IQR = 0.019) and 0.061 (IQR= 0.065) μg/mL (*p* < 0.0001). **ADDITIONAL PUBLISHED BIOLOGICAL APPLICATIONS CITING THIS ASSAY:** **Korytowska, N.; et al., 2021 [66]** Assay: cited Giebultowicz [56] but analyzed both saliva and serum (not originally validated). **OBJECTIVE:** - To characterize the associations between uremic toxins in saliva and loss/deterioration of graft function (DoGF) in kidney transplant recipients **STUDY DESIGN:** Observational **SAMPLE POPULATION (N = 92):** - Kidney transplant recipients, 55 men - Samples collected at baseline (M1), after 6 months from M1 (M6), and after 7 months from M6 (M12) - Age, median (IQR): M1 (53 (19)), M6 (55 (18)), and M12 (56 (20)) - Smoking, median (IQR): current 13 (14), ex-smoker 32 (35), never 47 (51), passive 16 (17) - Diabetes and hypertension, median (IQR): 18 (20) and 55 (60) - BMI, kg/m^2^: M1 25.9 ± 4.4 (mean ± SD), M6 26.3 ± 4.6, and M12 26.5 ± 4.6 [M1 vs. M12: *p* = 0.00572] - Serum creatinine, mg/dL (median (IQR)): M1: 1.46 (1.03), M6: 1.41 (0.85), and M12: 1.46 (0.88) [M6 vs. M12: *p* = 0.00807] - CKD stage M1, M6, and M12: 1: 9 (10), 9 (12), and 6 (8) [Frequency, (%)] 2: 29 (31), 22 (29), and 24 (33) 3: 30 (33), 27 (35), and 25 (34) 4: 21 (23), 15 (19), and 13 (18) 5: 3 (3), 4 (5), and 5 (7) - The causes of ESRD: glomerulonephritis (40%), diabetic nephropathy (10%), and polycystic kidney disease (28%) - Primary transplantation: N = 87% - Secondary transplantation: N = 12% - Kidneys provided from a deceased donor: 90% **AUTHORS’ KEY FINDINGS:** - eGFR, mL/min/1.73 m^2^, median (IQR): M1: 51 (48), M6: 51 (49), and M12: 48 (45) [M6 vs. M12: *p* = 0.00807] - Biomarkers concentrations at M1, M6, and M12: - salivary IxS, ng/mL: 33 (37), 21 (39), and 27 (39); *p* > 0.05 - serum IxS, ng/mL: 2075 (2788), 1715 (2489), and 1870 (2497); *p* > 0.05 - salivary *p*CS, ng/mL: 57 (139), 46 (86), and 48 (78); *p* > 0.05 - serum *p*CS, ng/mL: 7103 (9651), 4697 (6875), and 4755 (6636); *p* > 0.05 - hemoglobin, g/L: 136 (20), 136 (18), and 134 (17); *p* > 0.05 - proteinuria ≥10 mg/dL: 32 (35), 29 (32), and 22 (24) 7 - glucosuria ≥50 mg/dL: 7 (7.7), 4 (5.3), and 3 (3.3) - Correlations between salivary/serum IxS and *p*CS and eGFR at M1 for all subjects (N = 92), DoGF-Free Group (N = 72), and DoGF Group (N = 20): - serum *p*CS: −0.50 (*p* = 0.00001), −0.56 (*p* < 0.00001), and −0.52 (*p* = 0.01909) - salivary *p*CS: −0.55 (*p* < 0.00001), −0.61 (*p* < 0.00001), and −0.47 (*p* = 0.06582) - serum IxS: −0.78 (*p* < 0.00001), −0.76 (*p* < 0.00001), and −0.71 (*p* = 0.00042) - salivary IxS: −0.76 (*p* < 0.00001), −0.75 (*p* < 0.00001), and −0.62 (*p* = 0.01024) Correlations between salivary and serum concentrations at M1: - IxS: R = 0.81, *p* < 0.00001 - *p*CS: R = 0.92, *p* < 0.00001 - Salivary IxS concentrations (univariate *p* = 0.00682, multivariate *p* = 0.01269) and proteinuria (univariate *p* = 0.00522, multivariate *p* = 0.02048) were identified as independent predictors of deterioration of graft function. Salivary *p*CS is not a significant predictor. **Korytowska N, et al., 2018 [98]:** Assay: cited Giebultowicz [56] with slight changes to ion source gas parameter but analyzed both saliva and serum (not originally validated). **OBJECTIVE:** - To investigate the correlation between salivary and serum concentrations of uremic toxins in CKD patients. **STUDY DESIGN:** Observational with healthy controls **SAMPLE POPULATION (N = 119):** - Group 1: CKD patients at stages 1–5, N = 93 - Group 2: healthy volunteers, N = 26. Population parameters for Group 1 and Group 2, respectively: - Age range (years): 19–90 and 24–76 - Age, mean ± SD (years): 57 ± 17 and 52 ± 15. *p* = 0.15 - Females, N (%): 43 (46) and 12 (46). *p* = 0.99403 - Females age, mean ± SD (years): 54 ± 16 and 54 ± 15. *p* = 0.95796 - Males, N (%): 50 (54) and 14 (54). *p* = 0.99403 - Males age, mean ± SD (years): 60 ± 18 and 51 ± 15. *p* = 0.05533 - Creatinine, mean ± SD (µmol/L): 133 ± 86 and 82 ± 12. *p* = 0.00096 - eGFR value, mean ± SD (mL min^−1^): 58 ± 29 and 77 ± 10. *p* = 0.00017 - Smokers, N (%): 12 (13) and 2 (8). *p* = 0.50091 **AUTHORS’ KEY FINDINGS:** - Free and total concentrations of IxS and *p*CS as median (IQR), μg/mL, in control, CKD stages (1–5 combined), CKD-1, CKD-2, CKD-3, CKD-4, and CKD-5, respectively: *p*CS free, serum: 0.044 (0.059), 0.140 (0.210), 0.072 (0.044), 0.114 (0.088), 0.190 (0.170), 0.460 (0.30), and 0.690 (0.430) *p*CS total, serum: 3.10 (3.60), 7.30 (9.10), 4.50 (3.60), 6.00 (5.40), 8.40 (8.400), 21.00 (16.00), 30.00 (27.00) *p*CS, saliva: 0.019 (0.039), 0.063 (0.068), 0.028 (0.026), 0.040 (0.048), 0.080 (0.094), 0.10 (0.180), and 0.198 (0.058) IxS free, serum: 0.011 (0.016), 0.036 (0.044), 0.016 (0.008), 0.023 (0.025), 0.039 (0.039), 0.110 (0.067), and 0.260 (0.190) IxS total, serum: 1.040 (0.650), 2.00 (2.10), 1.030 (0.610), 1.400 (1.20), 2.20 (1.70), 5.30 (3.60), and 9.00 (14.00) IxS, saliva: 0.008 (0.009), 0.021 (0.029), 0.008 (0.005), 0.012 (0.017), 0.025 (0.022), 0.045 (0.052), and 0.166 (0.061) - *p*-values: Salivary *p*CS between experimental and control group: *p* = 0.00001 Salivary IxS between experimental and control group: *p* = 0.00008 Salivary *p*CS between CKD stages (1–2) and CKD stages (3–5): *p* < 0.00001 Salivary IxS between CKD stages (1–2) and CKD stages (3–5): *p* < 0.00001 Salivary *p*CS between CKD-1 and each of CKD-3, CKD-4, and CKD-5: *p* = 0.00283, 0.00022, and 0.00243, respectively. Salivary *p*CS between CKD-2 and each of CKD-3, CKD-4, and CKD-5: *p* = 0.02402, 0.00178, and 0.01501, respectively. Salivary IxS between CKD-1 and each of CKD-3, CKD-4, and CKD-5: *p* = 0.00511, 0.00002, and 0.00009, respectively. Salivary IxS between CKD-2 and each of CKD-3, CKD-4, and CKD-5: *p* = 0.03064, 0.00007, and 0.00050, respectively. - Spearman’s correlation of IxS and *p*CS between saliva concentrations and serum concentrations: IxS free: 0.81 (*p* < 0.00001) IxS total: 0.76 (*p* < 0.00001) *p*CS free: 0.74 (*p* < 0.00001) *p*CS total: 0.70 (*p* < 0.00001) - Spearman’s correlation of IxS and *p*CS between saliva concentrations and serum concentration concentrations in CKD stages (3–5): IxS free: 0.78 (*p* < 0.00001) IxS total: 0.73 (*p* < 0.00001) *p*CS free: 0.69 (*p* < 0.00001) *p*CS total: 0.66 (*p* < 0.00001) - Correlation factor between saliva toxin concentrations and eGFR: IxS: −0.70 (*p* < 0.00001) *p*CS: −0.61 (*p* < 0.00001)	Giebułtowicz et al. 2016 [56]

AHR: aryl hydrocarbon receptor. AKI: acute kidney injury. AS: arterial stiffness. ATA: asymmetric cellulose triacetate. BMI: body mass index. BUN: blood urea nitrogen. CKD: chronic kidney disease. CKD-EPI: Chronic Kidney Disease Epidemiology Collaboration. CVD: cardiovascular disease. CAS: composite amplitude scores. CAF: cardiovascular autonomic function. CGN: chronic glomerulonephritis. DBP: diastolic blood pressure. DM: diabetes mellitus. eGFR: estimated glomerular filtration rate. ESRD: end-stage renal disease. FUEF: fracture of upper extreme femur. HD: hemodialysis. HDL: high-density lipoprotein. HF: high frequency. HR_DB: heart rate response to deep breathing. HTN: Hypertension. ICU: intensive care unit. IxS: Indoxyl sulfate. LC-MS/MS: liquid chromatography-mass spectrometry. LDL: low-density lipoprotein. LLOQ: lower limit of quantification. LPD: low protein diet. LF: low frequency. MAP: mean arterial pressure. MMF: mycophenolate. N: sample size. ND: non-dialysis. NPA: normal protein diet. NPAs: neuropsychological assessments. *p*CS: *p*-Cresyl sulfate. PD: peritoneal dialysis. PPI: proton pump inhibitor. RED: rapid equilibrium dialysis. SBP: systolic blood pressure. SCr: serum creatinine. SD: standard deviation. SSN: Shen-Shuai-Ning. TF: tissue factor. TMAO: trimethylamine N-oxide. UF: ultrafiltration. UPLC-MS/MS: ultra-performance liquid chromatography-mass spectrometry.

## Data Availability

The data presented in this study are available on Embase, Medline, PubMed, Scopus, and Web of Science.
